# The Heat Asymptotics on Filtered Manifolds

**DOI:** 10.1007/s12220-018-00137-4

**Published:** 2019-01-23

**Authors:** Shantanu Dave, Stefan Haller

**Affiliations:** 1 Faculty of Mathematics, University of Vienna, Oskar-Morgenstern-Platz 1, 1090 Vienna, Austria; 2 Present Address: Department of Mathematics, Hill Center for the Mathematical Sciences, Rutgers University, 110 Frelinghuysen Rd., Piscataway, NJ 08854-8019 USA; 3 Department of Mathematics, University of Vienna, Oskar-Morgenstern-Platz 1, 1090 Vienna, Austria

**Keywords:** Filtered manifold, Hypoelliptic operator, Heat kernel expansion, Zeta function, Non-commutative residue, Generic rank-two distribution in dimension, 58J35 (58A30, 58J20, 58J40, 58J42)

## Abstract

The short-time heat kernel expansion of elliptic operators provides a link between local and global features of classical geometries. For many geometric structures related to (non-)involutive distributions, the natural differential operators tend to be Rockland, hence hypoelliptic. In this paper, we establish a universal heat kernel expansion for formally self-adjoint non-negative Rockland differential operators on general closed filtered manifolds. The main ingredient is the analysis of parametrices in a recently constructed calculus adapted to these geometric structures. The heat expansion implies that the new calculus, a more general version of the Heisenberg calculus, also has a non-commutative residue. Many of the well-known implications of the heat expansion such as, the structure of the complex powers, the heat trace asymptotics, the continuation of the zeta function, as well as Weyl’s law for the eigenvalue asymptotics, can be adapted to this calculus. Other consequences include a McKean–Singer type formula for the index of Rockland differential operators. We illustrate some of these results by providing a more explicit description of Weyl’s law for Rumin–Seshadri operators associated with curved BGG sequences over 5-manifolds equipped with a rank-two distribution of Cartan type.

## Introduction

Many geometric structures related to (non-)involutive distributions can be described in terms of an underlying filtered manifold. These include contact structures, Engel manifolds, and all regular parabolic geometries. Filtered analogues of classical (elliptic) operators are usually hypoelliptic [21]. It is an old dream to link local and global aspects of filtered geometry with the spectrum and index of hypoelliptic operators by studying the short-time asymptotics of heat kernels, similarly to the elliptic case. Active research in this direction started in the 1970s, see [22, 25, 46], and was quite popular in 1980s, see [3, 26]. The main accomplishment had been the development of the Heisenberg calculus [3, 43, 52] and its application to operators in contact and CR geometries. On graded nilpotent Lie groups, the representation theory allowed a harmonic analysis perspective of hypoellipticity [16, 34, 35].

The progress on these problems has been revived by the advent of new tangent groupoid techniques to study analysis on these manifolds [15, 54–57]. When the classical (elliptic) pseudodifferential calculus was described in [23] in a coordinate-free way using the tangent groupoid, it facilitated the construction of a pseudodifferential calculus on general filtered manifolds [56, 57]. This calculus has helped resolve two main hitherto unsurmountable challenges in analysis. It first allowed an explicit handle on the parametrix of a hypoelliptic operator similar to the one familiar from the elliptic case or nilpotent Lie groups as in [16]. The universality of this calculus and the existence of the parametrix [21] provide a clean way to obtain a general short-time heat kernel expansion for a large class of differential operators. Remarkably, this heat expansion has the same structure as the one for elliptic operators, bringing back the old expectation that the analysis should relate local geometric properties to global invariants.

The differential operators we consider satisfy a pointwise Rockland condition, a condition on their non-commutative principal symbols that guarantees that these operators are hypoelliptic. We also present here several consequences of the heat kernel asymptotics, which are well known for elliptic operators, but are not known for Rockland operators in this generality. These include the explicit structure of complex powers of the operators, Weyl’s asymptotic formula for the growth of eigenvalues, the McKean–Singer index formula, the description of a non-commutative residue for Heisenberg calculus, and the construction of a K-homology class associated to Rockland operators.

Let us look at the manifolds, the operators, and their heat kernel expansions in more detail.

### Filtered Manifolds

Filtered manifolds provide a very general setup to study geometry [39, 40]. These geometries include foliations, contact manifolds, Engel structures on 4-manifolds, graded nilpotent Lie groups, and all regular parabolic geometries. The equiregular Carnot–Carathéodory spaces considered in [28] are also filtered manifolds.

A filtered manifold *M* has a naturally associated non-commutative tangent bundle, that is, a simply connected nilpotent Lie group $\mathcal {T}_xM$ attached to each point *x* in *M*. The harmonic analysis of this non-commutative tangent space $\mathcal {T}_xM$ is related to the analysis of differential operators on *M*. An effective way to exhibit this relationship uses the so-called Heisenberg tangent groupoid [15, 57]. The pseudodifferential calculus on filtered manifolds [56] was constructed using the Heisenberg tangent groupoid. It was further studied in [21] where we show that a pointwise Rockland condition implies hypoellipticity. We remark that the idea of Heisenberg calculus is based on the work of Debourd–Skandalis [23], who studied the standard pseudodifferential calculus using Connes’ tangent groupoid construction.

The Heisenberg calculus can be globally defined on a filtered manifold *M* using the Heisenberg tangent groupoid, $\mathbb {T}M$. This is a groupoid that provides a deformation of the pair groupoid $M\times M$ into the non-commutative tangent bundle ${\mathcal {T}}M$, which is obtained by giving

$$\begin{aligned} \mathbb {T}M=\bigl ({\mathcal {T}}M\times \{0\}\bigr )\sqcup \bigl (M\times M\times \mathbb {R}^{\times }\bigr ) \end{aligned}$$

a smooth structure which makes all the groupoid maps smooth. There is an action of $\mathbb {R}^{\times }:=\mathbb {R}{\setminus }\{0\}$ on $\mathbb {T}M$ called the zoom action and the calculus is defined to be those distributions on $M\times M$ that admit an essentially homogeneous extension to $\mathbb {T}M$ with respect to the zoom action.

There is an equivalent local description of the kernels in suitably chosen coordinates. In this paper, we will mostly work with the local version; however, the global description allows a neater formulation of the concept of holomorphic families described below. In the local description, the kernels are described in a tubular neighborhood of the diagonal and the germs of diffeomorphism of these tubular neighborhood to the normal bundle is determined by the Euler-like vector field provided by the filtration [30]. Here, we will stick to the original Heisenberg tangent groupoid description for global invariance of the calculus.

### Rockland Differential Operators

Let us briefly recall the kind of operators studied in this article. These are hypoelliptic operators on filtered manifolds which are elliptic in the Heisenberg calculus described above.

Let *M* be a closed filtered manifold and suppose *E* is a complex vector bundle over *M*. Let *A* be a differential operator acting on sections of *E*. In coordinates adapted to the filtration on *M*, we can assign a (co)symbol to *A* by freezing the coefficients at a point $x\in M$, thereby obtaining a left invariant differential operator $\sigma _x(A)$ on the osculating group $\mathcal {T}_xM$. We require that each $\sigma _x(A)$ satisfies the Rockland condition and furthermore *A* is symmetric and non-negative on $L^2(E)$. Examples of such operators include the sub-Laplacian for many classes of sub-Riemannian geometries, the Rumin–Seshadri operators associated with (ungraded) BGG operators on parabolic geometries, and the square of the Connes–Moscovici transverse signature operator [18]. On trivially filtered manifolds, these operators are elliptic in the usual sense, and on contact or Heisenberg manifolds they are elliptic in the Heisenberg calculus of [3, 43].

### Heat Kernel Asymptotics

In this paper, we study the kernel of $e^{-tA}$ for $t>0$ and the complex powers $A^{-z}$ where $z\in \mathbb {C}$ for a differential operator *A* described in the paragraph above. Rather than using the standard approach by Seeley [50], we follow Beals–Greiner–Stanton [4] who use a Volterra–Heisenberg calculus on $M\times \mathbb {R}$ to describe the heat kernel on CR manifolds, see also [43, Chapter 5]. In our general setting, however, no new calculus has to be developed. Instead, we may regard $M\times \mathbb {R}$ as a filtered manifold such that the heat operator $A+\frac{\partial }{\partial t}$ becomes Rockland on $M\times \mathbb {R}$. The general Rockland theorem established in [21, Theorem 3.13] thus yields a parametrix for the heat operator $A+\frac{\partial }{\partial t}$ in the pseudodifferential operator calculus developed by van Erp and Yuncken in [56]. The asymptotic expansion of its Schwartz kernel along the diagonal immediately yields the heat kernel asymptotics for *A*.

Let us emphasize that this approach to the heat kernel asymptotics seems to only work for Rockland differential operators. For more general pseudodifferential operators *P* on the filtered manifold *M*, the corresponding heat operator $\frac{\partial }{\partial t}+P$ on $M\times \mathbb {R}$ is no longer pseudolocal in general and hence not in the Heisenberg pseudodifferential calculus. This implies that we can only investigate ungraded Rockland sequences in the sense of [21] and, in particular, the results can be applied only to ungraded BGG sequences. For example, the BGG sequences for Cartan geometries of generic rank-2 distributions in dimension five are always ungraded, and we will study them in greater detail here.

We mention here two prominent features of the heat asymptotics of Rockland differential operators.

As is well known in the classical (trivially filtered) case, certain terms in the heat kernel asymptotics of elliptic pseudodifferential operators vanish for differential operators. We show that this continues to hold for Rockland differential operators on filtered manifolds. Their expansion takes a form similar to the expansion of classical elliptic differential operators. In particular, there are no log terms, and every other polynomial term vanishes. To see this, we introduce the class of projective operators, see Definition 2, which have the desired asymptotic expansion and contain the parametrices of Rockland differential operators.

Another key feature of the heat asymptotics of a non-negative elliptic operator is the positivity of its leading term. This term has geometric significance: for instance, the leading term in the heat trace expansion of a Laplace operator encodes the volume of the corresponding Riemannian metric. Let us emphasize that this positivity of the leading term remains true for non-negative Rockland differential operators. In particular, this permits to derive a Weyl law for the eigenvalue asymptotics using a Tauberian theorem.

### Applications

The heat kernel asymptotics of an elliptic operator has many important consequences. We present here several analogous consequences for Rockland differential operators. This requires adapting the results in the context of the Heisenberg pseudodifferential calculus described above.

1. The structure of the complex powers $A^{-z}$ follows via Mellin transform from the heat asymptotics in the usual way. Analogous to the celebrated result of Seeley [50], the complex powers are pseudodifferential operators in the calculus of van Erp and Yuncken [56]. We interpret the notion of a holomorphic family of pseudodifferential operators [43], in terms of an essential homogeneity criterion on the Heisenberg tangent groupoid. As can be expected, $A^{-z}$ is a holomorphic family for every non-negative Rockland differential operator *A*.
2. Taking the trace of a holomorphic family, whenever it is defined, produces a holomorphic function. We will show that the various spectral zeta functions have meromorphic continuation with simple poles. In particular, the algebra of integer-order pseudodifferential operators on a filtered manifold admits a non-commutative residue.
3. Weyl’s law for the eigenvalue asymptotics also follows from the positivity of the constant term in the heat expansion, or from the position and residue at the first pole of the spectral zeta function as usual. We will work out Weyl’s law more explicitly for Cartan geometries in dimension five using suitable geometric choices. Surprisingly, the constant in Weyl’s law is universal, depending only on the irreducible representation defining the BGG sequence.
4. Rockland operators are hypoelliptic and hence on a closed manifold they are Fredholm. In particular, we note two consequences towards the study of their index, namely, the description of the K-homology class associated to them, and a generalization of the McKean–Singer formula.

### Structure of the Paper

The remaining part of the paper is organized as follows. In Sect. 2 we formulate our main results: Theorem 1 on the heat kernel asymptotics and Theorem 2 on the structure of complex powers. Furthermore, we derive several immediate consequences: Corollary 1 on the heat trace expansion, Corollary 2 on the zeta function, Corollary 3 on Weyl’s law, Corollary 4 on the McKean–Singer formula, as well as Corollary 5 on the K-homology class. In Sect. 3, we briefly recall some background for the calculus of pseudodifferential operators on filtered manifolds and introduce the class of projective pseudodifferential operators of integral Heisenberg order, see Definition 2. In Sects. 4 and 5, we present proofs of Theorems 1 and 2 , respectively. In Sect. 6 we discuss holomorphic families of Heisenberg pseudodifferential operators. In Sect. 7, we construct a non-commutative residue, see Corollary 6. In Sect. 8 we will work out Weyl’s law more explicitly for Rumin–Seshadri operators, see Corollary 7, and specialize further to BGG operators on 5-manifolds equipped with a rank-two distribution of Cartan type, see Corollary 8.

We would like to thank an anonymous referee for helpful remarks and another anonymous referee for thorough reading and many useful comments.

## Statement of the Main Results

Recall that a *filtered manifold* [21, 39–41] is a smooth manifold *M* together with a filtration of the tangent bundle *TM* by smooth subbundles,

$$\begin{aligned} TM=T^{-m}M\supseteq \cdots \supseteq T^{-2}M\supseteq T^{-1}M\supseteq T^0M=0, \end{aligned}$$

which is compatible with the Lie bracket of vector fields in the following sense: If $X\in \Gamma ^\infty (T^pM)$ and $Y\in \Gamma ^\infty (T^qM)$ then $[X,Y]\in \Gamma ^\infty (T^{p+q}M)$. Putting $\mathfrak t^pM:=T^pM/T^{p+1}M$, the Lie bracket induces a vector bundle homomorphism $\mathfrak t^pM\otimes \mathfrak t^qM\rightarrow \mathfrak t^{p+q}M$ referred to as *Levi bracket.* This turns the associated graded vector bundle $\mathfrak tM:=\bigoplus _p\mathfrak t^pM$ into a bundle of graded nilpotent Lie algebras called the *bundle of osculating algebras.* The Lie algebra structure on the fiber $\mathfrak t_xM=\bigoplus _p\mathfrak t^p_xM$ depends smoothly on the base point $x\in M$, but is not assumed to be locally trivial. In particular, the Lie algebras $\mathfrak t_xM$ might be non-isomorphic for different $x\in M$.

Using negative degrees, we are following a convention prevalent in parabolic geometry, see [11, 40] for instance. The other convention, where everything is concentrated in positive degrees, is the one that has been adopted in [56, 57].

For real $\lambda \ne 0$, we let $\dot{\delta }_\lambda :\mathfrak tM\rightarrow \mathfrak tM$ denote the grading automorphism given by multiplication with $\lambda ^{-p}$ on the summand $\mathfrak t^pM$. Note that $\dot{\delta }_\lambda$ restricts to a Lie algebra automorphism $\dot{\delta }_{\lambda ,x}\in {{\,\mathrm{Aut}\,}}(\mathfrak t_xM)$ for each $x\in M$. We will denote the *homogeneous dimension* of *M* by

1 $$\begin{aligned} n:=-\sum _{p=-m}^{-1}p\cdot {{\,\mathrm{rank}\,}}(\mathfrak t^pM). \end{aligned}$$

Its fundamental importance stems from the fact that a 1-density $\mu$ on the vector space $\mathfrak t_xM$ scales according to

2 $$\begin{aligned} (\dot{\delta }_{\lambda ,x})_*\mu =|\lambda |^{-n}\mu ,\qquad \lambda \ne 0. \end{aligned}$$

The filtration of the tangent bundle induces a *Heisenberg filtration* on differential operators. More explicitly, if *E* and *F* are two vector bundles over *M*, then a differential operator $\Gamma ^\infty (E)\rightarrow \Gamma ^\infty (F)$ is said to have *Heisenberg order* at most *r* if, locally, it can be written as a finite sum of operators of the form $\Phi \nabla _{X_k}\cdots \nabla _{X_1}$ where $\Phi \in \Gamma ^\infty (\hom (E,F))$, $\nabla$ is a linear connection on *E*, and $X_j\in \Gamma ^\infty (T^{p_j}M)$ such that $-(p_1+\cdots +p_k)\le r$. Denoting the space of differential operators of Heisenberg order at most *r* by $\mathcal {D}\mathcal {O}^r(E,F)$, we obtain a filtration

3 $$\begin{aligned} \Gamma ^\infty (\hom (E,F))=\mathcal {D}\mathcal {O}^0(E,F)\subseteq \mathcal {D}\mathcal {O}^1(E,F)\subseteq \mathcal {D}\mathcal {O}^2(E,F)\subseteq \cdots \end{aligned}$$

which is compatible with composition and taking the formal adjoint. More precisely, if $A\in \mathcal {D}\mathcal {O}^r(E,F)$ and $B\in \mathcal {D}\mathcal {O}^s(F,G)$ where *G* is another vector bundle, then $BA\in \mathcal {D}\mathcal {O}^{s+r}(E,G)$ and $A^*\in \mathcal {D}\mathcal {O}^r(F,E)$. As usual, the formal adjoint is with respect to standard $L^2$ inner products of the form

4 $$\begin{aligned} \langle \!\langle \psi _1,\psi _2\rangle \!\rangle :=\int _Mh(\psi _1(x),\psi _2(x))\mathrm{d}x, \end{aligned}$$

where $\psi _1,\psi _2\in \Gamma _c^\infty (E)$. Here $\mathrm{d}x$ is a volume density on *M* and *h* is a fiberwise Hermitian inner product on *E*. The formal adjoint can be characterized by the equation $\langle \!\langle A^*\phi ,\psi \rangle \!\rangle _E=\langle \!\langle \phi ,A\psi \rangle \!\rangle _F$ for all $\psi \in \Gamma ^\infty _c(E)$ and $\phi \in \Gamma ^\infty _c(F)$.

A differential operator $A\in \mathcal {D}\mathcal {O}^r(E,F)$ has a *Heisenberg principal symbol*, $\sigma ^r_x(A)\in \mathcal {U}_{-r}(\mathfrak t_xM)\otimes \hom (E_x,F_x)$ at every point $x\in M$. Here

$$\begin{aligned} \mathcal {U}_{-r}(\mathfrak t_xM)=\bigl \{\mathbf X\in \mathcal {U}(\mathfrak t_xM)\bigm |\forall \lambda \ne 0:\dot{\delta }_{\lambda ,x}(\mathbf X)=\lambda ^r\mathbf X\bigr \} \end{aligned}$$

denotes the degree $-r$ subspace in the universal enveloping algebra of the graded nilpotent Lie algebra $\mathfrak t_xM$. The Heisenberg principal symbol is compatible with composition and taking the formal adjoint, that is,

5 $$\begin{aligned} \sigma ^{s+r}_x(BA)=\sigma ^s_x(B)\sigma ^r_x(A)\qquad \text {and}\qquad \sigma ^r_x(A^*)=\sigma ^r_x(A)^*, \end{aligned}$$

for all $A\in \mathcal {D}\mathcal {O}^r(E,F)$ and $B\in \mathcal {D}\mathcal {O}^s(F,G)$. Actually, the Heisenberg principal symbol provides a canonical short exact sequence,

$$\begin{aligned} 0\rightarrow \mathcal {D}\mathcal {O}^{r-1}(E,F)\rightarrow \mathcal {D}\mathcal {O}^r(E,F)\xrightarrow {\sigma ^r}\Gamma ^\infty \bigl (\mathcal {U}_{-r}(\mathfrak tM)\otimes \hom (E,F)\big )\rightarrow 0. \end{aligned}$$

In particular, the Heisenberg principal symbol provides a canonical isomorphism between the associated graded of the filtered algebra $\mathcal {D}\mathcal {O}(E)$ with the Heisenberg filtration (3), and the graded algebra $\Gamma ^\infty \bigl (\bigoplus _p\mathcal {U}_p(\mathfrak tM)\otimes {{\,\mathrm{end}\,}}(E)\bigr )$. More details may be found in [41, Sect. 1.2.5].

For $x\in M$ we let $\mathcal {T}_xM$ denote the *osculating group* at *x*, that is, the simply connected nilpotent Lie group with Lie algebra $\mathfrak t_xM$. The individual osculating groups can be put together to form a bundle of nilpotent Lie groups ${\mathcal {T}}M$ over *M* such that the fiberwise exponential map, $\exp :\mathfrak tM\rightarrow {\mathcal {T}}M$, becomes a diffeomorphism of locally trivial bundles over *M*. The scaling automorphisms $\dot{\delta }_{\lambda ,x}\in {{\,\mathrm{Aut}\,}}(\mathfrak t_xM)$ integrate to Lie group automorphisms $\delta _{\lambda ,x}\in {{\,\mathrm{Aut}\,}}(\mathcal {T}_xM)$ which combine to form bundle diffeomorphisms $\delta _\lambda :{\mathcal {T}}M\rightarrow {\mathcal {T}}M$ such that $\delta _\lambda \circ \exp =\exp \circ \dot{\delta }_\lambda$. The Heisenberg principal symbol $\sigma ^r_x(A)$ of an operator $A\in \mathcal {D}\mathcal {O}^r(E,F)$ can be regarded as a left invariant differential operator on $\mathcal {T}_xM$ which is homogeneous of degree *r*. More precisely, regarding $\sigma ^r_x(A):C^\infty (\mathcal {T}_xM,E_x)\rightarrow C^\infty (\mathcal {T}_xM,F_x)$, we have $\sigma ^r_x(A)\circ l_g^*=l_g^*\circ \sigma ^r_x(A)$ and $\sigma ^r_x(A)\circ \delta _{\lambda ,x}^*=\lambda ^r\delta _{\lambda ,x}^*\circ \sigma ^r_x(A)$ for all $x\in M$, $\lambda \ne 0$ and $g\in \mathcal {T}_xM$. Here $l_g^*$ denotes pull back along the left translation, $l_g:\mathcal {T}_xM\rightarrow \mathcal {T}_xM$, $l_g(h):=gh$, and $\delta _{\lambda ,x}^*$ denotes pull back along $\delta _{\lambda ,x}:\mathcal {T}_xM\rightarrow \mathcal {T}_xM$.

A differential operator $A\in \mathcal {D}\mathcal {O}^r(E,F)$ is said to satisfy the *Rockland condition* [44] at $x\in M$ if, for every non-trivial irreducible unitary representation of the osculating group, $\pi :\mathcal {T}_xM\rightarrow U(\mathcal {H})$, on a Hilbert space $\mathcal {H}$, the linear operator $\pi (\sigma ^r_x(A)):\mathcal {H}_\infty \otimes E_x\rightarrow \mathcal {H}_\infty \otimes F_x$ is injective. Here $\mathcal {H}_\infty$ denotes the subspace of smooth vectors in $\mathcal {H}$. An operator is called a *Rockland operator* if it satisfies the Rockland condition at every point $x\in M$. We refer to [21, Sect. 2.3] for more details and references.

According to [21, Theorem 3.13] every Rockland operator $A\in \mathcal {D}\mathcal {O}^r(E,F)$ admits a properly supported left parametrix $B\in \Psi ^{-r}_\text {prop}(F,E)$ such that $BA-{{\,\mathrm{id}\,}}$ is a smoothing operator. Here $\Psi ^{-r}$ denotes the class of *pseudodifferential operators of Heisenberg order*$-r$ which has recently been introduced by van Erp and Yuncken, see [56] and Sect. 3. These are operators whose Schwartz kernels have a wave front set which is contained in the conormal of the diagonal. In particular, their kernels are smooth away from the diagonal. Moreover, their kernels admit an asymptotic expansion along the diagonal with respect to a tubular neighborhood which is adapted to the filtration. In particular, the left parametrix *B* induces a continuous operator $\Gamma ^\infty (F)\rightarrow \Gamma ^\infty (E)$ which extends continuously to a pseudolocal operator on distributional sections, $\Gamma ^{-\infty }(F)\rightarrow \Gamma ^{-\infty }(E)$. Consequently, Rockland operators are *hypoelliptic*, that is, if $\psi$ is a distributional section of *E* such that $A\psi$ is smooth on an open subset *U* of *M*, then $\psi$ was smooth on *U*, cf. [21, Corollary 2.10].

For the remaining part of this section, we will assume *M* to be closed. We consider a Rockland differential operator $A\in \mathcal {D}\mathcal {O}^r(E)$ of Heisenberg order $r\ge 1$ which is formally self-adjoint with respect to an $L^2$ inner product of the form (4), that is, for all $\psi _1,\psi _2\in \Gamma ^\infty (E)$ we have

$$\begin{aligned} \langle \!\langle A\psi _1,\psi _2\rangle \!\rangle =\langle \!\langle \psi _1,A\psi _2\rangle \!\rangle . \end{aligned}$$

### Lemma 1

With the above hypotheses, *A* is essentially self-adjoint with compact resolvent on $L^2(E)$.

### Proof

Indeed, *A* is symmetric with dense domain $\Gamma ^\infty (E)$ and thus closeable. By regularity, the domain of its adjoint coincides with the Heisenberg Sobolev space $H^{r}(E)$, see [21, Corollary 3.24]. To see that this coincides with the domain of the closure, let $\Lambda \in \Psi ^r(E)$ and $\Lambda '\in \Psi ^{-r}(E)$ such that $R=\Lambda '\Lambda -{{\,\mathrm{id}\,}}$ is a smoothing operator.^1^ Given $\phi \in H^r(E)$, choose $\phi _j\in \Gamma ^\infty (E)$ such that $\phi _j\rightarrow \Lambda \phi$ in $L^2(E)$. Since $\Lambda '$ and $A\Lambda '$ are both bounded on $L^2(E)$, see [21, Proposition 3.9(a)], we also have $\Lambda '\phi _j\rightarrow \Lambda '\Lambda \phi$ and $A\Lambda '\phi _j\rightarrow A\Lambda '\Lambda \phi$ in $L^2(E)$. Putting $\psi _j:=\Lambda '\phi _j-R\phi \in \Gamma ^\infty (E)$, we obtain $\psi _j\rightarrow \phi$ and $A\psi _j\rightarrow A\phi$ in $L^2(E)$, hence $\phi$ is in the domain of the closure of *A*. The resolvent of *A* is compact, since $A-z$ has a parametrix in $\Psi ^{-r}(E)$ for every $z\in \mathbb {C}$, and these operators are compact on $L^2(E)$, see [21, Proposition 3.9(b)]. $\square$

Assuming, moreover, that *A* is non-negative, that is,

$$\begin{aligned} \langle \!\langle \psi ,A\psi \rangle \!\rangle \ge 0 \end{aligned}$$

for all $\psi \in \Gamma ^\infty (E)$, the spectral theorem [37, Sect. VI§5.3] permits to construct a *strongly differentiable semi-group*$e^{-tA}$ for $t\ge 0$. More precisely, for each $\psi \in L^2(E)$ the vector $e^{-tA}\psi$ is contained in the domain of *A*, and we have

6 $$\begin{aligned} \tfrac{\partial }{\partial t}e^{-tA}\psi =-Ae^{-tA}\psi \qquad \text {as well as}\qquad \lim _{t\searrow 0}e^{-tA}\psi =\psi . \end{aligned}$$

Since *A* is non-negative, each $e^{-tA}$ is a contraction on $L^2(E)$. According to the Schwartz kernel theorem, it thus has a distributional kernel, $k_t\in \Gamma ^{-\infty }(E\boxtimes E')$. More explicitly, we have

7 $$\begin{aligned} \langle \phi ,e^{-tA}\psi \rangle =\int _{(x,y)\in M\times M}\phi (x)k_t(x,y)\psi (y) \end{aligned}$$

for all $t\ge 0$, $\psi \in \Gamma ^\infty (E)$, and $\phi \in \mathcal {D}(E)=\Gamma ^\infty (E')$. Here $E':=E^*\otimes |\Lambda _M|$ where $|\Lambda _M|$ denotes the bundle of 1-densities on *M* and $\langle -,-\rangle$ denotes the canonical pairing between $\mathcal {D}(E)$ and $\Gamma ^{-\infty }(E)=\mathcal {D}'(E)$. This pairing will also be denoted by $\langle \phi ,\xi \rangle =\int _M\phi \,\xi =\int _{x\in M}\phi (x)\xi (x)$, where $\phi \in \mathcal {D}(E)$ and $\xi \in \Gamma ^{-\infty }(E)$. In particular, the right-hand side in (7) denotes the canonical pairing of $\phi \boxtimes \psi \in \Gamma ^\infty (E'\boxtimes E)=\mathcal {D}(E\boxtimes E')$ with $k_t\in \Gamma ^{-\infty }(E\boxtimes E')=\mathcal {D}'(E\boxtimes E')$.

One main aim of this paper is to establish the following heat kernel asymptotics, generalizing a result of Beals–Greiner–Stanton for CR manifolds [4, Theorems 5.6 and 4.5], see also [43, Theorem 5.1.26 and Proposition 5.1.15].

### Theorem 1

(Heat kernel asymptotics) Let *E* be a vector bundle over a closed filtered manifold *M*. Suppose $A\in \mathcal {D}\mathcal {O}^r(E)$ is a Rockland differential operator of even^2^ Heisenberg order $r>0$ which is formally self-adjoint and non-negative with respect to an $L^2$ inner product of the form (4), that is, $\langle \!\langle A\psi _1,\psi _2\rangle \!\rangle =\langle \!\langle \psi _1,A\psi _2\rangle \!\rangle$ and $\langle \!\langle A\psi ,\psi \rangle \!\rangle \ge 0$, for all $\psi ,\psi _1,\psi _2\in \Gamma ^\infty (E)$. Then $e^{-tA}$ is a smoothing operator for each $t>0$, and the corresponding heat kernels $k_t\in \Gamma ^\infty (E\boxtimes E')$ depend smoothly on $t>0$. Moreover, as $t\searrow 0$, we have an asymptotic expansion

$$\begin{aligned} k_t(x,x)\sim \sum _{j=0}^\infty t^{(j-n)/r}q_j(x), \end{aligned}$$

where $q_j\in \Gamma ^\infty \bigl ({{\,\mathrm{end}\,}}(E)\otimes |\Lambda _M|\bigr )$. More precisely, for every integer *N* we have $k_t(x,x)=\sum _{j=0}^{N-1}t^{(j-n)/r}q_j(x)+O(t^{(N-n)/r})$, uniformly in *x* as $t\searrow 0$. Moreover, $q_j(x)=0$ for all odd *j*, and $q_0(x)>0$ in ${{\,\mathrm{end}\,}}(E_x)\otimes |\Lambda _{M,x}|$ for each $x\in M$.

The terms $q_j\in \Gamma ^\infty \bigl ({{\,\mathrm{end}\,}}(E)\otimes |\Lambda _M|\bigr )$ in Theorem 1 are (in principle) locally computable, they can be read off any parametrix for the heat operator $A+\frac{\partial }{\partial t}$, see Remark 2 for more details. The leading term $q_0(x)$ can also be obtained by evaluating the heat kernel of the Heisenberg principle symbol $\sigma _x^r(A)$ at the point $(o_x,1)\in \mathcal {T}_xM\times (0,\infty )$, see (65). Here $o_x\in \mathcal {T}_xM$ denotes the neutral element of the osculating group.

The spectral theorem also permits to construct complex powers $A^z$ for every $z\in \mathbb {C}$. These are unbounded operators on $L^2(E)$ satisfying $A^{z_1+z_2}=A^{z_1}A^{z_2}$ for all $z_1,z_2\in \mathbb {C}$. The powers are defined such that $A^z$ vanishes on $\ker (A)$ and commutes with the orthogonal projection onto $\ker (A)$. In particular, $A^0$ is the orthogonal projection onto the orthogonal complement of $\ker (A)$, and $A^{-1}$ is the pseudoinverse of *A*. If $z\in \mathbb {N}$, then $A^z$ coincides with the ordinary power, i.e., the *z*-fold product of *A* with itself.

Another main goal of this paper is the following result about the structure of complex powers generalizing [43, Theorems 5.3.1 and 5.3.4].

### Theorem 2

(Complex powers) In the situation of Theorem 1, the complex power $A^{-z}$ is a pseudodifferential operator of Heisenberg order $-zr$ for every $z\in \mathbb {C}$, and these powers constitute a holomorphic family of pseudodifferential operators, see Sect. 6. In particular, the kernel $k_{A^{-z}}(x,y)$ is smooth on $\{x\ne y\}\times \mathbb {C}$ and depends holomorphically on the variable $z\in \mathbb {C}$. If $\mathfrak {R}(z)>n/r$, then $A^{-z}$ has a continuous kernel and its restriction to the diagonal, $k_{A^{-z}}(x,x)$, provides a holomorphic family in $\Gamma ^\infty \bigl ({{\,\mathrm{end}\,}}(E)\otimes |\Lambda _M|\bigr )$ for $\mathfrak {R}(z)>n/r$. This family can be extended meromorphically to the entire complex plane with at most simple poles located at the arithmetic progression $(n-j)/r$ where $j\in \mathbb {N}_0$. If $(n-j)/r\notin -\mathbb {N}_0$, then the residue of $k_{A^{-z}}(x,x)$ at $(n-j)/r$ can be expressed as

8 $$\begin{aligned} {{\,\mathrm{res}\,}}_{z=(n-j)/r}\bigl (k_{A^{-z}}(x,x)\bigr )=\frac{q_j(x)}{\Gamma ((n-j)/r)},\qquad j\in \mathbb {N}_0, \end{aligned}$$

where $q_j\in \Gamma ^\infty \bigl ({{\,\mathrm{end}\,}}(E)\otimes |\Lambda _M|\bigr )$ are as in Theorem 1. Moreover, $k_{A^{-z}}(x,x)$ is holomorphic at the points in $-\mathbb {N}_0$, taking the values

$$\begin{aligned} k_{A^l}(x,x)=(-1)^l\,l!\,q'_{n+rl}(x),\qquad l\in \mathbb {N}_0. \end{aligned}$$

Here $q_j'(x):=q_j(x)$ for $j\ne n$ and $q_n'(x):=q_n(x)-p(x,x)$, where $p\in \Gamma ^\infty (E\boxtimes E')$ denotes the Schwartz kernel of the orthogonal projection onto $\ker (A)$.

Before turning to the proof of Theorems 1 and 2, we will now formulate several immediate corollaries. The next one generalizes [4, Theorem 5.6], see also [43, Proposition 6.1.1].

### Corollary 1

(Heat trace asymptotics) In the situation of Theorem 1, the heat trace admits an asymptotic expansion as $t\searrow 0$,

$$\begin{aligned} {{\,\mathrm{tr}\,}}\bigl (e^{-tA}\bigr )\sim \sum _{j=0}^\infty t^{(j-n)/r}a_j. \end{aligned}$$

More precisely, for each integer *N* we have ${{\,\mathrm{tr}\,}}(e^{-tA})=\sum _{j=0}^{N-1}t^{(j-n)/r}a_j+O(t^{(N-n)/r})$ as $t\searrow 0$. Moreover, $a_j=\int _M{{\,\mathrm{tr}\,}}_E(q_j)$ where $q_j\in \Gamma ^\infty \bigl ({{\,\mathrm{end}\,}}(E)\otimes |\Lambda _M|\bigr )$ is as in Theorem 1, $a_j=0$ for all odd *j*, and $a_0>0$.

### Proof

Since $e^{-tA}$ is a smoothing operator, its trace can be expressed as

$$\begin{aligned} {{\,\mathrm{tr}\,}}\bigl (e^{-tA}\bigr )=\int _{x\in M}{{\,\mathrm{tr}\,}}_E(k_t(x,x)),\qquad t>0. \end{aligned}$$

The asymptotic expansion of ${{\,\mathrm{tr}\,}}(e^{-tA})$ thus follows from the asymptotic expansion for $k_t(x,x)$ in Theorem 1. Clearly, $a_0>0$ since $q_0(x)>0$ at each $x\in M$. $\square$

### Corollary 2

(Zeta function) In the situation of Theorem 1, the complex power $A^{-z}$ is trace class for $\mathfrak {R}(z)>n/r$, and $\zeta (z):={{\,\mathrm{tr}\,}}(A^{-z})$ is a holomorphic function on $\{\mathfrak {R}(z)>n/r\}$. This zeta function can be extended to a meromorphic function on the entire complex plane with at most simple poles located at the arithmetic progression $(n-j)/r$ where $j\in \mathbb {N}_0$. If $(n-j)/r\notin -\mathbb {N}_0$, then the residue of $\zeta (z)$ at $(n-j)/r$ can be expressed as

$$\begin{aligned} {{\,\mathrm{res}\,}}_{z=(n-j)/r}\bigl (\zeta (z)\bigr )=\frac{a_j}{\Gamma ((n-j)/r)},\qquad j\in \mathbb {N}_0, \end{aligned}$$

where $a_j$ are the constants from Corollary 1. Moreover, $\zeta (z)$ is holomorphic at the points in $-\mathbb {N}_0$, taking the values

$$\begin{aligned} \zeta (-l)=(-1)^l\,l!\,a_{n+rl}',\qquad l\in \mathbb {N}_0. \end{aligned}$$

Here $a_j':=a_j$ for $j\ne n$ and $a_n':=a_n-\dim \ker (A)$.

### Proof

For $\mathfrak {R}(z)>n/r$ the operator $A^{-z}$ is trace class since it has a continuous kernel according to Theorem 2. Moreover,

$$\begin{aligned} \zeta (z)=\int _{x\in M}{{\,\mathrm{tr}\,}}_E\bigl (k_{A^{-z}}(x,x)\bigr ) \end{aligned}$$

depends holomorphically on *z* since the kernel $k_{A^{-z}}(x,x)$, considered as a family in $\Gamma ^\infty ({{\,\mathrm{end}\,}}(E)\otimes |\Lambda _M|)$, is holomorphic for $\mathfrak {R}(z)>n/r$. Since $k_{A^{-z}}(x,x)$ can be extended meromorphically to the entire complex plane, the same holds true for $\zeta (z)$. The pole structure, residues, and special values follow immediately from the corresponding statements in Theorem 2. $\square$

The next corollary generalizes [43, Proposition 6.1.2].

### Corollary 3

(Weyl’s eigenvalue asymptotics) In the situation of Theorem 1, the operator *A* is essentially self-adjoint on $L^2(E)$ with compact resolvent. There exists a complete orthonormal system of smooth eigenvectors $\psi _j\in \Gamma ^\infty (E)$ with non-negative eigenvalues $\lambda _j\ge 0$, that is, $A\psi _j=\lambda _j\psi _j$ for all $j\in \mathbb {N}$. Moreover,

9 $$\begin{aligned} {{\,\mathrm{tr}\,}}\bigl (e^{-tA}\bigr )=\sum _{j=1}^\infty e^{-t\lambda _j} \qquad \mathrm{and}\qquad \zeta (z)=\sum _{j=1}^\infty \lambda _j^{-z}, \end{aligned}$$

for $t>0$ and $\mathfrak {R}(z)>n/r$, respectively. Furthermore,

$$\begin{aligned} \sharp \{j\in \mathbb {N}\mid \lambda _j\le \lambda \}\sim \frac{a_0\,\lambda ^{n/r}}{\Gamma (1+n/r)}\qquad {\text {as }} \lambda \rightarrow \infty , \end{aligned}$$

where $a_0>0$ is the constant from Corollary 1.

### Proof

We have already shown that *A* is essentially self-adjoint with compact resolvent, see Lemma 1. It is well known that the spectrum of these operators is discrete [37, Theorem 6.29 in Chapter III§6.8] and real [37, Chapter V§3.5]. Since *A* is non-negative, each eigenvalue has to be non-negative. By hypoellipticity, its eigenfunctions are smooth, see [21, Corollary 2.10]. Since $e^{-tA}$ and $A^{-z}$ are trace class for $t>0$ and $\mathfrak {R}(z)>n/r$, respectively, the expressions (9) follow immediately, see [37, Chapter X§1.4]. Using the Tauberian theorem of Karamata, see [33, Theorem 108] or [51, Problem 14.2], Weyl’s law for the asymptotics of eigenvalues follows from the heat trace asymptotics in Corollary 1. $\square$

To formulate the next corollary, suppose *E* and *F* are two vector bundles over a closed filtered manifold *M* and let $D\in \mathcal {D}\mathcal {O}^k(E,F)$ be a differential operator of Heisenberg order at most $k\ge 1$ such that *D* and $D^*$ are both Rockland. Then *D* induces a Fredholm operator between Heisenberg Sobolev spaces, $D:H_s(E)\rightarrow H_{s-k}(F)$, for every real *s*, see [21, Corollary 3.28]. Moreover, its index does not depend on *s* and can be expressed as

10 $$\begin{aligned} {{\,\mathrm{ind}\,}}(D)=\dim \ker (D)-\dim \ker (D^*). \end{aligned}$$

By hypoellipticity, we have $\ker (D)\subseteq \Gamma ^\infty (E)$ and $\ker (D^*)\subseteq \Gamma ^\infty (F)$, see [21, Corollary 2.10].

Using (5) one readily checks that $D^*D$ and $DD^*$ are differential operators of Heisenberg order at most 2*k* which satisfy the Rockland condition. Clearly, they are formally self-adjoint and non-negative. According to Theorem 1, their heat kernels admit asymptotic expansions as $t\searrow 0$,

$$\begin{aligned} k_t^{D^*D}(x,x)\sim \sum _{j=0}^\infty t^{(n-j)/2k}q_j^{D^*D}(x) \end{aligned}$$

and

$$\begin{aligned} k_t^{DD^*}(x,x)\sim \sum _{j=0}^\infty t^{(n-j)/2k}q_j^{DD^*}(x), \end{aligned}$$

respectively. Here $q_j^{D^*D}\in \Gamma ^\infty ({{\,\mathrm{end}\,}}(E)\otimes |\Lambda _M|)$ and $q_j^{DD^*}\in \Gamma ^\infty ({{\,\mathrm{end}\,}}(F)\otimes |\Lambda _M|)$ denote the local quantities from Theorem 1 for the operators $D^*D$ and $DD^*$, respectively.

### Corollary 4

(McKean–Singer index formula) Let *E* and *F* be two vector bundles over a closed filtered manifold *M*. Moreover, let $D\in \mathcal {D}\mathcal {O}^k(E,F)$ be a differential operator of Heisenberg order at most $k\ge 1$ such that *D* and $D^*$ both satisfy the Rockland condition. Then

11 $$\begin{aligned} {{\,\mathrm{ind}\,}}(D) =a_n^{D^*D}-a_n^{DD^*} \end{aligned}$$

where $a_n^{D^*D}=\int _M{{\,\mathrm{tr}\,}}_E(q_n^{D^*D})$ and $a_n^{DD^*}=\int _M{{\,\mathrm{tr}\,}}_F(q_n^{DD^*})$. In particular, we have ${{\,\mathrm{ind}\,}}(D)=0$ whenever the homogeneous dimension *n* is odd.

### Proof

The argument is the same as in the classical case. As usual,

$$\begin{aligned} \tfrac{\partial }{\partial t}\Bigl ({{\,\mathrm{tr}\,}}\bigl (e^{-tD^*D}\bigr )-{{\,\mathrm{tr}\,}}\bigl (e^{-tDD^*}\bigr )\Bigr ) ={{\,\mathrm{tr}\,}}\bigl (D^*De^{-tD^*D}\bigr )-{{\,\mathrm{tr}\,}}\bigl (DD^*e^{-tDD^*}\bigr ) =0, \end{aligned}$$

for we have $De^{-tD^*D}=e^{-tDD^*}D$ and thus ${{\,\mathrm{tr}\,}}(D^*De^{-tD^*D})={{\,\mathrm{tr}\,}}(DD^*e^{-tDD^*})$. Hence, ${{\,\mathrm{tr}\,}}\bigl (e^{-tD^*D}\bigr )-{{\,\mathrm{tr}\,}}\bigl (e^{-tDD^*}\bigr )$ is constant in *t* and Corollary 1 yields

12 $$\begin{aligned} {{\,\mathrm{tr}\,}}\bigl (e^{-tD^*D}\bigr )-{{\,\mathrm{tr}\,}}\bigl (e^{-tDD^*}\bigr )=a_n^{D^*D}-a_n^{DD^*}, \end{aligned}$$

for all $t>0$. On the other hand, $e^{-tD^*D}$ converges to the orthogonal projection onto $\ker (D^*D)$ with respect to the trace norm, as $t\rightarrow \infty$. This follows from Weyl’s law in Corollary 3 or, more directly, from Lemma 5. Hence,

13 $$\begin{aligned} \lim _{t\rightarrow \infty }{{\,\mathrm{tr}\,}}\bigl (e^{-tD^*D}\bigr )=\dim \ker (D^*D)=\dim \ker (D). \end{aligned}$$

Analogously, $e^{-tDD^*}$ converges to the orthogonal projection onto $\ker (DD^*)$ and

14 $$\begin{aligned} \lim _{t\rightarrow \infty }{{\,\mathrm{tr}\,}}\bigl (e^{-tDD^*}\bigr )=\dim \ker (DD^*)=\dim \ker (D^*). \end{aligned}$$

Combining (13) and (14) with (10), we obtain

$$\begin{aligned} \lim _{t\rightarrow \infty }\Bigl ({{\,\mathrm{tr}\,}}\bigl (e^{-tD^*D}\bigr )-{{\,\mathrm{tr}\,}}\bigl (e^{-tDD^*}\bigr )\Bigr )={{\,\mathrm{ind}\,}}(D). \end{aligned}$$

Combining this with (12), we obtain the McKean–Singer index formula (11). If *n* is odd, then $a_n^{D^*D}=0=a_n^{DD^*}$ according to Corollary 1, whence ${{\,\mathrm{ind}\,}}(D)=0$. $\square$

According to Atiyah [2] elliptic differential operators represent K-homology classes of the underlying manifold, see also [36, 6, Sect. 17], or [45, Sect. 5]. We have the following generalization for Rockland differential operators.

### Corollary 5

(K-homology class) Let *E* and *F* be two vector bundles over a closed filtered manifold *M*. Moreover, let $D\in \mathcal {D}\mathcal {O}^k(E,F)$ be a differential operator of Heisenberg order at most $k\ge 1$ such that *D* and $D^*$ both satisfy the Rockland condition. Then the following holds true:

- $P:=D({{\,\mathrm{id}\,}}_E+D^*D)^{-1/2}=({{\,\mathrm{id}\,}}_F+DD^*)^{-1/2}D$ is bounded from $L^2(E)$ to $L^2(F)$.
- $P^*P-{{\,\mathrm{id}\,}}_E$ is compact on $L^2(E)$.
- $PP^*-{{\,\mathrm{id}\,}}_F$ is compact on $L^2(F)$.
- [*f*, *P*] is compact from $L^2(E)$ to $L^2(F)$, for each $f\in C^\infty (M,\mathbb {C})$.

Hence, the operator $$\begin{pmatrix}0&{}P^*\\ P&{}0\end{pmatrix}$$ acting on $L^2(E)\oplus L^2(F)$ together with the action of the $C^*$-algebra *C*(*M*) by multiplication constitutes a graded Fredholm module, representing a K-homology class in $K_0(M)=KK(C(M),\mathbb {C})$.

### Proof

According to Theorem 2 we have $({{\,\mathrm{id}\,}}_E+D^*D)^{-1/2}\in \Psi ^{-k}(E)$, and thus $P\in \Psi ^0(E,F)$. Hence, *P* represents a bounded operator $L^2(E)\rightarrow L^2(F)$, see [21, Proposition 3.9(a)]. This shows (a).

Clearly, $P^*P-{{\,\mathrm{id}\,}}_E=-({{\,\mathrm{id}\,}}_E+D^*D)^{-1}\in \Psi ^{-2k}(E)$. Hence, $P^*P-{{\,\mathrm{id}\,}}_E$ represents a compact operator on $L^2(E)$, see [21, Proposition 3.9(b)]. This shows (b), and (c) can be proved analogously.

To see (d), we write

$$\begin{aligned}{}[f,P]=[f,D]({{\,\mathrm{id}\,}}_E+D^*D)^{-1/2}+D[f,({{\,\mathrm{id}\,}}_E+D^*D)^{-1/2}]. \end{aligned}$$

The first summand is contained in $\Psi ^{-1}(E,F)$, for we have $[f,D]\in \Psi ^{k-1}(E,F)$ and $({{\,\mathrm{id}\,}}_E+D^*D)^{-1/2}\in \Psi ^{-k}(E)$. The second summand is in $\Psi ^{-1}(E,F)$ too, for $[f,({{\,\mathrm{id}\,}}_E+D^*D)^{-1/2}]\in \Psi ^{-k-1}(E)$. Hence $[f,P]\in \Psi ^{-1}(E,F)$, and thus [*f*, *P*] is compact, see [21, Proposition 3.9(b)]. $\square$

## Pseudodifferential Operators on Filtered Manifolds

In this section, we will briefly recall van Erp and Yuncken’s pseudodifferential operator calculus on filtered manifolds, see [56] and [21]. Moreover, we will introduce a subclass of operators of integral order, characterized by an additional symmetry, and containing all differential operators. This class will be used to show that the heat kernel expansion for a differential operator has no log terms, and every other polynomial term vanishes.

Let *M* be a filtered manifold. For any two complex vector bundles *E* and *F* over *M*, and every complex number *s*, there is a class of operators called *pseudodifferential operators of Heisenberg order s* and denoted by $\Psi ^s(E,F)$, mapping sections of *E* to sections of *F*. Every $A\in \Psi ^s(E,F)$ has a distributional Schwartz kernel $k\in \Gamma ^{-\infty }(F\boxtimes E')$ with wave front set contained in the *conormal* of the diagonal. In particular, *k* is smooth away from the diagonal and *A* induces a continuous operator $\Gamma ^\infty _c(E)\rightarrow \Gamma ^\infty (F)$ which extends continuously to a pseudolocal operator on distributional sections, $\Gamma ^{-\infty }_c(E)\rightarrow \Gamma ^{-\infty }(F)$. Here $E':=E^*\otimes |\Lambda _M|$ where $E^*$ denotes the dual bundle and $|\Lambda _M|$ is the line bundle of 1-densities on *M*. Moreover, $F\boxtimes E'=p_1^*F\otimes p_2^*E'$ where $p_i:M\times M\rightarrow M$ denote the canonical projections, $i=1,2$. As usual,

$$\begin{aligned} \langle \phi ,A\psi \rangle =\int _{(x,y)\in M\times M}\phi (x)k(x,y)\psi (y) \end{aligned}$$

for all $\psi \in \Gamma ^\infty _c(E)$ and $\phi \in \mathcal {D}(F)=\Gamma ^\infty _c(F')$ where $\langle -,-\rangle$ denotes the canonical pairing between $\mathcal {D}(F)=\Gamma ^\infty _c(F')$ and $\Gamma ^\infty (F)$. We will denote the space of these conormal kernels by $\mathcal {K}(M\times M;E,F)$. Moreover, we introduce the notation $\mathcal {K}^\infty (M\times M;E,F):=\Gamma ^\infty (F\boxtimes E')$ for the subspace of smooth kernels corresponding to smoothing operators, denoted by $\mathcal {O}^{-\infty }(E,F)$. The operators in the class $\Psi ^s(E,F)$ can be characterized by having a conormal kernel which admits an *asymptotic expansion along the diagonal* in carefully chosen coordinates.

The asymptotic expansion of the kernels can be formulated using tubular neighborhoods of the diagonal adapted to the filtration of *M*. These are constructed using two geometrical choices: (1) a splitting of the filtration on *TM*, i.e., a vector bundle isomorphism $S:\mathfrak tM\rightarrow TM$ mapping $\mathfrak t^pM$ into $T^pM$ such that the composition with the canonical projection $T^pM\rightarrow T^pM/T^{p+1}M=\mathfrak t^pM$ is the identity on $\mathfrak t^pM$; and (2) a linear connection $\nabla$ on *TM* preserving the decomposition $TM=\bigoplus _pS(\mathfrak t^pM)$. Denoting the corresponding exponential map by $\exp ^\nabla :TM\rightarrow M$, we obtain a commutative diagram:15Here $\exp :\mathfrak tM\rightarrow {\mathcal {T}}M$ denotes the fiberwise exponential map, all downwards heading vertical arrows indicate canonical bundle projections, and the upwards pointing vertical arrows denote the corresponding zero (neutral) sections. In particular, $\Delta (x)=(x,x)$ denotes the diagonal mapping, and ${{\,\mathrm{pr}\,}}_1(x,y)=x$. Using $-S$ to identify $\mathfrak tM$ with *TM*, mediates between two common, yet conflicting, conventions we have adopted: The Lie algebra of a Lie group is defined using *left* invariant vector fields, while the Lie algebroid of a smooth Lie groupoid is defined using *right* invariant vector fields.

Restricting the composition in the top row of diagram (15) to a sufficiently small neighborhood *U* of the zero section in ${\mathcal {T}}M$, it gives rise to a diffeomorphism $\varphi :U\rightarrow V$ onto an open neighborhood *V* of the diagonal in $M\times M$ such that the rectangle at the bottom of diagram (16) commutes:16Possibly shrinking *U*, there exists a vector bundle isomorphism $\phi$ over $\varphi$ which restricts to the tautological identification over the diagonal/zero section,

17 $$\begin{aligned} o^*\bigl (\hom (\pi ^*E,\pi ^*F)\otimes \Omega _\pi \bigr )=\hom (E,F)\otimes |\Lambda _M|=\Delta ^*(F\boxtimes E'), \end{aligned}$$

and such that the upper rectangle in diagram (16) commutes. Here $\Omega _\pi$ is the line bundle over ${\mathcal {T}}M$ obtained by applying the representation $|{\det }|$ to the frame bundle of the vertical bundle $\ker (T\pi )$. Note that the restriction $\Omega _\pi |_{\mathcal {T}_xM}$ is canonically isomorphic to the 1-density bundle $|\Lambda _{\mathcal {T}_xM}|$ over $\mathcal {T}_xM$. Moreover, we have a canonical identification $o^*\Omega _\pi =|\Lambda _{\mathfrak tM}|=|\Lambda _{TM}|$ used in (17). Every pair $(\varphi ,\phi )$ as above will be referred to as *exponential coordinates adapted to the filtration.* Only the germ of $(\varphi ,\phi )$ along the zero section is relevant for formulating the asymptotic expansion. We will occasionally suppress the restriction to the neighborhoods *U* or *V* in our notation.

Put $\mathcal {K}^\infty ({\mathcal {T}}M;E,F):=\Gamma ^\infty \bigl (\hom (\pi ^*E,\pi ^*F)\otimes \Omega _\pi \bigr )$, and let $\mathcal {K}({\mathcal {T}}M;E,F)$ denote the space of distributional sections of $\hom (\pi ^*E,\pi ^*F)\otimes \Omega _\pi$ with wave front set contained in the conormal of the zero section *o*(*M*). In particular, elements in $\mathcal {K}({\mathcal {T}}M;E,F)$ are assumed to be smooth away from the zero section. Equivalently, these can be characterized as families $a_x\in \Gamma ^{-\infty }(|\Lambda _{\mathcal {T}_xM}|)\otimes \hom (E_x,F_x)$ which are smooth away from the origin (regular) and depend smoothly on $x\in M$.

Let *s* be a complex number. An element $a\in \mathcal {K}({\mathcal {T}}M;E,F)$ is called *essentially homogeneous of order s* if $(\delta _\lambda )_*a=\lambda ^sa$ mod $\mathcal {K}^\infty ({\mathcal {T}}M;E,F)$, for all $\lambda >0$. The space of principal cosymbols of order *s* will be denoted by

$$\begin{aligned} \Sigma ^s(E,F):=\left\{ a\in \frac{\mathcal {K}({\mathcal {T}}M;E,F)}{\mathcal {K}^\infty ({\mathcal {T}}M;E,F)}:(\delta _\lambda )_*a=\lambda ^sa\, \hbox {for all }\lambda >0\right\} , \end{aligned}$$

cf. [56, Definition 34]. Our notation will often not distinguish between elements in $\Sigma ^s(E,F)$ and the distributions in $\mathcal {K}({\mathcal {T}}M;E,F)$ representing them. Operators in $\Psi ^s(E,F)$ can be characterized as those having a conormal Schwartz kernel $k\in \mathcal {K}(M\times M;E,F)$ which admits an asymptotic expansion of the form

18 $$\begin{aligned} \phi ^*(k|_V)\sim \sum _{j=0}^\infty k_j, \end{aligned}$$

where $k_j\in \Sigma ^{s-j}(E,F)$. More precisely, for every integer *N* there exists an integer $j_N$ such that $\phi ^*(k|_V)-\sum _{j=0}^{j_N}k_j$ is of class $C^N$. Strictly speaking, the right-hand side of (18) involves distributions representing the classes $k_j\in \Sigma ^{s-j}(E,F)$, restricted to *U*. Clearly, the condition expressed in (18) does not depend on the choice of these representatives. We will continue to suppress this in our notation in similar formulas below.

It is a non-trivial fact that a kernel which admits an asymptotic expansion as above, also has an asymptotic expansion of the same form with respect to any other exponential coordinates adapted to the filtration, see [56] and [21, Remark 3.7]. Moreover, the leading term, $\sigma ^s(A):=k_0\in \Sigma ^s(E,F)$, is independent of the exponential coordinates and referred to as *Heisenberg principal symbol* of $A\in \Psi ^s(E,F)$. It provides a short exact sequence

$$\begin{aligned} 0\rightarrow \Psi ^{s-1}(E,F)\rightarrow \Psi ^s(E,F)\xrightarrow {\sigma ^s}\Sigma ^s(E,F)\rightarrow 0. \end{aligned}$$

The basic properties of the class $\Psi ^s(E,F)$ and the Heisenberg principal symbol have been established in [56] and are summarized in [21, Proposition 3.4]. Let us mention a few. If $A\in \Psi ^s(E,F)$ and $B\in \Psi ^r(F,G)$, then $BA\in \Psi ^{r+s}(E,G)$ and $\sigma ^{r+s}(BA)=\sigma ^r(B)\sigma ^s(A)$, provided one of the two operators is properly supported. Here the multiplication of cosymbols, $\Sigma ^r(F,G)\times \Sigma ^s(E,F)\rightarrow \Sigma ^{r+s}(E,G)$ is by fiberwise convolution on ${\mathcal {T}}M$. Moreover, $A^t\in \Psi ^s(F',E')$ and $\sigma ^s(A^t)=\sigma ^s(A)^t$ where $A^t$ denotes the formal transposed operator, and the transposed of $\sigma ^s(A)$ is defined using the (fiberwise) inversion on ${\mathcal {T}}M$. Assuming $r\in \mathbb {N}_0$, a differential operator is contained in $\Psi ^r(E,F)$ if and only if it has Heisenberg order at most *r* in the sense of Sect. 2, and in this case the Heisenberg principal symbol discussed in Sect. 2 is related to the one introduced in the preceding paragraph by the canonical inclusion

$$\begin{aligned} \Gamma ^\infty \bigl (\mathcal {U}_{-r}(\mathfrak tM)\otimes \hom (E,F)\bigr )\subseteq \Sigma ^r(E,F). \end{aligned}$$

We have $\bigcap _{j=0}^\infty \Psi ^{s-j}(E,F)=\mathcal {O}^{-\infty }(E,F)$, where the right-hand side denotes the smoothing operators. The calculus is asymptotically complete. Hence, an operator $A\in \Psi ^s(E,F)$ admits a left parametrix $B\in \Psi ^{-s}_\text {prop}(F,E)$, that is to say, $BA-{{\,\mathrm{id}\,}}$ is a smoothing operator, if and only if $\sigma ^s(A)$ admits a left inverse $b\in \Sigma ^{-s}(F,E)$, that is, $b\sigma ^s(A)=1$ in $\Sigma ^0(E,E)$, see [56, Theorem 60]. The general Rockland theorem in [21, Theorem 3.13] asserts that such a left inverse *b* for the principal symbol exists if and only if $\sigma ^s_x(A)$ satisfies the Rockland condition at each point $x\in M$. The operator class $\Psi ^s$ gives rise to a Heisenberg Sobolev scale with the expected mapping properties, see [21, Proposition 3.21] for details.

A more intrinsic characterization of $\Psi ^s(E,F)$ can be given in terms of the tangent groupoid associated with a filtered manifold, see [15, 56, 57]. This is a smooth groupoid with space of units $M\times \mathbb {R}$ and arrows

$$\begin{aligned} \mathbb {T}M=\bigl (\mathcal {T}^\text {op}M\times \{0\}\bigr )\sqcup \bigl (M\times M\times \mathbb {R}^\times \bigr ), \end{aligned}$$

defined such that the inclusions ${{\,\mathrm{inc}\,}}_0:\mathcal {T}^\text {op}M\rightarrow \mathbb {T}M$ and ${{\,\mathrm{inc}\,}}_t:M\times M\rightarrow \mathbb {T}M$ for $t\ne 0$ are smooth maps of groupoids. Here we use the notation $\mathbb {R}^\times :=\mathbb {R}{\setminus }\{0\}$. As with $-S$ in diagram (15), the opposite groupoid $\mathcal {T}^\text {op}M$ resolves two conflicting, yet common, conventions we are following, one for Lie algebras of Lie groups, and another for Lie algebroids of smooth groupoids. The smooth structure on $\mathbb {T}M$ can be characterized using adapted exponential coordinates, see [57, Theorem 16]. Indeed, if $\varphi :U\rightarrow V$ is as above, then

$$\begin{aligned} \mathcal {T}^\text {op}M\times \mathbb {R}\supseteq \mathbb {U}\xrightarrow {\Phi }\mathbb {V}\subseteq \mathbb {T}M, \qquad \Phi (g,t):={\left\{ \begin{array}{ll}(g,0)&{}\text {if }t=0, \text {and}\\ \bigl (\varphi (\delta _t(\nu (g))),t\bigr )&{}\text {if } t\ne 0,\end{array}\right. } \end{aligned}$$

is a diffeomorphism from the open subset

$$\begin{aligned} \mathbb {U}:=\bigl (\mathcal {T}^\text {op}M\times \{0\}\bigr )\cup \bigl \{(g,t)\in \mathcal {T}^\text {op}M\times \mathbb {R}^\times :\delta _t(\nu (g))\in U\bigr \} \end{aligned}$$

onto the open subset $\mathbb {V}:=\bigl (\mathcal {T}^\text {op}M\times \{0\}\bigr )\cup \bigl (V\times \mathbb {R}^\times \bigr )$. Here $\nu$ denotes the inversion on the osculating groupoid, i.e., the fiberwise inversion on ${\mathcal {T}}M$, and may be regarded as an isomorphism of smooth groupoids, $\nu :\mathcal {T}^\text {op}M\rightarrow {\mathcal {T}}M$, which intertwines the dilation $\delta _\lambda ^\text {op}$ on $\mathcal {T}^\text {op}M$ with the dilation $\delta _\lambda$ on ${\mathcal {T}}M$. The group of automorphisms $\delta _\lambda ^\text {op}$ of $\mathcal {T}^\text {op}M$ extends to a group of automorphisms of the tangent groupoid called the *zoom action* by putting $\delta _\lambda ^{\mathbb {T}M}(g,0):=(\delta ^\text {op}_\lambda (g),0)$ and $\delta _\lambda ^{\mathbb {T}M}(x,y,t):=(x,y,t/\lambda )$ for $t\in \mathbb {R}^\times$ and all $\lambda \ne 0$, see [56, Definition 17].

For two vector bundles *E* and *F* over *M*, we consider the vector bundle

19 $$\begin{aligned} \hom \bigl (\sigma ^*(E\times \mathbb {R}),\tau ^*(F\times \mathbb {R})\bigr )\otimes \Omega _\tau \end{aligned}$$

over $\mathbb {T}M$, where $\sigma ,\tau :\mathbb {T}M\rightarrow M\times \mathbb {R}$ denote the source and target maps given by $\sigma (g,0)=(\pi (g),0)=\tau (g,0)$, $\sigma (x,y,t)=(y,t)$, and $\tau (x,y,t)=(x,t)$ where $g\in \mathcal {T}^\text {op}M$, $t\in \mathbb {R}^\times$, and $x,y\in M$. Moreover, $\Omega _\tau$ denotes the line bundle obtained by applying the representation $|{\det }|$ to the frame bundle of the vertical bundle of $\tau$. We let $\mathcal {K}^\infty (\mathbb {T}M;E,F)$ denote the space of smooth sections of the vector bundle (19), and we let $\mathcal {K}(\mathbb {T}M;E,F)$ denote the space of distributional sections of (19) with wave front set conormal to the space of units in $\mathbb {T}M$. The inclusions and the scaling automorphisms give rise to a commutative diagram, $t\in \mathbb {R}^\times$ and $\lambda \ne 0$,20in which all maps are multiplicative, that is to say, compatible with the convolution and transposition induced by the groupoid structures on $\mathcal {T}^\text {op}M$, $\mathbb {T}M$, and $M\times M$, respectively.

A conormal kernel $k\in \mathcal {K}(M\times M;E,F)$ corresponds to an operator in $\Psi ^s(E,F)$ if and only if it admits an extension across the tangent groupoid which is essentially homogeneous of order *s*. More precisely, iff there exists $\mathbb {K}\in \mathcal {K}(\mathbb {T}M;E,F)$ such that ${{\,\mathrm{ev}\,}}_1(\mathbb {K})=k$ and $(\delta ^{\mathbb {T}M}_\lambda )_*\mathbb {K}=\lambda ^s\mathbb {K}$ mod $\mathcal {K}^\infty (\mathbb {T}M;E,F)$ for all $\lambda >0$, see [56, Definition 19] or [21, Definition 3.2]. In this case, we have $\sigma ^s(A)=\nu ^*({{\,\mathrm{ev}\,}}_0(\mathbb {K}))$.

To express this more succinctly, let us introduce the notation

$$\begin{aligned} \Sigma ^s(\mathbb {T}M;E,F):=\left\{ \mathbb {K}\in \frac{\mathcal {K}(\mathbb {T}M;E,F)}{\mathcal {K}^\infty (\mathbb {T}M;E,F)}:(\delta ^{\mathbb {T}M}_\lambda )_*\mathbb {K}=\lambda ^s\mathbb {K}\, \hbox {for all }\lambda >0\right\} \end{aligned}$$

and

$$\begin{aligned} \Sigma ^s(\mathcal {T}^\text {op}M;E,F):=\left\{ a\in \frac{\mathcal {K}(\mathcal {T}^\text {op}M;E,F)}{\mathcal {K}^\infty (\mathcal {T}^\text {op}M;E,F)}:(\delta ^\text {op}_\lambda )_*a=\lambda ^sa\text { for all } \lambda >0\right\} . \end{aligned}$$

Then we have the following commutative diagram:21The bottom row in (21) is exact in view of [56, Lemma 36 and Proposition 37]. The vertical arrow labeled ${{\,\mathrm{ev}\,}}_1$ is onto by definition of the class $\Psi ^s(E,F)$. It follows from [56, Lemma 32 and Proposition 37] that these vertical arrows are injective too. The arrow labeled *t* in (21) is induced by multiplication with the function $t:\mathbb {T}M\rightarrow \mathbb {R}$ given by the composition of the source (or target) map $\mathbb {T}M\rightarrow M\times \mathbb {R}$ with the canonical projection onto $\mathbb {R}$. Note that

22 $$\begin{aligned} {{\,\mathrm{ev}\,}}_1(t\mathbb {K})={{\,\mathrm{ev}\,}}_1(\mathbb {K}) \qquad \text {and}\qquad \bigl (\delta ^{\mathbb {T}M}_\lambda \bigr )_*(t\mathbb {K})=\lambda t\bigl (\delta _\lambda ^{\mathbb {T}M}\bigr )_*\mathbb {K} \end{aligned}$$

for all $\lambda \ne 0$ and $\mathbb {K}\in \mathcal {K}(\mathbb {T}M;E,F)$. These facts permit to define the principal symbol map $\sigma ^s$ such that the diagram becomes commutative. Moreover, the exactness of the sequence in the top row of (21) follows from the exactness of the sequence at the bottom.

By conormality, $\mathbb {K}$ has a Taylor expansion along $\mathcal {T}^\text {op}M\times \{0\}$

23 $$\begin{aligned} \Phi ^*\bigl (\mathbb {K}|_{\mathbb {V}}\bigr )\sim \sum _{j=0}^\infty \mathbb {K}_jt^j \end{aligned}$$

where $\mathbb {K}_j\in \Sigma ^{s-j}(\mathcal {T}^\text {op}M;E,F)$ and $\mathbb {K}_jt^j$ is considered as a distribution on $\mathcal {T}^\text {op}M\times \mathbb {R}$. More precisely, for each integer *N* there exists an integer $j_N$ such that $\Phi ^*\bigl (\mathbb {K}|_{\mathbb {V}}\bigr )-\sum _{j=0}^{j_N}\mathbb {K}_jt^j$ is of class $C^N$. The coefficients are related to the terms in the asymptotic expansion (18) of $k={{\,\mathrm{ev}\,}}_1(\mathbb {K})$ via

24 $$\begin{aligned} k_j=\nu ^*(\mathbb {K}_j). \end{aligned}$$

Let us now turn to the aforementioned subclass of $\Psi ^r(E,F)$ for integral *r*.

### Definition 1

Suppose $r\in \mathbb {Z}$. A principal cosymbol $a\in \mathcal {K}({\mathcal {T}}M;E,F)$ is said to be *essentially projectively homogeneous of order r* if $(\delta _\lambda )_*a=\lambda ^ra$ mod $\mathcal {K}^\infty ({\mathcal {T}}M;E,F)$ holds for all $\lambda \ne 0$. Correspondingly, we put

$$\begin{aligned} \Sigma ^r_2(E,F):=\left\{ a\in \frac{\mathcal {K}({\mathcal {T}}M;E,F)}{\mathcal {K}^\infty ({\mathcal {T}}M;E,F)}:(\delta _\lambda )_*a=\lambda ^ra\, \hbox {for all }\lambda \ne 0\right\} , \end{aligned}$$

using the subscript 2 to indicate this additional $\mathbb {Z}_2$ symmetry. Note that

25 $$\begin{aligned} \Sigma ^r_2(E,F)=\bigl \{a\in \Sigma ^r(E,F):(\delta _{-1})_*a=(-1)^ra\bigr \}. \end{aligned}$$

Let us also introduce the notation

26 $$\begin{aligned} \mathcal {P}^r({\mathcal {T}}M;E,F):=\bigl \{a\in \mathcal {K}^\infty ({\mathcal {T}}M;E,F):(\delta _\lambda )_*a=\lambda ^ra\, \hbox {for all }\lambda >0\bigr \}. \end{aligned}$$

Clearly, $\mathcal {P}^r({\mathcal {T}}M;E,F)=0$ if $-r-n\notin \mathbb {N}_0$. For $-r-n\in \mathbb {N}_0$ these are smooth kernels which are polynomial along each fiber $\mathcal {T}_xM$. In particular, the homogeneity in (26) remains true for all $\lambda \ne 0$. As in [21, Lemma 3.8] one can show:

### Lemma 2

Suppose $a\in \mathcal {K}({\mathcal {T}}M;E,F)$ and $r\in \mathbb {Z}$. Then *a* is essentially projectively homogeneous of order *r*, i.e., represents an element in $\Sigma ^r_2(E,F)$, if and only if there exist $a_\infty \in \mathcal {K}^\infty ({\mathcal {T}}M;E,F)$, $q\in \mathcal {K}({\mathcal {T}}M;E,F)$ and $p\in \mathcal {P}^r({\mathcal {T}}M;E,F)$ such that $a=a_\infty +q$ and

$$\begin{aligned} (\delta _\lambda )_*q=\lambda ^rq+\lambda ^r\log |\lambda |p,\qquad {\text {for all }}\lambda \ne 0. \end{aligned}$$

Here *q* is only unique mod $\mathcal {P}^r({\mathcal {T}}M;E,F)$, but *p* is without ambiguity.

### Definition 2

An operator $A\in \Psi ^r(E,F)$ of integral order $r\in \mathbb {Z}$ is called *projective* if its Schwartz kernel *k* admits an extension across the tangent groupoid, $\mathbb {K}\in \mathcal {K}(\mathbb {T}M;E,F)$, which is essentially projectively homogeneous of order *r*, that is, ${{\,\mathrm{ev}\,}}_1(\mathbb {K})=k$, and for all $\lambda \ne 0$ we have $(\delta ^{\mathbb {T}M}_\lambda )_*\mathbb {K}=\lambda ^r\mathbb {K}$ mod $\mathcal {K}^\infty (\mathbb {T}M;E,F)$. We will denote these operators by $\Psi ^r_2(E,F)$.

### Proposition 1

The class $\Psi ^r_2$ has the following properties:

- If $A\in \Psi ^r_2(E,F)$ and $B\in \Psi ^l_2(F,G)$ then $BA\in \Psi ^{l+r}_2(E,G)$, provided at least one of *A* and *B* is properly supported.
- If $$A\in \Psi ^r_2(E,F)$$, then $$A^t\in \Psi ^r_2(F',E')$$.
- If $A\in \Psi ^r_2(E,F)$, then $\sigma ^r(A)\in \Sigma ^r_2(E,F)$. Moreover,
  $$\begin{aligned} 0\rightarrow \Psi ^{r-1}_2(E,F)\rightarrow \Psi ^r_2(E,F)\xrightarrow {\sigma ^r}\Sigma ^r_2(E,F)\rightarrow 0 \end{aligned}$$
  is a natural short exact sequence.
- $\bigcap _{r\in \mathbb {Z}}\Psi ^r_2(E,F)=\mathcal {O}^{-\infty }(E,F)$, the smoothing operators.
- For $r\in \mathbb {N}_0$ we have $\mathcal {D}\mathcal {O}^r(E,F)=\mathcal {D}\mathcal {O}(E,F)\cap \Psi ^r_2(E,F)$.
- If an operator in $\Psi ^r_2(E,F)$ admits a left parametrix in $\Psi ^{-r}(F,E)$, then it also admits a left parametrix $\Psi ^{-r}_2(F,E)$. An analogous statement holds true for right parametrices.
- Suppose $A\in \Psi ^r(E,F)$ and let $\phi ^*(k|_V)\sim \sum _{j=0}^\infty k_j$ with $k_j\in \Sigma ^{r-j}(E,F)$ denote the asymptotic expansion of its kernel along the diagonal with respect to adapted exponential coordinates. Then $A\in \Psi ^r_2(E,F)$ if and only if $k_j\in \Sigma ^{r-j}_2(E,F)$ for all $j\in \mathbb {N}_0$.

### Proof

Parts (a) and (b) can be proved exactly as in [56], see also [21, Proposition 3.4]. Indeed, all maps in the commutative diagram (20) are multiplicative.

To see (c) put

$$\begin{aligned} \Sigma ^r_2(\mathbb {T}M;E,F):=\left\{ \mathbb {K}\in \frac{\mathcal {K}(\mathbb {T}M;E,F)}{\mathcal {K}^\infty (\mathbb {T}M;E,F)}:(\delta ^{\mathbb {T}M}_\lambda )_*\mathbb {K}=\lambda ^r\mathbb {K}\, \hbox {for all }\lambda \ne 0\right\} \end{aligned}$$

and note that

27 $$\begin{aligned} \Sigma ^r_2(\mathbb {T}M;E,F)=\bigl \{\mathbb {K}\in \Sigma ^r(\mathbb {T}M;E,F):(\delta ^{\mathbb {T}M}_{-1})_*\mathbb {K}=(-1)^r\mathbb {K}\bigr \}. \end{aligned}$$

Moreover, introduce

$$\begin{aligned} \Sigma ^r_2(\mathcal {T}^\text {op}M;E,F):=\left\{ a\in \frac{\mathcal {K}(\mathcal {T}^\text {op}M;E,F)}{\mathcal {K}^\infty (\mathcal {T}^\text {op}M;E,F)}:(\delta ^\text {op}_\lambda )_*a=\lambda ^ra\hbox { for all } \lambda \ne 0\right\} , \end{aligned}$$

and note that

28 $$\begin{aligned} \Sigma ^r_2(\mathcal {T}^\text {op}M;E,F)=\bigl \{a\in \Sigma ^r(\mathcal {T}^\text {op}M;E,F):(\delta ^\text {op}_{-1})_*a=(-1)^ra\bigr \}. \end{aligned}$$

Using the commutativity of the left square in (20) and (22) we see that (21) restricts to a commutative diagram:29Using (28), (27), and the averaging operator $\frac{1}{2}\bigl ({{\,\mathrm{id}\,}}+(-1)^{-r}(\delta ^{\mathbb {T}M}_{-1})_*\bigr )$, one readily sees that the bottom row in the diagram above is exact, for the same is true in (21). Consequently, the top row in (29) is also exact, whence (c).

Part (d) follows immediately from $\bigcap _{r\in \mathbb {Z}}\Psi ^r(E,F)=\mathcal {O}^{-\infty }(E,F)$, see [56, Corollary 53] or [21, Proposition 3.4(c)], for we have the obvious inclusions $\mathcal {O}^{-\infty }(E,F)\subseteq \Psi ^r_2(E,F)\subseteq \Psi ^r(E,F)$.

The proof of [21, Proposition 3.4(f)] actually shows (e), see also [56, Sect. 10.3].

To see (f), consider $A\in \Psi ^r_2(E,F)$ and $B\in \Psi ^{-r}(F,E)$ such that $BA-{{\,\mathrm{id}\,}}$ is a smoothing operator. Choose $\mathbb {K}\in \Sigma ^r_2(\mathbb {T}M;E,F)$ such that ${{\,\mathrm{ev}\,}}_1(\mathbb {K})$ represents *A* mod smoothing operators. Moreover, choose $\mathbb {L}\in \Sigma ^{-r}(\mathbb {T}M;F,E)$ such that ${{\,\mathrm{ev}\,}}_1(\mathbb {L})$ represents *B* mod smoothing operators. Since ${{\,\mathrm{ev}\,}}_1$ induces a multiplicative isomorphism as indicated in (21), we conclude $\mathbb {L}\mathbb {K}=1$ in $\Sigma ^0(\mathbb {T}M;E,E)$. Consider $\tilde{\mathbb {L}}:=\frac{1}{2}\bigl (\mathbb {L}+(-1)^r(\delta ^{\mathbb {T}M}_{-1})_*\mathbb {L}\bigr )\in \Sigma ^{-r}_2(\mathbb {T}M;\mathbb {F},\mathbb {E})$. In view of $1=(\delta ^{\mathbb {T}M}_{-1})_*1=(\delta ^{\mathbb {T}M}_{-1})_*(\mathbb {L}\mathbb {K})=\bigl ((\delta ^{\mathbb {T}M}_{-1})_*\mathbb {L}\bigr )\bigl ((\delta ^{\mathbb {T}M}_{-1})_*\mathbb {K}\bigr )=\bigl ((\delta ^{\mathbb {T}M}_{-1})_*\mathbb {L}\bigr )\bigl ((-1)^r\mathbb {K}\bigr )=\bigl ((-1)^r(\delta ^{\mathbb {T}M}_{-1})_*\mathbb {L}\bigr )\mathbb {K}$, we conclude $\tilde{\mathbb {L}}\mathbb {K}=1$ in $\Sigma ^0_2(\mathbb {T}M;E,E)$. Hence, ${{\,\mathrm{ev}\,}}_1(\tilde{\mathbb {L}})$ gives rise to a left parametrix ${\tilde{B}}\in \Psi ^{-r}_2(F,E)$ such that ${\tilde{B}}A-{{\,\mathrm{id}\,}}$ is a smoothing operator.

To see (g), consider $\mathbb {K}\in \Sigma ^r(\mathbb {T}M;E,F)$ and suppose the kernel $k={{\,\mathrm{ev}\,}}_1(\mathbb {K})$ represents the operator $A\in \Psi ^r(E,F)$. From (23) we get

30 $$\begin{aligned} \Phi ^*\bigl ((\delta _{-1}^{\mathbb {T}M})_*\mathbb {K}|_{\mathbb {V}}\bigr )\sim \sum _{j=0}^\infty (-1)^j(\delta ^\text {op}_{-1})_*(\mathbb {K}_j)t^j, \end{aligned}$$

since we have $\delta _\lambda ^{\mathbb {T}M}(\Phi (g,t))=\Phi \bigl (\delta ^\text {op}_\lambda (g),t/\lambda \bigr )$ for all $\lambda \ne 0$. Now, $A\in \Psi ^r_2(E,F)$ iff $(\delta _{-1}^{\mathbb {T}M})_*\mathbb {K}=(-1)^r\mathbb {K}$ in $\Sigma ^r(\mathbb {T}M;E,F)$, see (27). Comparing (23) with (30), we conclude that this is the case iff $(\delta ^\text {op}_{-1})_*\mathbb {K}_j=(-1)^{r-j}\mathbb {K}_j$ in $\Sigma ^{r-j}(\mathcal {T}^\text {op}M;E,F)$, for all *j*. Using (28), we see that this holds iff $\mathbb {K}_j\in \Sigma ^{r-j}_2(\mathcal {T}^\text {op}M;E,F)$, for all *j*. In view of (24), this is in turn equivalent to $k_j\in \Sigma ^{r-j}_2(E,F)$, for all *j*. $\square$

### Remark 1

Proposition 1(b) implies that the class $\Psi ^r_2$ is invariant under taking formal adjoints too. More precisely, if $A\in \Psi ^r_2(E,F)$, then $A^*\in \Psi ^r_2(F,E)$ where the formal adjoint is with respect to inner products of the form (4).

## Heat Kernel Asymptotics

The aim of this section is to prove Theorem 1. To this end let *E* be a vector bundle over a closed filtered manifold *M*, and suppose $A\in \mathcal {D}\mathcal {O}^r(E)$ is a differential Rockland operator of even Heisenberg order $r>0$ which is formally self-adjoint and non-negative with respect to the $L^2$ inner product induced by a volume density $\mathrm{d}x$ on *M* and a fiberwise Hermitian metric *h* on *E*, see (4).

We turn $M\times \mathbb {R}$ into a filtered manifold by putting

$$\begin{aligned} T^p(M\times \mathbb {R}):={\left\{ \begin{array}{ll}\pi _1^*T^pM&{}\text {if }p>-r, \text {and}\\ \pi _1^*T^pM\oplus \pi _2^*T\mathbb {R}&{}\text {if }p\le -r.\end{array}\right. } \end{aligned}$$

Here $\pi _1:M\times \mathbb {R}\rightarrow M$ and $\pi _2:M\times \mathbb {R}\rightarrow \mathbb {R}$ denote the canonical projections, and we identify $T(M\times \mathbb {R})=\pi _1^*TM\oplus \pi _2^*T\mathbb {R}$. We will regard *A* and $\frac{\partial }{\partial t}$ as operators over $M\times \mathbb {R}$ acting on sections of the pull back bundle ${\tilde{E}}:=\pi _1^*E$. The fiberwise Hermitian metric *h* on *E* induces a fiberwise Hermitian metric ${\tilde{h}}:=\pi _1^*h$ on ${\tilde{E}}$. Furthermore, the volume density $\mathrm{d}x$ on *M* and the standard volume density $\mathrm{d}t$ on $\mathbb {R}$ provide a volume density $\mathrm{d}x\mathrm{d}t$ on $M\times \mathbb {R}$. We will use the $L^2$ inner product on sections of ${\tilde{E}}$ induced by $\mathrm{d}x\mathrm{d}t$ and ${\tilde{h}}$, see (4).

This filtration on $M\times \mathbb {R}$ is motivated by the fact that the heat operator $A+\frac{\partial }{\partial t}$ becomes a Rockland operator. More precisely, we have:

### Lemma 3

The differential operator $A+\frac{\partial }{\partial t}$ is a Rockland operator of Heisenberg order *r*. The same is true for $(A+\frac{\partial }{\partial t})^*=A-\frac{\partial }{\partial t}$.

### Proof

Clearly, $\frac{\partial }{\partial t}\in \Gamma ^\infty (T^{-r}(M\times \mathbb {R}))$, hence $\frac{\partial }{\partial t}$ is a differential operator of Heisenberg order at most *r* on $M\times \mathbb {R}$. Since $\pi _1^*T^pM\subseteq T^p(M\times \mathbb {R})$, the operator *A* has Heisenberg order at most *r* when considered on $M\times \mathbb {R}$. Consequently, $A+\frac{\partial }{\partial t}$ has Heisenberg order at most *r*.

For $(x,t)\in M\times \mathbb {R}$, the canonical projections induce a canonical isomorphism of osculating algebras,

31 $$\begin{aligned} \mathfrak t_{(x,t)}(M\times \mathbb {R})=\mathfrak t_xM\oplus \mathbb {R}, \end{aligned}$$

where $\mathbb {R}$ is a central ideal in homogeneous degree $-r$. Correspondingly, we obtain a canonical isomorphism of osculating groups,

32 $$\begin{aligned} \mathcal {T}_{(x,t)}(M\times \mathbb {R})=\mathcal {T}_xM\times \mathbb {R}, \end{aligned}$$

such that the parabolic dilation becomes

33 $$\begin{aligned} \delta ^{\mathcal {T}(M\times \mathbb {R})}_{\lambda ,(x,t)}(g,\tau )=\bigl (\delta ^{{\mathcal {T}}M}_{\lambda ,x}(g),\lambda ^r\tau \bigr ), \end{aligned}$$

where $$g\in \mathcal {T}_xM$$, $$\tau \in \mathbb {R}$$, and $$\lambda \ne 0$$.

Let $\sigma ^r_x(A)\in \mathcal {U}_{-r}(\mathfrak t_xM)\otimes {{\,\mathrm{end}\,}}(E_x)$ denote the Heisenberg principal symbol of *A*. For the Heisenberg principal symbol of $A+\tfrac{\partial }{\partial t}$ we clearly have

34 $$\begin{aligned} \sigma ^r_{(x,t)}\left( A+\tfrac{\partial }{\partial t}\right) =\sigma ^r_x(A)+T, \end{aligned}$$

via the canonical isomorphism induced by (31),

$$\begin{aligned} \mathcal {U}_{-r}\bigl (\mathfrak t_{(x,t)}(M\times \mathbb {R})\bigr )\otimes {{\,\mathrm{end}\,}}\bigl ({\tilde{E}}_{(x,t)}\bigr ) =\bigl (\mathcal {U}(\mathfrak t_xM)\otimes \mathbb {R}[T]\bigr )_{-r}\otimes {{\,\mathrm{end}\,}}(E_x). \end{aligned}$$

Here $\mathbb {R}[T]=\mathcal {U}(\mathbb {R})$ denotes the polynomial algebra in one variable *T* of homogeneous degree $-r$.

To verify the Rockland condition, consider a non-trivial irreducible unitary representation of the osculating group, $\pi :\mathcal {T}_{(x,t)}(M\times \mathbb {R})\rightarrow U(\mathcal {H})$, on a Hilbert space $\mathcal {H}$. Since the factor $\mathbb {R}$ in (31) is central, $b:=\pi (T)$ acts by a scalar on $\mathcal {H}$, see [38, Theorem 5 in Appendix V]. Hence, restricting $\pi$ via (32), we obtain an irreducible representation $\bar{\pi }:\mathcal {T}_xM\rightarrow U(\mathcal {H})$. Putting $a:=\bar{\pi }(\sigma ^r_x(A))$, (34) gives

35 $$\begin{aligned} \pi \left( \sigma ^r_x\left( A+\tfrac{\partial }{\partial t}\right) \right) =a+b. \end{aligned}$$

Let $\mathcal {H}_\infty$ denote the space of smooth vectors for the representation $\pi$, and note that this coincides with the space of smooth vectors of $\bar{\pi }$. By unitarity, and since *A* is assumed to be formally self-adjoint, we have $a^*=a$ and $b^*=-b$, see (5), hence

$$\begin{aligned} (a-b)(a+b)=a^*a+b^*b. \end{aligned}$$

By positivity, it thus suffices to show that *a* or *b* acts injectively on $\mathcal {H}_\infty$, see (35). If $\bar{\pi }$ is non-trivial, then *a* acts injectively for *A* is assumed to satisfy the Rockland condition. If $\bar{\pi }$ is trivial, then *b* acts injectively, for it has to be a non-trivial scalar.^3^    $\square$

In view of [21, Theorem 3.13] and Lemma 3, the operator $A+\frac{\partial }{\partial t}$ admits a properly supported parametrix ${\tilde{Q}}\in \Psi _\text {prop}^{-r}({\tilde{E}})$, see also [21, Remarks 3.18 and 3.19]. Hence, there exist smoothing operators $R_1$ and $R_2$ over $M\times \mathbb {R}$ such that

36 $$\begin{aligned} {\tilde{Q}}\left( A+\tfrac{\partial }{\partial t}\right) ={{\,\mathrm{id}\,}}+R_1 \qquad \text {and}\qquad \left( A+\tfrac{\partial }{\partial t}\right) {\tilde{Q}}={{\,\mathrm{id}\,}}+R_2. \end{aligned}$$

By the definition of the class $\Psi ^{-r}_\text {prop}({\tilde{E}})$, see Sect. 3 or [21, Definition 3.2], ${\tilde{Q}}$ has a properly supported Schwartz kernel, $k_{{\tilde{Q}}}\in \Gamma _\text {prop}^{-\infty }({\tilde{E}}\boxtimes {\tilde{E}}')$, whose wave front set is contained in the conormal of the diagonal. In particular, $k_{{\tilde{Q}}}$ is smooth away from the diagonal. Moreover, $k_{{\tilde{Q}}}$ admits an asymptotic expansion along the diagonal.

To describe the asymptotic expansion of the kernel $k_{{\tilde{Q}}}$, we will use adapted exponential coordinates for $M\times \mathbb {R}$ which are compatible with the product structure and the translational invariance of the operator $A+\frac{\partial }{\partial t}$. We start with adapted exponential coordinates for *M* as described in Sect. 3 and summarized in the commutative diagram (16). The exponential coordinates on $M\times \mathbb {R}$ will be defined such that the following diagram commutes:Here $\tilde{\pi }:\mathcal {T}(M\times \mathbb {R})\rightarrow M\times \mathbb {R}$ denotes the bundle projection, ${\tilde{o}}$ is the zero section, and $\tilde{\Delta }$ denotes the diagonal mapping. Moreover,

37 $$\begin{aligned} {\tilde{V}}&:=\{(x,s;y,t)\in (M\times \mathbb {R})\times (M\times \mathbb {R}):(x,y)\in V\}\nonumber \\ {\tilde{U}}_{(x,s)}&:=\{(g,t)\in \mathcal {T}_{(x,s)}(M\times \mathbb {R})=\mathcal {T}_xM\times \mathbb {R}:g\in U\}\nonumber \\ \tilde{\varphi }_{(x,s)}(g,t)&:=(x,s;\varphi _x(g),s-t) \end{aligned}$$

38 $$\begin{aligned} \tilde{\phi }_{(x,s)}(g,t)&:=\phi _x(g) \end{aligned}$$

where $(x,s)\in M\times \mathbb {R}$ and $(g,t)\in \mathcal {T}_{(x,s)}(M\times \mathbb {R})=\mathcal {T}_xM\times \mathbb {R}$. Note that these are exponential coordinates as described in Sect. 3 associated with the splitting of the filtration $T(M\times \mathbb {R})\cong \mathfrak t(M\times \mathbb {R})$ induced by the splitting $TM\cong \mathfrak tM$ and the linear connection on $T(M\times \mathbb {R})=\pi _1^*TM\oplus \pi _2^*T\mathbb {R}$ induced from the linear connection on *TM* and the trivial connection on $T\mathbb {R}$.

Pulling back the Schwartz kernel of ${\tilde{Q}}$ with $\tilde{\phi }$, we obtain a distributional section $\tilde{\phi }^*(k_{{\tilde{Q}}}|_{{\tilde{V}}})$ of the vector bundle ${{\,\mathrm{end}\,}}(\tilde{\pi }^*{\tilde{E}})\otimes \Omega _{\tilde{\pi }}$ over ${\tilde{U}}$. According to Proposition 1(e) and (f), the parametrix ${\tilde{Q}}$ may be assumed to be in the class $\Psi ^{-r}_2({\tilde{E}})$. Hence, see Proposition 1(g), we have an asymptotic expansion of the form

39 $$\begin{aligned} \tilde{\phi }^*\bigl (k_{{\tilde{Q}}}|_{{\tilde{V}}}\bigr )\sim \sum _{j=0}^\infty {\tilde{q}}_j \end{aligned}$$

where ${\tilde{q}}_j\in \Sigma ^{-r-j}_2({\tilde{E}})$. More precisely, for every integer *N* there exists an integer $j_N$ such that $\tilde{\phi }^*(k_{{\tilde{Q}}}|_{{\tilde{V}}})-\sum _{j=0}^{j_N}{\tilde{q}}_j$ is of class $C^N$ on ${\tilde{U}}$. According to Lemma 2, we may fix representatives ${\tilde{q}}_j\in \mathcal {K}\bigl (\mathcal {T}(M\times \mathbb {R});{\tilde{E}}\bigr )$ satisfying

40 $$\begin{aligned} \bigl (\delta ^{\mathcal {T}(M\times \mathbb {R})}_\lambda \bigr )_*{\tilde{q}}_j=\lambda ^{-r-j}{\tilde{q}}_j-\lambda ^{-r-j}\log |\lambda |\,{\tilde{p}}_j,\qquad \lambda \ne 0, \end{aligned}$$

where ${\tilde{p}}_j\in \mathcal {P}^{-r-j}\bigl (\mathcal {T}(M\times \mathbb {R});{\tilde{E}}\bigr )$ is smooth and strictly homogeneous,

$$\begin{aligned} \bigl (\delta ^{\mathcal {T}(M\times \mathbb {R})}_\lambda \bigr )_*{\tilde{p}}_j=\lambda ^{-r-j}{\tilde{p}}_j,\qquad \lambda \ne 0. \end{aligned}$$

The representative ${\tilde{q}}_j$ satisfying (40) is unique mod $\mathcal {P}^{-r-j}\bigl (\mathcal {T}(M\times \mathbb {R});{\tilde{E}}\bigr )$. The logarithmic terms ${\tilde{p}}_j$, however, are without ambiguity.

The heat semi-group $e^{-tA}$ permits to invert the heat operator $A+\frac{\partial }{\partial t}$ over $M\times \mathbb {R}$. Following [4, Sect. 5] we let $C_+(\mathbb {R},L^2(E))$ denote the space of continuous functions $\psi :\mathbb {R}\rightarrow L^2(E)$ for which there exists $t_0\in \mathbb {R}$ such that $\psi (t)=0$ for all $t\le t_0$. We consider the operator $Q:C_+(\mathbb {R},L^2(E))\rightarrow C_+(\mathbb {R},L^2(E))$ defined by

41 $$\begin{aligned} (Q\psi )(s):=\int _{-\infty }^se^{-(s-t)A}\psi (t)\,\mathrm{d}t,\qquad \psi \in C_+(\mathbb {R},L^2(E)). \end{aligned}$$

Since $e^{-tA}$ is strongly continuous, the integrand $e^{-(s-t)A}\psi (t)$ depends continuously on $t\in (-\infty ,s]$, hence the integral converges and $Q\psi \in C_+(\mathbb {R},L^2(E))$, cf. [37, Lemma 3.7 in Sect. III§3.1]. Since $e^{-tA}$ is a contraction, the estimate $\Vert (Q\psi )(s)\Vert \le |s-t_0|\sup _{t\in [t_0,s]}\Vert \psi (t)\Vert$ holds for all $\psi \in C_+(\mathbb {R},L^2(E))$ which are supported on $[t_0,\infty )$. This shows that *Q* is continuous, when $C_+(\mathbb {R},L^2(E))=\varinjlim C_{[t_0,\infty )}(\mathbb {R},L^2(E))$ is equipped with the inductive limit topology over $t_0\in \mathbb {R}$, and $C_{[t_0,\infty )}(\mathbb {R},L^2(E))$ denotes the subspace of continuous maps which are supported on $[t_0,\infty )$ carrying the topology of uniform convergence on compact subsets. Composing *Q* with the continuous inclusions $\Gamma _c^\infty ({\tilde{E}})\subseteq C_+(\mathbb {R},L^2(E))$ and $C_+(\mathbb {R},L^2(E))\subseteq \Gamma ^{-\infty }({\tilde{E}})$, we may thus regard it as a continuous operator, $Q:\Gamma ^\infty _c({\tilde{E}})\rightarrow \Gamma ^{-\infty }({\tilde{E}})$. By the Schwartz kernel theorem, *Q* has a distributional kernel, $k_Q\in \Gamma ^{-\infty }({\tilde{E}}\boxtimes {\tilde{E}}')$, that is,

42 $$\begin{aligned} \langle \phi ,Q\psi \rangle =\int _{(x,s;y,t)\in (M\times \mathbb {R})\times (M\times \mathbb {R})}\phi (x,s)k_Q(x,s;y,t)\psi (y,t) \end{aligned}$$

for $\psi \in \Gamma ^\infty _c({\tilde{E}})$ and $\phi \in \mathcal {D}({\tilde{E}})=\Gamma ^\infty _c({\tilde{E}}')$. From (6) and (41) we get

43 $$\begin{aligned} \left( A+\tfrac{\partial }{\partial t}\right) Q\psi =\psi =Q\left( A+\tfrac{\partial }{\partial t}\right) \psi \end{aligned}$$

in the distributional sense, for all $\psi \in \Gamma ^\infty _c({\tilde{E}})$, cf. [4, Eq. (5.12)].

Comparing (43) with (36) we obtain, as in [4, Sect. 5],

44 $$\begin{aligned} ({{\,\mathrm{id}\,}}+R_1)Q\psi ={\tilde{Q}}\psi =Q({{\,\mathrm{id}\,}}+R_2)\psi \end{aligned}$$

for all $\psi \in \Gamma ^\infty _c({\tilde{E}})$. Note here, that ${\tilde{Q}}$ is properly supported, defining continuous operators on $\Gamma ^{-\infty }({\tilde{E}})$ and $\Gamma ^\infty _c({\tilde{E}})$. Moreover, $R_1$ and $R_2$ are properly supported smoothing operators, defining continuous operators $\Gamma ^{-\infty }_c({\tilde{E}})\rightarrow \Gamma ^\infty _c({\tilde{E}})$ and $\Gamma ^{-\infty }({\tilde{E}})\rightarrow \Gamma ^\infty ({\tilde{E}})$. The equality on the left-hand side in (44) implies that *Q* maps $\Gamma ^\infty _c({\tilde{E}})$ continuously into $\Gamma ^\infty ({\tilde{E}})$. Hence, $QR_2$ is a smoothing operator, for it maps $\Gamma ^{-\infty }_c({\tilde{E}})$ continuously into $\Gamma ^\infty ({\tilde{E}})$. Using the equality on the right-hand side of (44), we conclude that ${\tilde{Q}}-Q$ is a smoothing operator.

Since *Q* differs from ${\tilde{Q}}$ by a smoothing operator, $k_Q$ is smooth away from the diagonal and (39) gives an asymptotic expansion

45 $$\begin{aligned} \tilde{\phi }^*\bigl (k_Q|_{{\tilde{V}}}\bigr )\sim \sum _{j=0}^\infty {\tilde{q}}_j. \end{aligned}$$

Using Taylor’s theorem, we may, by adding terms in $\mathcal {P}^{-r-j}\bigl (\mathcal {T}(M\times \mathbb {R});{\tilde{E}}\bigr )$ to ${\tilde{q}}_j$, assume that for every integer *N* there exists an integer $j_N$ such that

46 $$\begin{aligned} \left( \tilde{\phi }^*\bigl (k_Q|_{{\tilde{V}}}\bigr )-\sum _{j=0}^{j_N}{\tilde{q}}_j\right) _{(x,s)}({\tilde{g}})=O\bigl (|{\tilde{g}}|^N\bigr ), \end{aligned}$$

as ${\tilde{g}}\rightarrow {\tilde{o}}_{(x,s)}\in \mathcal {T}_{(x,s)}(M\times \mathbb {R})$, uniformly for (*x*, *s*) in compact subsets of $M\times \mathbb {R}$. Here $|-|$ denotes a fiberwise homogeneous norm on $\mathcal {T}(M\times \mathbb {R})$. Comparing (7), (41), and (42), we find

47 $$\begin{aligned} k_Q(x,s;y,s-t)={\left\{ \begin{array}{ll}k_t(x,y)\mathrm{d}t&{}\text {for t>0, and}\\ 0&{}\text {for t<0.}\end{array}\right. } \end{aligned}$$

In particular, $k_t(x,y)$ is smooth on $M\times M\times (0,\infty )$. Furthermore, $\tilde{\phi }^*_{(x,s)}(k_Q|_{{\tilde{V}}})$ vanishes on $\mathcal {T}_xM\times (-\infty ,0)\subseteq \mathcal {T}_xM\times \mathbb {R}=\mathcal {T}_{(x,s)}(M\times \mathbb {R})$ and does not depend on *s*, see (37) and (38). Combining this with (46) and (40), we conclude that

48 $$\begin{aligned} {\tilde{q}}_{j,x}:={\tilde{q}}_{j,(x,s)}\text { vanishes on } \mathcal {T}_xM\times (-\infty ,0)\subseteq \mathcal {T}_{(x,s)}(M\times \mathbb {R}) \end{aligned}$$

and does not depend on $s\in \mathbb {R}$, for every $j\in \mathbb {N}_0$ and all $x\in M$. Note here that $\mathcal {T}_xM\times (-\infty ,0)$ is invariant under scaling, see (33). Using (48) and (40), we see that ${\tilde{p}}_{j,(x,s)}$ vanishes on $\mathcal {T}_xM\times (-\infty ,0)$. Since ${\tilde{p}}_{j,(x,s)}$ is polynomial, we conclude ${\tilde{p}}_{j,(x,s)}=0$ for every $j\in \mathbb {N}_0$ and all $(x,s)\in M\times \mathbb {R}$. This shows that there are no log terms in the asymptotic expansion (45). In other words,

49 $$\begin{aligned} \bigl (\delta ^{\mathcal {T}(M\times \mathbb {R})}_\lambda \bigr )_*{\tilde{q}}_j=\lambda ^{-r-j}{\tilde{q}}_j,\qquad \lambda \ne 0. \end{aligned}$$

Restricting (46) we obtain, using (37) and (33),

50 $$\begin{aligned} k_Q(x,s;x,s-t)=\sum _{j=0}^{j_N}\tilde{\phi }_{(x,s)}\bigl ({\tilde{q}}_{j,(x,s)}(o_x,t)\bigr )+O\bigl (|t|^{N/r}\bigr ), \end{aligned}$$

as $|t|\rightarrow 0$, uniformly in *x*. Using (49), (38), (33), and (2), where *n* has to be replaced by the homogeneous dimension $n+r$ of $M\times \mathbb {R}$, we obtain

51 $$\begin{aligned} \tilde{\phi }_{(x,s)}\bigl ({\tilde{q}}_{j,(x,s)}(o_x,t)\bigr )=t^{(j-n)/r}\tilde{\phi }_{(x,s)}\bigl ({\tilde{q}}_{j,(x,s)}(o_x,1)\bigr ) \end{aligned}$$

for all $x\in M$, $s\in \mathbb {R}$, and $t>0$. In Eq. (51) we are using (left) trivialization of the 1-density bundle of the osculating group $\mathcal {T}_{(x,s)}(M\times \mathbb {R})$ to identify the two sides. Hence, defining $q_j\in \Gamma ^\infty \bigl ({{\,\mathrm{end}\,}}(E)\otimes |\Lambda _M|\bigr )$ by

52 $$\begin{aligned} q_j(x)\mathrm{d}t:=\tilde{\phi }_{(x,s)}\bigl ({\tilde{q}}_{j,(x,s)}(o_x,1)\bigr ), \end{aligned}$$

we obtain from (47), (50), (51), and (52)

$$\begin{aligned} k_t(x,x)=\sum _{j=0}^{N-1}t^{(j-n)/r}q_j(x)+O\bigl (t^{(N-n)/r}\bigr ), \end{aligned}$$

as $t\searrow 0$, uniformly in *x*.

Using $\lambda =-1$ in (49) we see that ${\tilde{q}}_{j,(x,s)}(o_x,t)=0$ for all odd *j* and $t\ne 0$, see (33) and (2). Hence, $q_j(x)=0$ for all odd *j*, see (52).

### Remark 2

The asymptotic term $q_j\in \Gamma ^\infty \bigl ({{\,\mathrm{end}\,}}(E)\otimes |\Lambda _M|\bigr )$ in Theorem 1 can be read off the homogeneous term ${\tilde{q}}_j\in \Sigma ^{-r-j}({\tilde{E}})$ in the asymptotic expansion (39) of any parametrix ${\tilde{Q}}$ for the heat operator $A+\frac{\partial }{\partial t}$ on $M\times \mathbb {R}$. Indeed, the representative ${\tilde{q}}_j\in \mathcal {K}(\mathcal {T}(M\times \mathbb {R});{\tilde{E}})$ used in (52) is uniquely characterized by (51) and (48). Note that these representatives are also translation invariant, corresponding precisely to the homogeneous terms on which the Volterra–Heisenberg calculus is based on, see [4, Sect. 3] and [43, Sect. 5.1].

To complete the proof of Theorem 1 it remains to show $q_0(x)>0$. This will be accomplished using the subsequent lemma concerning the heat kernel on the osculating groups, cf. [43, Lemma 6.1.4].

### Lemma 4

Consider the Heisenberg principal symbol $\sigma _x^r(A)$ as a left invariant homogeneous differential operator acting on $C^\infty (\mathcal {T}_xM,E_x)$. The corresponding heat equation admits a kernel (fundamental solution) in the Schwartz space of ${{\,\mathrm{end}\,}}(E_x)$-valued 1-densities on $\mathcal {T}_xM$. More precisely, there exists a family

53 $$\begin{aligned} k_t^{\sigma ^r_x(A)}\in \mathcal {S}\bigl (|\Lambda _{\mathcal {T}_xM}|\bigr )\otimes {{\,\mathrm{end}\,}}(E_x), \end{aligned}$$

depending smoothly on $t>0$, which satisfies the heat equation, i.e.,

54 $$\begin{aligned} \tfrac{\partial }{\partial t}k_t^{\sigma _x^r(A)}=-\sigma _x^r(A)k_t^{\sigma _x^r(A)}, \end{aligned}$$

and the initial condition

55 $$\begin{aligned} \lim _{t\searrow 0}k_t^{\sigma _x^r(A)}\psi =\psi , \end{aligned}$$

for all $\psi \in \mathcal {S}(\mathcal {T}_xM)\otimes E_x$. Moreover, this kernel is homogeneous, that is,

56 $$\begin{aligned} (\delta _{\lambda ,x}^{\mathcal {T}_xM})_*k_t^{\sigma ^r_x(A)}=k_{\lambda ^rt}^{\sigma ^r_x(A)}, \end{aligned}$$

for all $t>0$ and $\lambda \ne 0$. Furthermore, $k_t^{\sigma ^r_x(A)}(o_x)>0$ in $|\Lambda _{\mathcal {T}_xM,o_x}|\otimes {{\,\mathrm{end}\,}}(E_x)$, for each $t>0$. The heat kernel $k_t^{\sigma ^r_x(A)}$ is uniquely characterized by (54) in the following sense: Every family of tempered distributions $k_t''\in \mathcal {S}'(\mathcal {T}_xM)\otimes {{\,\mathrm{end}\,}}(E_x)$ which is continuously differentiable in $t>0$, satisfies the heat equation (54) and the initial condition $\lim _{t\searrow 0}k_t''=\delta _{o_x}$ in $\mathcal {S}'(\mathcal {T}_xM)\otimes {{\,\mathrm{end}\,}}(E_x)$, coincides with $k^{\sigma ^r_x(A)}_t$.

### Proof

Since ${\tilde{Q}}$ is a parametrix for $A+\frac{\partial }{\partial t}$, we have $\sigma ^r_{(x,s)}(A+\tfrac{\partial }{\partial t})\sigma ^{-r}_{(x,s)}({\tilde{Q}})=1$ in $\Sigma ^0_{(x,s)}({\tilde{E}})$, for any fixed $s\in \mathbb {R}$. As $\sigma ^r_{(x,s)}(A+\tfrac{\partial }{\partial t})=\sigma ^r_x(A)+\tfrac{\partial }{\partial t}$ and ${\tilde{q}}_{0,x}={\tilde{q}}_{0,(x,s)}=\sigma ^{-r}_{(x,s)}({\tilde{Q}})$, we get $\bigl (\sigma ^r_{x}(A)+\tfrac{\partial }{\partial t}\bigr ){\tilde{q}}_{0,x}=\delta _{(o_x,0)}$ mod $\mathcal {K}^\infty \bigl (\mathcal {T}_xM\times \mathbb {R};{\tilde{E}}_x\bigr )$. By homogeneity, see (49), we actually have

57 $$\begin{aligned} \bigl (\sigma ^r_{x}(A)+\tfrac{\partial }{\partial t}\bigr ){\tilde{q}}_{0,x}=\delta _{(o_x,0)}. \end{aligned}$$

Hence, restricting ${\tilde{q}}_{0,x}$ to $\mathcal {T}_xM\times (0,\infty )\subseteq \mathcal {T}_xM\times \mathbb {R}=\mathcal {T}_{(x,s)}(M\times \mathbb {R})$, more precisely, defining

58 $$\begin{aligned} k^{\sigma ^r_x(A)}_t\mathrm{d}t:={\tilde{q}}_{0,x}|_{\mathcal {T}_xM\times \{t\}},\qquad t>0, \end{aligned}$$

we obtain a smooth kernel satisfying the differential equation in (54). As ${\tilde{Q}}$ is also a left parametrix for $A+\frac{\partial }{\partial t}$, a similar argument shows

59 $$\begin{aligned} \tfrac{\partial }{\partial t}k_t^{\sigma _x^r(A)}=-k_t^{\sigma _x^r(A)}\sigma ^r_x(A), \end{aligned}$$

where the right-hand side involves the convolution of the (smooth) kernel $k_t^{\sigma _x^r(A)}$ with the (distributional) kernel of $\sigma ^r_x(A)$, a distribution on $\mathcal {T}_xM$ which is supported at the unit element $o_x$. The homogeneity formulated in (56) follows from (49), see also (33).

To show that $k_t^{\sigma ^r_x(A)}$ is in the Schwartz class, we fix a homogeneous norm $|-|$ and translation invariant volume density *dg* on $\mathcal {T}_xM$. Writing $k_t^{\sigma ^r_x(A)}=k_t'dg$ and ${\tilde{q}}_{0,x}=q'\mathrm{d}g\mathrm{d}t$, homogeneity implies

60 $$\begin{aligned} k_t'(g)=|g|^{-n}q'\bigl (\delta _{|g|}^{-1}(g),|g|^{-r}t\bigr ), \end{aligned}$$

for all $t>0$ and $o_x\ne g\in \mathcal {T}_xM$. Since $q'$ is smooth away from the origin in $\mathcal {T}_xM\times \mathbb {R}$ and vanishes on $\mathcal {T}_xM\times (-\infty ,0)$, see (48), it vanishes to infinite order along $\mathcal {T}_xM\times \{0\}$. Combining this with (60), we see that $|g|^mk_t'(g)$ tends to 0, as $|g|\rightarrow \infty$, for each $m\in \mathbb {N}$. The same argument shows that this remains true if $k'_t$ is replaced with $Bk_t'$ where *B* is some left invariant homogeneous differential operator on $\mathcal {T}_xM$. This shows that $k_t'$ is indeed in the Schwartz space $\mathcal {S}(\mathcal {T}_xM)\otimes {{\,\mathrm{end}\,}}(E_x)$, whence (53).

By homogeneity, see (56), the integral $\int _{\mathcal {T}_xM}k_t^{\sigma ^r_x(A)}$ is independent of *t*. Testing (57) with $\chi \in \Gamma ^\infty _c(\mathbb {R},E_x)$, considered as function on $\mathcal {T}_xM\times \mathbb {R}$ which is constant in $\mathcal {T}_xM$, we obtain

$$\begin{aligned} \chi (0) =\langle \delta _{(o_x,0)},\chi \rangle= & {} \left\langle \left( \sigma ^r_x(A)+\tfrac{\partial }{\partial t}\right) {\tilde{q}}_{0,x},\chi \right\rangle =-\langle {\tilde{q}}_{0,x},\chi '\rangle \\= & {} -\int _{\mathcal {T}_xM}k_t^{\sigma ^r_x(A)}\int _0^\infty \chi '(t)\mathrm{d}t =\Bigl (\int _{\mathcal {T}_xM}k_t^{\sigma ^r_x(A)}\Bigr )\chi (0), \end{aligned}$$

see (48) and (58). Consequently,

61 $$\begin{aligned} \int _{\mathcal {T}_xM}k_t^{\sigma ^r_x(A)}={{\,\mathrm{id}\,}}_{E_x}, \end{aligned}$$

for all $t>0$. Using (56), this readily implies the initial condition (55).

Let us now turn to the uniqueness of the heat kernel. The heat equation for $k_t''$ and (59) imply that for fixed $t>0$ the expression $k_s^{\sigma ^r_x(A)}k_{t-s}''$ is independent of $s\in (0,t)$. Moreover, the initial condition for $k_t''$ gives $\lim _{s\nearrow t}k_s^{\sigma ^r_x(A)}k_{t-s}''=k_t^{\sigma ^r_x(A)}$, while the initial condition (55) for $k_t^{\sigma ^r_x(A)}$ implies $\lim _{s\searrow 0}k_s^{\sigma ^r_x(A)}k_{t-s}''=k_t''$. Combining these observations, we obtain $k_t''=k_t^{\sigma ^r_x(A)}$, as well as the semi-group property

62 $$\begin{aligned} k^{\sigma ^r_x(A)}_sk^{\sigma ^r_x(A)}_t=k^{\sigma ^r_x(A)}_{s+t},\qquad s,t>0. \end{aligned}$$

Since *A* is formally self-adjoint, we also have $\bigl (k^{\sigma ^r_x(A)}_t\bigr )^*=k^{\sigma ^r_x(A)}_t$, that is,

63 $$\begin{aligned} k^{\sigma ^r_x(A)}_t(g^{-1})=\bigl (k^{\sigma ^r_x(A)}_t(g)\bigr )^*, \end{aligned}$$

for $g\in \mathcal {T}_xM$ and $t>0$. Combining (62) and (63), we obtain

64 $$\begin{aligned} k_{2t}^{\sigma ^r_x(A)}(o_x)=\int _{g\in \mathcal {T}_xM}\bigl (k^{\sigma ^r_x(A)}_t(g)\bigr )^*k^{\sigma ^r_x(A)}_t(g). \end{aligned}$$

Using (61), we conclude $k^{\sigma ^r_x(A)}_{2t}(o_x)>0$ in $|\Lambda _{\mathcal {T}_xM,o_x}|\otimes {{\,\mathrm{end}\,}}(E_x)$. $\square$

From (52) and (58) we get, up to the canonical identification $|\Lambda _{M,x}|=|\Lambda _{\mathcal {T}_xM,o_x}|$,

65 $$\begin{aligned} q_0(x)=k_1^{\sigma ^r_x(A)}(o_x). \end{aligned}$$

Hence, $q_0(x)>0$ in $|\Lambda _{M,x}|\otimes {{\,\mathrm{end}\,}}(E_x)$, for we have $k_t^{\sigma ^r_x(A)}(o_x)>0$ according to Lemma 4. This completes the proof of Theorem 1.

## Complex Powers

In this section, we will present a proof of Theorem 2 following the approach in [43, Sect. 5.3]. Throughout this section *E* denotes a vector bundle over a closed filtered manifold *M*, and $A\in \mathcal {D}\mathcal {O}^r(E)$ is a Rockland differential operator of even Heisenberg order $r>0$ which is formally self-adjoint and non-negative as in Theorem 1.

Recall that by hypoellipticity, $\ker (A)$ is a finite dimensional subspace of $\Gamma ^\infty (E)$, see [21, Corollary 2.10]. Let *P* denote the orthogonal projection onto $\ker (A)$. To express the complex powers using the Mellin formula,

66 $$\begin{aligned} A^{-z}=\frac{1}{\Gamma (z)}\int _0^\infty t^{z-1}(e^{-tA}-P)\mathrm{d}t,\qquad \mathfrak {R}(z)>0, \end{aligned}$$

we need the following standard estimate for the heat kernel for large time.

### Lemma 5

Let $p\in \Gamma ^\infty (E\boxtimes E')$ denote the Schwartz kernel of the orthogonal projection *P* onto $\ker (A)$. Then there exists $\varepsilon >0$ such that

67 $$\begin{aligned} k_t(x,y)=p(x,y)+O\bigl (e^{-t\varepsilon }\bigr ) \end{aligned}$$

as $t\rightarrow \infty$, uniformly with all derivatives in *x* and *y*.

### Proof

Since *A* is self-adjoint and non-negative, its spectrum is contained in $[0,\infty )$. Moreover, since *A* has compact resolvent, zero is isolated in its spectrum. Hence, by functional calculus, there exists $\varepsilon >0$ such that

$$\begin{aligned} e^{-tA}=P+O\bigl (e^{-t\varepsilon }\bigr ) \end{aligned}$$

as $t\rightarrow \infty$ with respect to the operator norm topology on $\mathcal {B}\bigl (L^2(E)\bigr )$. Writing

$$\begin{aligned} e^{-tA}=e^{-A/2}e^{-(t-1)A}e^{-A/2} \end{aligned}$$

and using the fact that $e^{-A/2}$ is a smoothing operator, we conclude

68 $$\begin{aligned} e^{-tA}=P+O\bigl (e^{-t\varepsilon }\bigr ) \end{aligned}$$

in $\mathcal {L}\bigl (\Gamma ^{-\infty }(E),\Gamma ^\infty (E)\bigr )$ with respect to the topology of uniform convergence on bounded subsets of $\Gamma ^{-\infty }(E)$, as $t\rightarrow \infty$. By nuclearity, and since $\Gamma ^{-\infty }(E)$ is the strong dual of $\Gamma ^\infty (E')$, we have canonical topological isomorphisms

$$\begin{aligned} \mathcal {L}\bigl (\Gamma ^{-\infty }(E),\Gamma ^\infty (E)\bigr ) =\Gamma ^\infty (E){\hat{\otimes }}\Gamma ^\infty (E')=\Gamma ^\infty (E\boxtimes E'), \end{aligned}$$

see [53, Eqs. (50.17) and (51.4)]. Hence, (68) is equivalent to (67). $\square$

Suppose $\mathfrak {R}(z)>0$. In view of the remarks on the spectrum at the beginning of the proof of Lemma 5, the integral on the right-hand side of the Mellin formula (66) converges to $A^{-z}$ with respect to the operator norm topology on $L^2(E)$. In particular, $A^{-z}$ is a bounded operator on $L^2(E)$ and its distributional kernel can be expressed in the form

$$\begin{aligned} k_{A^{-z}}(x,y)= \frac{1}{\Gamma (z)}\int _0^\infty t^{z-1}\bigl (k_t(x,y)-p(x,y)\bigr )\mathrm{d}t,\qquad \mathfrak {R}(z)>0, \end{aligned}$$

where the integral on the right-hand side converges in the distributional sense. Splitting the integral as usual, we may rewrite this as

69 $$\begin{aligned} k_{A^{-z}}(x,y)= & {} \frac{1}{\Gamma (z)}\int _0^1t^{z-1}k_t(x,y)\mathrm{d}t-\frac{1}{\Gamma (z)}\frac{p(x,y)}{z}\nonumber \\&+\,\frac{1}{\Gamma (z)}\int _1^\infty t^{z-1}\bigl (k_t(x,y)-p(x,y)\bigr )\mathrm{d}t,\qquad \mathfrak {R}(z)>0. \end{aligned}$$

In view of (67) the second integral converges with respect to the $C^\infty$-topology and defines a smooth function on $M\times M\times \mathbb {C}$ which depends holomorphically on *z*. If $x\ne y$, then $k_t(x,y)$ vanishes to infinite order at $t=0$, see (47), hence the first integral in (69) converges in the $C^\infty$-topology and defines a smooth function on $\{x\ne y\}\times \mathbb {C}$ which depends holomorphically on *z*.

To study the behavior of

70 $$\begin{aligned} K_z(x,y):=\int _0^1t^{z-1}k_t(x,y)\mathrm{d}t,\qquad \mathfrak {R}(z)>0, \end{aligned}$$

near the diagonal, we fix a real number *s*, use (47), and move to exponential coordinates, see (37) and (38),

71 $$\begin{aligned} (\phi ^*K_z)_x(g)=\int _{t\in [0,1]}t^{z-1}\bigl (\tilde{\phi }^*(k_Q|_{{\tilde{V}}})\bigr )_{(x,s)}(g,t),\qquad \mathfrak {R}(z)>0, \end{aligned}$$

where $x\in M$, $g\in \mathcal {T}_xM$. Given an integer *N*, we choose an integer $j_N$ such that $\tilde{\phi }^*(k_Q|_{{\tilde{V}}})-\sum _{j=0}^{j_N}{\tilde{q}}_j$ is of class $C^N$, see (46), and rewrite (71) in the form

72 $$\begin{aligned} (\phi ^*K_z)_x(g)=\int _{t\in [0,1]}t^{z-1}\left( \tilde{\phi }^* (k_Q|_{{\tilde{V}}})-\sum _{j=0}^{j_N}{\tilde{q}}_j\right) _{(x,s)}(g,t) +\sum _{j=0}^{j_N}(K_{z,j})_x(g)\qquad \end{aligned}$$

where $K_{z,j}$ the distributional section of ${{\,\mathrm{end}\,}}(\pi ^*E)\otimes \Omega _\pi$ defined by

73 $$\begin{aligned} (K_{z,j})_x(g):=\int _{t\in [0,1]}t^{z-1}{\tilde{q}}_{j,(x,s)}(g,t),\qquad \mathfrak {R}(z)>-j/r. \end{aligned}$$

In view of (49) and (33) we have

74 $$\begin{aligned} \bigl (\bigl (\delta ^{{\mathcal {T}}M}_\lambda \bigr )_*K_{z,j}-\lambda ^{-zr-j}K_{z,j}\bigr )_x(g)= \lambda ^{-zr-j}\int _{t\in [1,\lambda ^r]}t^{z-1}{\tilde{q}}_{j,(x,s)}(g,t) \end{aligned}$$

for all $\lambda \ge 1$, and a similar formula holds for $0<\lambda \le 1$. Since the integral on the right-hand side defines a smooth section of ${{\,\mathrm{end}\,}}(E)\otimes \Omega _\pi$, we conclude $K_{z,j}\in \Sigma ^{-zr-j}(E)$. Since the integral term in (72) is of class $C^N$, we have an asymptotic expansion $\phi ^*K_z\sim \sum _{j=0}^\infty K_{z,j}$, provided $\mathfrak {R}(z)>0$. Combining this with (69) and (70) we obtain an asymptotic expansion

75 $$\begin{aligned} \phi ^*(k_{A^{-z}})\sim \sum _{j=0}^\infty \frac{K_{z,j}}{\Gamma (z)}. \end{aligned}$$

This shows $A^{-z}\in \Psi ^{-zr}(E)$, provided $\mathfrak {R}(z)>0$. Using $A^{k-z}=A^kA^{-z}$ and $A^k\in \Psi ^{kr}(E)$ for $k\in \mathbb {N}$, we see that this remains true for all complex *z*.

For $\mathfrak {R}(z)>n/r$, the Heisenberg order of $A^{-z}$ has real part smaller than $-n$, hence this power has a continuous kernel, see [21, Proposition 3.9(d)], and the Mellin formula (66) gives

76 $$\begin{aligned} k_{A^{-z}}(x,x)=\frac{1}{\Gamma (z)}\int _0^\infty t^{z-1}\bigl (k_t(x,x)-p(x,x)\bigr )\mathrm{d}t,\qquad \mathfrak {R}(z)>n/r. \end{aligned}$$

Splitting the integral as usual, we may write

77 $$\begin{aligned} \int _0^\infty t^{z-1}\bigl (k_t(x,x)-p(x,x)\bigr )\mathrm{d}t= & {} \int _1^\infty t^{z-1}\bigl (k_t(x,x)-p(x,x)\bigr )\mathrm{d}t-\frac{p(x,x)}{z}\nonumber \\&+\int _0^1t^{z-1}\left( k_t(x,x)-\sum _{j=0}^{N-1}t^{(j-n)/r}q_j(x)\right) \mathrm{d}t\nonumber \\&+\sum _{j=0}^{N-1}\frac{q_j(x)}{z-(n-j)/r}, \end{aligned}$$

where $q_j\in \Gamma ^\infty \bigl ({{\,\mathrm{end}\,}}(E)\otimes |\Lambda _M|\bigr )$ are as in Theorem 1. In view of the estimate (67), the first integral on the right-hand side of (77) converges for all complex *z* and defines an entire function. In view of Theorem 1, the second integral on the right-hand side of (77) converges for $\mathfrak {R}(z)>(n-N)/r$ and defines a holomorphic function on $\{\mathfrak {R}(z)>(n-N)/r\}$. Note that these considerations are all uniform in *x*. Combining this with (76), we see that $k_{A^{-z}}(x,x)$ can be extended meromorphically to all complex *z* with poles and special values as specified in Theorem 2. Here we also use the classical fact that $1/\Gamma (z)$ is an entire function with zero set $-\mathbb {N}_0$ and $(1/\Gamma )'(-l)=(-1)^ll!$ for all $l\in \mathbb {N}_0$.

To complete the proof of Theorem 2, it remains to show that the powers $A^{-z}$ form a holomorphic family of Heisenberg pseudodifferential operators in a sense analogous to [43, Sect. 4]. This will be established in Sect. 6.

## Holomorphic Families of Heisenberg Pseudodifferential Operators

In this section, we will extend the concept of a holomorphic family of pseudodifferential operators [43, Chapter 4] to the Heisenberg calculus on general filtered manifolds. We will show that the complex powers discussed above do indeed form a holomorphic family as stated in Theorem 2. Holomorphic families will also be used to construct a non-commutative residue in Sect. 7.

### Remark 3

Below we will use several spaces of distributions which are conormal to certain closed submanifolds. It will be convenient to equip theses spaces with the structure of a complete locally convex vector space. To describe this topology, suppose $\xi$ is a vector bundle over a smooth manifold *N*, and suppose *S* is a closed submanifold of *N*. We let $\mathcal {K}\subseteq \Gamma ^{-\infty }(\xi )$ denote the vector space of all distributional sections of $\xi$ whose wave front set is contained in the conormal of *S*. In particular, these distributions are smooth on $N{\setminus } S$, hence we have a canonical map

78 $$\begin{aligned} \mathcal {K}\rightarrow \Gamma ^\infty (\xi |_{N{\setminus } S}). \end{aligned}$$

We let $\pi :T^\perp S\rightarrow S$ denote the normal bundle of *S* in *N*, that is, $T^\perp S:=TN|_S/TS$. Suppose $\varphi :T^\perp S\rightarrow W\subseteq N$ is a tubular neighborhood, i.e., a diffeomorphism onto an open neighborhood *W* of *S* in *N*, which restricts to the identity along *S*. Moreover, let $\phi$ be a vector bundle isomorphism $\pi ^*(\xi |_S)\cong \varphi ^*(\xi |_W)$. If $a\in \mathcal {K}$, then $\phi ^*a$ is a distributional sections of $\pi ^*(\xi |_S)$ whose wave front set is conormal to the zero section $S\subseteq T^\perp S$. In particular, $\phi ^*a$ is $\pi$-fibered. Hence, we obtain a map

79 $$\begin{aligned} \phi ^*:\mathcal {K}\rightarrow \Gamma ^{-\infty }_\pi (\pi ^*(\xi |_S)), \end{aligned}$$

where the right-hand side denotes the space of all $\pi$-fibered distributional sections of $\pi ^*(\xi |_S)$. Recall that elements $a\in \Gamma ^{-\infty }_\pi (\pi ^*(\xi |_S))$ can be considered as families of distributions $a_x\in C^{-\infty }(T^\perp _xS,\xi _x)$ on the fibers $T_x^\perp S$ with values in $\xi _x$ which depend smoothly on $x\in S$. Every $\chi \in \Gamma ^\infty _c(\Omega _\pi )$ provides a map $\Gamma ^{-\infty }_\pi (\pi ^*(\xi |_S))\rightarrow \Gamma ^\infty (\xi |_S)$, $a\mapsto \pi _*(a\chi )$, where $\pi _*$ denotes integration along the fibers of $\pi$. We equip $\Gamma ^{-\infty }_\pi (\pi ^*(\xi |_S))$ with the weakest locally convex topology such that these maps are all continuous. Finally, we equip $\mathcal {K}$ with the coarsest locally convex topology such that the maps (78) and (79) become continuous. It is well known that $\mathcal {K}$ is complete. Moreover, the topology does not depend on the choice of a tubular neighborhood.

Suppose *E* and *F* are two vector bundles over a filtered manifold *M*. Moreover, let $\Omega$ be a domain in the complex plane and suppose $s:\Omega \rightarrow \mathbb {C}$ is a holomorphic function. We intend to make precise when a family of operators $A_z\in \Psi ^{s(z)}(E,F)$, parametrized by $z\in \Omega$, is considered to be a holomorphic family.

Recall from Sect. 3 that $\mathcal {K}(\mathbb {T}M;E,F)$ denotes the space of all distributional sections of the vector bundle (19) whose wave front set is contained in the conormal of the units, $M\times \mathbb {R}\subseteq \mathbb {T}M$. We equip $\mathcal {K}(\mathbb {T}M;E,F)$ with the topology described in Remark 3, and let $\mathcal {K}_\Omega (\mathbb {T}M;E,F)$ denote the space of holomorphic curves from $\Omega$ into $\mathcal {K}(\mathbb {T}M;E,F)$. Moreover, we let $\mathcal {K}_\Omega ^\infty (\mathbb {T}M;E,F)$ denote the space of holomorphic curves from $\Omega$ into $\mathcal {K}^\infty (\mathbb {T}M;E,F)$ where the latter space carries the $C^\infty$-topology.

### Definition 3

Let $\Omega$ be a domain in the complex plane and suppose $s:\Omega \rightarrow \mathbb {C}$ is a holomorphic function. A family of operators $A_z\in \Psi ^{s(z)}(E,F)$, parametrized by $z\in \Omega$, is called a *holomorphic family of Heisenberg pseudodifferential operators* if there exists a family $\mathbb {K}_z\in \mathcal {K}_\Omega (\mathbb {T}M;E,F)$ such that ${{\,\mathrm{ev}\,}}_1(\mathbb {K}_z)$ is the Schwartz kernel of $A_z$ for all $z\in \Omega$, and $\mathbb {K}_z$ is essentially homogeneous of order *s*(*z*) in the sense that $(\delta ^{\mathbb {T}M}_\lambda )_*\mathbb {K}_z-\lambda ^{s(z)}\mathbb {K}_z$ is a family in $\mathcal {K}^\infty _\Omega (\mathbb {T}M;E,F)$, for all $\lambda >0$.

### Lemma 6

Suppose $A_z\in \Psi ^{s(z)}(E,F)$ and $B_z\in \Psi ^{t(z)}(F,G)$ are two holomorphic families of Heisenberg pseudodifferential operators, where $z\in \Omega$ and $s,t:\Omega \rightarrow \mathbb {C}$. Then $B_zA_z\in \Psi ^{(t+s)(z)}(E,G)$ is a holomorphic family of Heisenberg pseudodifferential operators, provided at least one of the two families is properly supported (locally uniformly in *z*). Moreover, the transpose $A_z^t\in \Psi ^{s(z)}(F',E')$ is a holomorphic family of Heisenberg pseudodifferential operators.

### Proof

Convolution and transposition induce bounded (bi)linear maps:

$$\begin{aligned} \mathcal {K}(\mathbb {T}M;F,G)\times \mathcal {K}_\text {prop}(\mathbb {T}M;E,F)&\rightarrow \mathcal {K}(\mathbb {T}M;E,G)\\ \mathcal {K}^\infty (\mathbb {T}M;F,G)\times \mathcal {K}_\text {prop}(\mathbb {T}M;E,F)&\rightarrow \mathcal {K}^\infty (\mathbb {T}M;E,G)\\ \mathcal {K}(\mathbb {T}M;F,G)\times \mathcal {K}_\text {prop}^\infty (\mathbb {T}M;E,F)&\rightarrow \mathcal {K}^\infty (\mathbb {T}M;E,G)\\ \mathcal {K}^\infty (\mathbb {T}M;F,G)\times \mathcal {K}_\text {prop}^\infty (\mathbb {T}M;E,F)&\rightarrow \mathcal {K}^\infty (\mathbb {T}M;E,G)\\ \mathcal {K}(\mathbb {T}M;E,F)&\rightarrow \mathcal {K}(\mathbb {T}M;F',E')\\ \mathcal {K}^\infty (\mathbb {T}M;E,F)&\rightarrow \mathcal {K}^\infty (\mathbb {T}M;F',E'). \end{aligned}$$

Since holomorphic curves remain holomorphic when composed with bounded (bi)linear mappings, convolution, and transposition thus induce maps:

$$\begin{aligned} \mathcal {K}_\Omega (\mathbb {T}M;F,G)\times \mathcal {K}_{\Omega ,\text {prop}}(\mathbb {T}M;E,F)&\rightarrow \mathcal {K}_\Omega (\mathbb {T}M;E,G)\\ \mathcal {K}_\Omega ^\infty (\mathbb {T}M;F,G)\times \mathcal {K}_{\Omega ,\text {prop}}(\mathbb {T}M;E,F)&\rightarrow \mathcal {K}_\Omega ^\infty (\mathbb {T}M;E,G)\\ \mathcal {K}_\Omega (\mathbb {T}M;F,G)\times \mathcal {K}^\infty _{\Omega ,\text {prop}}(\mathbb {T}M;E,F)&\rightarrow \mathcal {K}_\Omega ^\infty (\mathbb {T}M;E,G)\\ \mathcal {K}_\Omega ^\infty (\mathbb {T}M;F,G)\times \mathcal {K}^\infty _{\Omega ,\text {prop}}(\mathbb {T}M;E,F)&\rightarrow \mathcal {K}_\Omega ^\infty (\mathbb {T}M;E,G)\\ \mathcal {K}_\Omega (\mathbb {T}M;E,F)&\rightarrow \mathcal {K}_\Omega (\mathbb {T}M;F',E')\\ \mathcal {K}_\Omega ^\infty (\mathbb {T}M;E,F)&\rightarrow \mathcal {K}_\Omega ^\infty (\mathbb {T}M;F',E'). \end{aligned}$$

The lemma follows at once. $\square$

Recall from Sect. 3 that $\mathcal {K}({\mathcal {T}}M;E,F)$ denotes the space of all distributional sections of the vector bundle $\hom (\pi ^*E,\pi ^*F)\otimes \Omega _\pi$ over ${\mathcal {T}}M$ whose wave front set is contained in the conormal of the units, $M\subseteq {\mathcal {T}}M$. We equip $\mathcal {K}({\mathcal {T}}M;E,F)$ with the topology described in Remark 3, and let $\mathcal {K}_\Omega ({\mathcal {T}}M;E,F)$ denote the space of holomorphic curves from $\Omega$ into $\mathcal {K}({\mathcal {T}}M;E,F)$. Moreover, we let $\mathcal {K}_\Omega ^\infty ({\mathcal {T}}M;E,F)$ denote the space of holomorphic curves from $\Omega$ into $\mathcal {K}^\infty ({\mathcal {T}}M;E,F)$ where the latter space carries the $C^\infty$-topology.

### Definition 4

Let $\Omega$ be a domain in the complex plane, and suppose $s:\Omega \rightarrow \mathbb {C}$ is a holomorphic function. A family $k_z\in \mathcal {K}_\Omega ({\mathcal {T}}M;E,F)$ is called *essentially homogeneous* of order *s*(*z*) if $(\delta _\lambda )_*k_z-\lambda ^{s(z)}k_z\in \mathcal {K}_\Omega ^\infty ({\mathcal {T}}M;E,F)$, for all $\lambda >0$. This generalizes [43, Definition 4.4.1] where the term *almost homogeneous* is used.

### Lemma 7

Let $s:\Omega \rightarrow \mathbb {C}$ be a holomorphic function, consider a family of operators $A_z\in \Psi ^{s(z)}(E,F)$ parametrized by $z\in \Omega$, and let $k_z$ denote the Schwartz kernel of $A_z$. Then the following are equivalent:

- $A_z$ is a holomorphic family of Heisenberg pseudodifferential operators.
- Away from the diagonal, the Schwartz kernels $k_z$ are smooth and depend holomorphically on $z\in \Omega$. Moreover, with respect to some (and then every) exponential coordinates adapted to the filtration as in Sect. 3, see (16), we have an asymptotic expansion of the form
  80 $$\begin{aligned} \phi ^*(k_z|_V)\sim \sum _{j=0}^\infty k_{j,z} \end{aligned}$$
  where $k_{j,z}\in \mathcal {K}_\Omega ({\mathcal {T}}M;E,F)$ is an essentially homogeneous family of order $s(z)-j$, cf. Definition 4. More precisely, for every $z_0\in \Omega$ and every integer *N* there exists a neighborhood *W* of $z_0$ in $\Omega$ such that, for sufficiently large $J\in \mathbb {N}$, the expression $\phi ^*(k_z|_V)-\sum _{j=0}^{J-1}k_{j,z}$ restricts to a holomorphic curve from *W* into the space of $C^N$-sections of $\hom (\pi ^*E,\pi ^*F)\otimes \Omega _\pi$ over *U*.
- Away from the diagonal, the Schwartz kernels $k_z$ are smooth and depend holomorphically on $z\in \Omega$. Moreover, with respect to some (and then every) exponential coordinates adapted to the filtration as in Sect. 3, see (16), and for some (and then any) properly supported bump function $\chi \in C^\infty _\text {prop}({\mathcal {T}}M)$ with ${{\,\mathrm{supp}\,}}(\chi )\subseteq U$ and $\chi \equiv 1$ in a neighborhood of the zero section, the fiber wise Fourier transform,
  $$\begin{aligned} \mathcal {F}\bigl (\chi \cdot \phi ^*(k_z|_V)\bigr )(\xi ) =\int _{X\in \mathfrak t_xM}e^{-2\pi \mathbf i\langle \xi ,X\rangle }\bigl (\chi \cdot \phi ^*(k_z|_V)\bigr )(\exp (X)),\quad \xi \in \mathfrak t_x^*M, \end{aligned}$$
  is smooth on $\Omega \times \mathfrak t^*M$, depends holomorphically on *z*, and admits a uniform asymptotic expansion of the form
  81 $$\begin{aligned} \mathcal {F}\bigl (\chi \cdot \phi ^*(k_z|_V)\bigr )\sim \sum _{j=0}^\infty {\hat{k}}_{j,z}, \end{aligned}$$
  where ${\hat{k}}_{j,z}\in \Gamma ^\infty (\hom (p^*E,p^*F)|_{\mathfrak t^*M{\setminus } M})$ are homogeneous of order $s(z)-j$, i.e., $(\dot{\delta }'_\lambda )^*{\hat{k}}_{j,z}=\lambda ^{s(z)-j}{\hat{k}}_{j,z}$ for all $\lambda >0$. Here $\dot{\delta }_\lambda '$ denotes the grading automorphism on $\mathfrak t^*M$ dual to $\dot{\delta }_\lambda$ on $\mathfrak tM$, and $p:\mathfrak t^*M\rightarrow M$ denotes the vector bundle projection. More precisely, for all $a,N\in \mathbb {N}$, and for every homogeneous differential operator *D* acting on $\Gamma ^\infty (\hom (p^*E,p^*F))$, i.e., $(\dot{\delta }'_\lambda )_*D=\lambda ^aD$, for every compact $K\subseteq \Omega$, and for some (and then every) fiberwise homogeneous norm on $\mathfrak t^*M$, and for some (and then every) fiberwise Hermitian metric on $\hom (E,F)$, there exists a constant $C\ge 0$ such that
  82 $$\begin{aligned} \left| D\left( \mathcal {F}\bigl (\chi \cdot \phi ^*(k_z|_V)\bigr )-\sum _{j=0}^{N-1}{\hat{k}}_{j,z}\right) (\xi )\right| \le C|\xi |^{s(z)-N-a} \end{aligned}$$
  holds for all $z\in K$ and all $\xi \in \mathfrak t^*M$ with $|\xi |\ge 1$.

### Proof

To see (a)$\Rightarrow$(b) assume $A_z$ is a holomorphic family of Heisenberg pseudodifferential operators with Schwartz kernels $k_z$. Choose $\mathbb {K}\in \mathcal {K}_\Omega (\mathbb {T}M;E,F)$ as in Definition 3, i.e., ${{\,\mathrm{ev}\,}}_1(\mathbb {K}_z)=k_z$ and $(\delta ^{\mathbb {T}M}_\lambda )_*\mathbb {K}_z-\lambda ^{s(z)}\mathbb {K}_z\in \mathcal {K}^\infty _\Omega (\mathbb {T}M;E,F)$ for all $\lambda >0$. Away from the units, $\mathbb {K}_z$ is smooth and depends holomorphically on $z\in \Omega$. Clearly, this implies that the same is true for $k_z$, i.e., away from the diagonal $k_z$ is smooth and depends holomorphically on $z\in \Omega$. Now consider adapted exponential coordinates as in Sect. 3. By conormality, $\mathbb {K}$ has a Taylor expansion along $\mathcal {T}^\text {op}M\times \{0\}$,

83 $$\begin{aligned} \Phi ^*\bigl (\mathbb {K}_z|_{\mathbb {V}}\bigr )\sim \sum _{j=0}^\infty \mathbb {K}_{j,z}t^j \end{aligned}$$

where $\mathbb {K}_{j,z}\in \mathcal {K}_\Omega (\mathcal {T}^\text {op}M;E,F)$. By essential homogeneity of $\mathbb {K}$, the family $\mathbb {K}_{j,z}$ is essentially homogeneous of order $s(z)-j$, that is, $(\delta _\lambda )_*\mathbb {K}_{j,z}-\lambda ^{s(z)-j}\mathbb {K}_{j,z}\in \mathcal {K}^\infty _\Omega (\mathcal {T}^\text {op}M;E,F)$. For each $J\in \mathbb {N}$ we have $\Phi ^*\bigl (\mathbb {K}_z|_{\mathbb {V}}\bigr )-\sum _{j=0}^{J-1}\mathbb {K}_{j,z}t^j=t^J\mathbb {L}_{J,z}$ where $\mathbb {L}_{J,z}\in \mathcal {K}_\Omega (\mathbb {U};E,F)$ is essentially homogeneous of order $s(z)-J$ on $\mathbb {U}$. Let *W* be an open subset with compact closure in $\Omega$, and suppose $N\in \mathbb {N}$. Then, for sufficiently large *J*, the family $\mathbb {L}_{J,z}$ restricts to a holomorphic curve from *W* into the space of $C^N$-sections over $\mathbb {U}$. Indeed, this follows from a parametrized version of [56, Theorem 52]. We conclude that $\Phi ^*\bigl (\mathbb {K}_z|_{\mathbb {V}}\bigr )-\sum _{j=0}^{J-1}\mathbb {K}_{j,z}t^j$ restricts to a holomorphic curve from *W* into the space of $C^N$-sections over $\mathbb {U}$. Evaluating at $t=1$, we obtain the asymptotic expansion (80) with $k_{j,z}=\nu ^*(\mathbb {K}_{j,z})$.

To see (b)$\Rightarrow$(a) we observe that $t^jk_{j,z}\in \mathcal {K}_\Omega (\mathbb {U};E,F)$ is essentially homogeneous of order *s*(*z*). Parametrizing the proof of [56, Theorem 59], we obtain a holomorphic family $\mathbb {L}_z\in \mathcal {K}_\Omega (\mathbb {U};E,F)$ which is essentially homogeneous of order *s*(*z*) and such that ${{\,\mathrm{ev}\,}}_1(\mathbb {L}_z)\sim \sum _{j=0}^\infty k_{j,z}$ in the same sense as (80). Clearly, this implies that $\phi ^*(k_z|_V)-{{\,\mathrm{ev}\,}}_1(\mathbb {L}_z)$ is a holomorphic curve into the space of smooth sections over $\mathbb {U}$. Adding a term in $\mathcal {K}_\Omega ^\infty (\mathbb {U};E,F)$ to $\mathbb {L}_z$, we may, moreover, assume $\phi ^*(k_z|_V)={{\,\mathrm{ev}\,}}_1(\mathbb {L}_z)$. Using a bump function one readily constructs $\mathbb {K}_z\in \mathcal {K}_\Omega (\mathbb {T}M;E,F)$, essentially homogeneous of order *s*(*z*), such that $\Phi ^*(\mathbb {K}_z)$ coincides with $\mathbb {L}_z$ on neighborhood of $M\times \mathbb {R}$. Hence, $k_z$ coincides with ${{\,\mathrm{ev}\,}}_1(\mathbb {K}_z)$ in a neighborhood of the diagonal. Clearly, this implies (a).

Let us now turn to the implication (b)$\Rightarrow$(c): Note first, that $\chi \cdot \phi ^*(k_z|_V)\in \mathcal {K}_{\Omega ,\text {prop}}({\mathcal {T}}M;E,F)$, hence its fiberwise Fourier transform is smooth on $\mathfrak t^*M\times \Omega$ and depends holomorphically on $z\in \Omega$. Fix a bump function $\rho \in C^\infty (\mathfrak t^*M)$ which vanishes in a neighborhood of the zero section and such that $\rho (\xi )=1$ whenever $|\xi |\ge 1$. Since $k_{z,j}$ is essentially homogeneous of order $s(z)-j$, there exist unique ${\hat{k}}_{j,z}\in \Gamma ^\infty (\hom (p^*E,p^*F)|_{\mathfrak t^*M{\setminus } M})$, strictly homogeneous of order $s(z)-j$, such that $\rho \cdot \bigl (\mathcal {F}(\chi \cdot k_{z,j})-{\hat{k}}_{z,j}\bigr )$ is in the fiberwise Schwartz space $\mathcal {S}(\mathfrak t^*M;\hom (p^*E,p^*F))$. More precisely, for every compact $K\subseteq \Omega$, every homogeneous differential operator *D*, and every integer $b\in \mathbb {N}$, there exists a constant $C\ge 0$ such that

$$\begin{aligned} \left| D\left( \mathcal {F}\bigl (\chi \cdot k_{j,z}\bigr )-{\hat{k}}_{j,z}\right) (\xi )\right| \le C|\xi |^{-b} \end{aligned}$$

holds for all $z\in K$ and $\xi \in \mathfrak t^*M$ with $|\xi |\ge 1$. In view of the expansion (80), we conclude that for every compact $K\subseteq \Omega$, every $b\in \mathbb {N}$, every homogeneous differential operator *D*, and every sufficiently large $J\in \mathbb {N}$, there exists a constant $C\ge 0$ such that

$$\begin{aligned} \left| D\left( \mathcal {F}\left( \chi \cdot \left( \phi ^*(k_z|_V)-\sum _{j=0}^{J-1}k_{j,z}\right) \right) \right) (\xi )\right| \le C|\xi |^{-b} \end{aligned}$$

holds for all $z\in K$ and $\xi \in \mathfrak t^*M$ with $|\xi |\ge 1$. Combining the preceding two estimates, we see that for every compact $K\subseteq \Omega$, every $b\in \mathbb {N}$, every homogeneous differential operator *D*, and every sufficiently large $J\in \mathbb {N}$, there exists a constant $C\ge 0$ such that

$$\begin{aligned} \left| D\left( \mathcal {F}\bigl (\chi \cdot \phi ^*(k_z|_V)\bigr )-\sum _{j=0}^{J-1}{\hat{k}}_{j,z}\right) (\xi )\right| \le C|\xi |^{-b} \end{aligned}$$

holds for all $z\in K$ and $\xi \in \mathfrak t^*M$ with $|\xi |\ge 1$. If *D* is homogeneous of degree $a\in \mathbb {N}$, i.e., $(\dot{\delta }'_\lambda )_*D=\lambda ^aD$, then $|D{\hat{k}}_{j,z}(\xi )|\le C|\xi |^{s(z)-j-a}$, whence (81).

Let us now turn to the implication (c)$\Rightarrow$(b): Note first that the homogeneous terms ${\hat{k}}_{j,z}$ in the asymptotic expansion (81) depend holomorphically on $z\in \Omega$. This follows from (82), cf. [43, Remark 4.2.2]. Defining $k_{j,z}:=\mathcal {F}^{-1}(\rho \cdot {\hat{k}}_{j,z})$, we thus have $k_{j,z}\in \mathcal {K}_\Omega ({\mathcal {T}}M;E,F)$. Moreover, for each $\lambda >0$,

$$\begin{aligned} (\delta _\lambda )_*k_{j,z}-\lambda ^{s(z)-j}k_{j,z} =\mathcal {F}^{-1}\left( \lambda ^{s(z)-j}\cdot \bigl ((\dot{\delta }'_\lambda )^*\rho -\rho \bigr )\cdot {\hat{k}}_{j,z}\right) \end{aligned}$$

is a holomorphic curve in the fiberwise Schwartz space $\mathcal {S}({\mathcal {T}}M;\hom (\pi ^*E,\pi ^*F)\otimes \Omega _\pi )$. In particular, $k_{j,z}$ is essentially homogeneous of order $s(z)-j$. If *W* is an open subset with compact closure in $\Omega$, then (82) implies that, for sufficiently large $J\in \mathbb {N}$, the expression $\chi \cdot \phi ^*(k_z|_V)-\sum _{j=0}^{J-1}k_{j,z}$ is a holomorphic curve in the space of $C^N$ sections, whence (80). $\square$

### Remark 4

As pointed out in the proof above, the homogeneous terms ${\hat{k}}_{j,z}$ in the asymptotic expansion (81) depend holomorphically on $z\in \Omega$.

The characterization in Lemma 7 shows that the concept of holomorphic families considered here generalizes the definition for Heisenberg manifolds, see [43, Sect. 4].

Let us now complete the proof of Theorem 2 by establishing the following generalization of [43, Theorem 5.3.1].

### Lemma 8

The complex powers $A^{-z}\in \Psi ^{-zr}(E)$ considered in Theorem 2 constitute a holomorphic family.

### Proof

Writing $A^{-z}=A^kA^{-(z+k)}$ and using Lemma 6, we see that it suffices to show that the powers form a holomorphic family on $\Omega =\{\mathfrak {R}(z)>0\}$. To prove this, we will use the characterization in Lemma 7(b) and proceed as in [43, Lemma 5.3.2].

As observed above, away from the diagonal the Schwartz kernels $k_{A^{-z}}$, see (69), are smooth and depend holomorphically on $z\in \mathbb {C}$. Moreover, the distributions $K_{z,j}$ defined in (73) form a family in $\mathcal {K}_\Omega ({\mathcal {T}}M;E,F)$ which is essentially homogeneous of order $-rz-j$ in the sense of Definition 4, see (74). Furthermore, the $C^N$ sections defined by the integral in (72) depend holomorphically on $z\in \Omega$. We conclude that the asymptotic expansion (75) established above is in fact the asymptotic expansion required in Lemma 7(b). $\square$

## Non-commutative Residue

In this section, we consider the analogue of Wodzicki’s non-commutative residue [29, 58] for filtered manifolds. Let *E* be a vector bundle over a closed filtered manifold *M* of homogeneous dimension *n*. We will fix a strictly positive Rockland differential operator $D\in \mathcal {D}\mathcal {O}^r(E)$ of even Heisenberg order $r>0$. For a pseudodifferential operator $A\in \Psi ^k(E)$ of integer Heisenberg order $k\in \mathbb {Z}$, the operator $AD^{-z}\in \Psi ^{k-rz}(E)$ is trace class for $\mathfrak R(z)>(n+k)/r$ and hence defines a holomorphic function

$$\begin{aligned} \zeta _A(z):={{\,\mathrm{tr}\,}}(AD^{-z}),\qquad \mathfrak R(z)>(n+k)/r, \end{aligned}$$

see Theorem 2 and [21, Proposition 3.9(d)]. In view of Lemma 6, the subsequent proposition can be applied to $R(z)=AD^{-z}$.

We continue to use the canonical identification $|\Lambda _M|=o^*\Omega _\pi$ between the bundle of 1-densities on *M* and the restriction along the zero section of the bundle of vertical densities on ${\mathcal {T}}M$, see Sect. 3. In particular, we will use the induced identification

84 $$\begin{aligned} \Gamma ^\infty (|\Lambda _M|\otimes {{\,\mathrm{end}\,}}(E))=\mathcal {P}^{-n}({\mathcal {T}}M;E,E). \end{aligned}$$

Recall that the right-hand side in (84) denotes the space of smooth sections of ${{\,\mathrm{end}\,}}(\pi ^*E)\otimes \Omega _\pi$ which are homogeneous of degree $-n$ and thus fiberwise translation invariant (constant).

### Proposition 2

Let *E* be a vector bundle over a closed filtered manifold *M* of homogeneous dimension *n*. Moreover, suppose $r>0$ and let $R(z)\in \Psi ^{d-rz}(E)$ be a holomorphic family of Heisenberg pseudodifferential operators on *M* with Schwartz kernels $k_z$. Then the restriction to the diagonal, $k_z(x,x)$, provides a holomorphic curve in $\Gamma ^\infty ({{\,\mathrm{end}\,}}(E)\otimes |\Lambda _M|\bigr )$ for $\mathfrak {R}(z)>(n+d)/r$. This curve can be extended meromorphically to the entire complex plane with at most simple poles located at $z_j:=(n+d-j)/r$ with $j\in \mathbb {N}_0$. Moreover, the residue at $z_j$ can be computed locally by the expected formula,

85 $$\begin{aligned} {{\,\mathrm{res}\,}}_{z=z_j}\bigl (k_z(x,x)\bigr )=\frac{1}{r}\int _{\xi \in \mathfrak t_x^*M,|\xi |=1}{\hat{k}}_{j,z_j}(\xi )\mathrm{d}\xi =\frac{p_j(o_x)}{r}, \end{aligned}$$

where ${\hat{k}}_{j,z}$ are as in Lemma 7(c), and $p_j\in \mathcal {P}^{-n}({\mathcal {T}}M;E,E)$ are characterized by $(\delta _\lambda )_*k_{j,z_j}=\lambda ^{-n}k_{j,z_j}+\lambda ^{-n}\log |\lambda |\,p_j$ for $\lambda >0$ with $k_{j,z}$ as in Lemma 7(b).^4^

In particular, the function ${{\,\mathrm{tr}\,}}(R(z))$ is analytic on the half plane $\mathfrak R(z)>(n+d)/r$, and has a meromorphic continuation to all of the complex plane with only simple poles possibly located at $z_j$ with $j\in \mathbb {N}_0$. Moreover,

86 $$\begin{aligned} {{\,\mathrm{res}\,}}_{z=z_j}{{\,\mathrm{tr}\,}}(R(z))=\frac{1}{r}\int _{x\in M}\int _{\xi \in \mathfrak t_x^*M,|\xi |=1}{{\,\mathrm{tr}\,}}_E\bigl ({\hat{k}}_{j,z_j}(\xi )\bigr )\mathrm{d}\xi =\frac{1}{r}\int _M{{\,\mathrm{tr}\,}}_E(p_j|_M).\qquad \end{aligned}$$

### Remark 5

The volume density $\mathrm{d}\xi$ used in (85) and (86) is defined such that a 1-density on $\mathfrak t_xM$ in the Schwartz class, $k\in \mathcal {S}(|\Lambda _{\mathfrak t_xM}|)$, and its Fourier transform, ${\hat{k}}\in \mathcal {S}(\mathfrak t_x^*M)$, a Schwartz class function on $\mathfrak t_x^*M$, are related by

87 $$\begin{aligned} {\hat{k}}(\xi )=\int _{X\in \mathfrak t_xM}e^{-2\pi \mathbf i\langle \xi ,X\rangle }k \qquad \text {and}\qquad k(X)=\int _{\mathfrak t_x^*M}e^{2\pi \mathbf i\langle \xi ,X\rangle }{\hat{k}}(\xi )\mathrm{d}\xi , \end{aligned}$$

where $X\in \mathfrak t_xM$ and $\xi \in \mathfrak t_x^*M$. Hence, $\mathrm{d}\xi$ is the translation invariant 1-density on $\mathfrak t_x^*M$ with values in $|\Lambda _{\mathfrak t_xM}|$ corresponding to the canonical pairing between $|\Lambda _{\mathfrak t_x^*M}|$ and $|\Lambda _{\mathfrak t_xM}|$. Given a homogeneous norm $|-|$ on $\mathfrak t_xM$, we may contract $\mathrm{d}\xi$ with the fundamental vector field of the dilation action, $\frac{\partial }{\partial \lambda }|_{\lambda =1}\dot{\delta }'_\lambda$, and restrict to obtain a 1-density on the unit sphere $\{\xi \in \mathfrak t_x^*M:|\xi |=1\}$ with values in $|\Lambda _{\mathfrak t_xM}|$ which will also be denoted by $\mathrm{d}\xi$ and can be characterized by

88 $$\begin{aligned} \int _{\mathfrak t_x^*M}f(\xi )\mathrm{d}\xi =\int _0^\infty \lambda ^{n-1}d\lambda \int _{\xi \in \mathfrak t_x^*M,|\xi |=1}f(\dot{\delta }_\lambda '(\xi ))\mathrm{d}\xi , \end{aligned}$$

for all compactly supported smooth functions *f* on $\mathfrak t_x^*M{\setminus }\{0\}$.

### Proof of Proposition 2

To begin with, let $\mathfrak {R}(z)>(n+d)/r$. We fix adapted exponential coordinates and consider the corresponding asymptotic expansion of the kernel $k_z$ along the diagonal as in Lemma 7(c), see (81). Using Fourier inversion formula, see (87), we may write

$$\begin{aligned} k_z(x,x) =\bigl (\phi ^*(k_z|_V)\bigr )(o_x) =\int _{\mathfrak t^*_xM}\mathcal {F}\bigl (\chi \cdot \phi ^*(k_z|_V)\bigr )(\xi )\mathrm{d}\xi . \end{aligned}$$

Splitting the integral at the unit sphere in $\mathfrak t_x^*M$ and using the asymptotic expansion in (81) this may be rewritten in the form

89 $$\begin{aligned} k_z(x,x)&=\int _{\xi \in \mathfrak t_x^*M,|\xi |\le 1}\mathcal {F}\bigl (\chi \cdot \phi ^*(k_z|_V)\bigr )(\xi )\mathrm{d}\xi \nonumber \\&\quad +\int _{\xi \in \mathfrak t_x^*M, |\xi |\ge 1}\left( \mathcal {F}\bigl (\chi \cdot \phi ^*(k_z|_V)\bigr )-\sum _{j=0}^N{\hat{k}}_{j,z}\right) (\xi )\mathrm{d}\xi \\&\quad +\sum _{j=0}^N\int _{\xi \in \mathfrak t_x^*M,|\xi |\ge 1}{\hat{k}}_{j,z}(\xi )\mathrm{d}\xi .\nonumber \end{aligned}$$

The continuation of the left-hand side in (89) can be achieved by studying the right-hand side. Clearly, the first integral on the right-hand side of (89) converges and defines a smooth section of ${{\,\mathrm{end}\,}}(E)\otimes |\Lambda _M|$ which depends holomorphically on $z\in \Omega$ for the integrand is smooth and analytic in *z*. The second integral converges for $\mathfrak {R}(z)>(d+n-N)/r$ and defines a smooth section of ${{\,\mathrm{end}\,}}(E)\otimes |\Lambda _M|$ which depends holomorphically on these *z* in view of the estimates (82). Using (88) and the homogeneity of ${\hat{k}}_{j,z}$, we rewrite the remaining terms in the form

$$\begin{aligned} \int _{\xi \in \mathfrak t_x^*M,|\xi |\ge 1}{\hat{k}}_{j,z}(\xi )\mathrm{d}\xi= & {} \int _1^\infty \lambda ^{d-rz-j+n-1}d\lambda \int _{\xi \in \mathfrak t_x^*M,|\xi |=1}{\hat{k}}_{j,z}(\xi )\mathrm{d}\xi \\= & {} \frac{1}{rz-d-n+j}\int _{\xi \in \mathfrak t_x^*M,|\xi |=1}{\hat{k}}_{j,z}(\xi )\mathrm{d}\xi . \end{aligned}$$

Hence, this term admits a meromorphic continuation to the entire complex plane with a single pole located at $z_j=(d+n-j)/r$. This pole is simple with residue

$$\begin{aligned} {{\,\mathrm{res}\,}}_{z=z_j}\int _{\xi \in \mathfrak t_x^*M,|\xi |\ge 1}{\hat{k}}_{j,z}(\xi )\mathrm{d}\xi =\frac{1}{r}\int _{\xi \in \mathfrak t_x^*M,|\xi |=1}{\hat{k}}_{j,z_j}(\xi )\mathrm{d}\xi . \end{aligned}$$

Combining these observation, we obtain the meromorphic extension of $k_z(x,x)$ and the first equality in (85). The second equality in (85) is well known.

For $\mathfrak R(z)>(n+d)/r$ the Schwartz kernel $k_z$ of the operator *R*(*z*) is continuous, and we can compute the trace by

$$\begin{aligned} {{\,\mathrm{tr}\,}}(R(z))=\int _{x\in M}{{\,\mathrm{tr}\,}}_E(k_z(x,x)). \end{aligned}$$

The meromorphic extension of ${{\,\mathrm{tr}\,}}(R(z))$ thus follows from the meromorphic extension of $k_z(x,x)$, and so does the formula for the residues. $\square$

We can now define a functional on integer-order pseudodifferential operators $A\in \Psi ^\infty (E):=\bigcup _{k\in \mathbb {Z}}\Psi ^k(E)$ by

$$\begin{aligned} \tau (A):=r\cdot {{\,\mathrm{res}\,}}_{z=0}(\zeta _A(z)), \end{aligned}$$

where $\zeta _A(z)={{\,\mathrm{tr}\,}}(AD^{-z})$, cf. Proposition 2 and Lemma 6.

The following corollary generalizes a result for CR manifolds obtained by R. Ponge in his thesis, see [42].

### Corollary 6

The functional $\tau :\Psi ^{\infty }(E)\rightarrow \mathbb {C}$ is a non-trivial trace on the algebra of integer-order pseudodifferential operators. More precisely, we have:

- $$\tau ([A,B])=0$$ for all $$A,B\in \Psi ^\infty (E)$$.
- $\tau (A)$ does not depend on the positive Rockland differential operator *D*.
- If the order of $A\in \Psi ^\infty (E)$ is less than $-n$ then $\tau (A)=0$.
- If *A* has order $d\ge -n$, and $\phi ^*(k_A|_V)\sim \sum _{j=0}^\infty k_j$ is an asymptotic expansion of its kernel with $k_j$ essentially homogeneous of order $d-j$, chosen such that $(\delta _\lambda )_*k_{d+n}=\lambda ^{-n}k_{d+n}+\lambda ^{-n}\log |\lambda |p_A$ for $\lambda >0$ with $p_A\in \mathcal {P}^{-n}({\mathcal {T}}M;E,E)$, then
  $$\begin{aligned} \tau (A)=\int _M{{\,\mathrm{tr}\,}}_E(p_A|_M). \end{aligned}$$
- If $B\in \Gamma ^\infty ({{\,\mathrm{end}\,}}(E))$ and $j\in \mathbb {N}_0$, then
  90 $$\begin{aligned} \tau \left( BD^{(j-n)/r}\right) =\frac{r}{\Gamma ((n-j)/r)}\int _M{{\,\mathrm{tr}\,}}_E(Bq_j), \end{aligned}$$
  where $q_j$ denotes the term in the heat kernel asymptotics of *D*, that is $k_{e^{-tD}}(x,x)\sim \sum _{j=0}^\infty t^{(j-n)/r}q_j(x)$, see Theorem 1.
- The map $\mu :C^\infty (M)\rightarrow \mathbb {C}$, $\mu (f):=\tau (fD^{-\frac{n}{r}})=\frac{r}{\Gamma (n/r)}\int _Mf{{\,\mathrm{tr}\,}}_E(q_0)$ is a positive smooth measure on *M*.
- In particular, $\mu (1)=\tau (D^{-\frac{n}{r}})=r\cdot a_0/\Gamma (\frac{n}{r})>0$, where $a_0$ is the leading term in the heat trace expansion of *D*, that is, ${{\,\mathrm{tr}\,}}(e^{-tD})\sim \sum _{j=0}^\infty t^{(j-n)/r}a_j$, see Corollary 1.

### Proof

To see (a), we observe that, for $\mathfrak {R}(z)$ very large,

$$\begin{aligned} {{\,\mathrm{tr}\,}}([A,B]D^{-z})={{\,\mathrm{tr}\,}}(A[B,D^{-z}])+{{\,\mathrm{tr}\,}}([AD^{-z},B])={{\,\mathrm{tr}\,}}(A[B,D^{-z}]). \end{aligned}$$

Note that $[B,D^{-z}]$ is a holomorphic family which vanishes at $z=0$, for *D* is assumed to be strictly positive. Hence, the holomorphic family $A[B,D^{-z}]$ is of the form *zR*(*z*) for some holomorphic family *R*(*z*). As ${{\,\mathrm{tr}\,}}(R(z))$ can at most have a simple pole at $z=0$, the function ${{\,\mathrm{tr}\,}}(A[B,D^{-z}])$ has no pole there and hence $\tau ([A,B])=0$.

To see (b), let ${\tilde{D}}\in \Psi ^{{\tilde{r}}}(E)$ be another strictly positive Rockland differential operator of order ${\tilde{r}}>0$, and suppose *A* is of order *k*. Hence, $AD^{-z/r}-A{\tilde{D}}^{-z/{\tilde{r}}}$ is a holomorphic family of order $k-z$ which vanishes at $z=0$. As before, we conclude that ${{\,\mathrm{tr}\,}}\bigl (AD^{-z/r}-A{\tilde{D}}^{-z/{\tilde{r}}}\bigr )$ is holomorphic at $z=0$. Consequently,

$$\begin{aligned} {{\,\mathrm{res}\,}}_{z=0}{{\,\mathrm{tr}\,}}(AD^{-z/r})={{\,\mathrm{res}\,}}_{z=0}{{\,\mathrm{tr}\,}}(A{\tilde{D}}^{-z/{\tilde{r}}}). \end{aligned}$$

Equivalently, $$r\cdot {{\,\mathrm{res}\,}}_{z=0}{{\,\mathrm{tr}\,}}(AD^{-z})={\tilde{r}}\cdot {{\,\mathrm{res}\,}}_{z=0}{{\,\mathrm{tr}\,}}(A{\tilde{D}}^{-z})$$.

To see (c), observe that for operators *A* of order less than $-n$ the zeta function $\zeta _A(z)$ is clearly analytic at $z=0$ and hence has no residue there.

Part (d) follows immediately from the formula for the residue in (86).

To see (e), suppose $B\in \Gamma ^\infty ({{\,\mathrm{end}\,}}(E))$ and observe that we clearly have

$$\begin{aligned} k_{BD^{(j-n)/r}D^{-z}}(x,x)=B(x)k_{D^{(j-n)/r-z}}(x,x), \end{aligned}$$

for all $x\in M$. Using Theorem 2, we obtain

$$\begin{aligned} {{\,\mathrm{res}\,}}_{z=0}\left( k_{BD^{(j-n)/r}D^{-z}}(x,x)\right) =B(x){{\,\mathrm{res}\,}}_{z=(n-j)/r}\left( k_{D^{-z}}(x,x)\right) =\frac{B(x)q_j(x)}{\Gamma ((n-j)/r)}, \end{aligned}$$

see Eq. 8. Applying ${{\,\mathrm{tr}\,}}_E$ and integrating over $x\in M$, we obtain (90).

The assertion about $\mu (f)$ in (f) follows from (90) by specializing to the multiplication operator $B=f$, where $f\in C^\infty (M)$, and observing that ${{\,\mathrm{tr}\,}}_E(q_0(x))>0$ at each $x\in M$, for we have $q_0(x)>0$ according to Theorem 1. Hence, $\mu$ is a positive functional.

The last statement about $\tau (D^{-\frac{n}{r}})$ in (g) follows from (90) by specializing to $A={{\,\mathrm{id}\,}}_E$ and using $\int _M{{\,\mathrm{tr}\,}}_E(q_0)=a_0>0$, see Corollary 1. In particular, $\tau$ is non-trivial. $\square$

### Remark 6

Using (84), we may identify the logarithmic term $p_A=p_A|_M$ associated with a pseudodifferential operator $A\in \Psi ^\infty (E)$, see Corollary 6(d), as an ${{\,\mathrm{end}\,}}(E)$ valued density on *M*. This Wodzicki density is intrinsic to *A*, i.e., it does not depend on the exponential coordinates used for the asymptotic expansion. The independence follows from the evident formula $p_{BA}=Bp_A$ for all $B\in \Gamma ^\infty ({{\,\mathrm{end}\,}}(E))$, the expression for the residue in Corollary 6(d), and the intrinsic definition of the residue using *D*.

In case of a trivially filtered manifold *M*, the trace $\tau$ is the non-commutative residue which was introduced in Wodzicki and Guillemin [29, 58], with many applications including to index theory [18]. In Connes [17], the non-commutative residue is shown to coincide with Dixmier trace for classical pseudodifferential operators of order $-n$. We expect such a relationship to hold also in Heisenberg calculus extending part (f) of Corollary 6.

## Weyl’s Law for Rumin–Seshadri Operators

In this section, we will work out the eigenvalue asymptotics for Rumin–Seshadri operators associated with Rockland sequences of differential operators whose Heisenberg principle symbol sequence is the same at each point. More precisely, we will assume that the principle symbol sequence, the $L^2$ inner product, and the volume density on any two osculating groups are simultaneously isometric. In this situation, the constant appearing in Weyl’s law is the product of the volume of the underlying manifold with a constant depending on the isometry class of the principal symbol sequence, see Corollary 7. Similar formulas for CR and contact geometry can be found in [43, Sect. 6].

In the second part of this section, we will specialize to a particular parabolic geometry in five dimensions associated with the exceptional Lie group $G_2$. Every irreducible representation of $G_2$ gives rise to a Rockland sequence of differential operators known as a (curved) BGG sequence [13]. Moreover, there is a distinguished class of fiberwise Hermitian inner products such that the Heisenberg principal symbol sequences, at any two points, are indeed isometric. In this situation, the constant in Weyl’s law is universal, depending only on the representation of $G_2$, see Corollary 8.

### Rumin–Seshadri Operators

Let $E_i$ be vector bundles over a closed filtered manifold *M*. Consider a sequence of differential operators

91 $$\begin{aligned} \cdots \rightarrow \Gamma ^\infty (E_{i-1})\xrightarrow {A_{i-1}}\Gamma ^\infty (E_i)\xrightarrow {A_i}\Gamma ^\infty (E_{i+1})\rightarrow \cdots \end{aligned}$$

where $A_i\in \mathcal {D}\mathcal {O}^{r_i}(E_i,E_{i+1})$ is of Heisenberg order at most $r_i\ge 1$. Recall that such a sequence is called Rockland if the Heisenberg principal symbol sequence

$$\begin{aligned} \cdots \rightarrow \mathcal {H}_\infty \otimes E_{i-1,x}\xrightarrow {\pi (\sigma _x^{r_{i-1}}(A_{i-1}))}\mathcal {H}_\infty \otimes E_{i,x}\xrightarrow {\pi (\sigma ^{r_i}_x(A_i))}\mathcal {H}_\infty \otimes E_{i+1,x}\rightarrow \cdots \end{aligned}$$

is (weakly) exact, for each $x\in M$ and every non-trivial irreducible unitary representation $\pi :\mathcal {T}_xM\rightarrow U(\mathcal {H})$ on a Hilbert space $\mathcal {H}$, see [21, Definition 2.14]. Here $\mathcal {H}_\infty$ denotes the subspace of smooth vectors.

Fix a volume density $\mathrm{d}x$ on *M*. Moreover, let $h_i$ be a fiberwise Hermitian metric on $E_i$, and consider the associated standard $L^2$-inner product

92 $$\begin{aligned} \langle \!\langle \psi _1,\psi _2\rangle \!\rangle _{L^2(E_i)}:=\int _Mh_i(\psi _1,\psi _2)\mathrm{d}x, \end{aligned}$$

where $\psi _1,\psi _2\in \Gamma ^\infty (E_i)$. Let $A_i^*\in \mathcal {D}\mathcal {O}^{r_i}(E_{i+1},E_i)$ denote the formal adjoint characterized by $\langle \!\langle A_i^*\phi ,\psi \rangle \!\rangle _{L^2(E_i)}=\langle \!\langle \phi ,A_i\psi \rangle \!\rangle _{L^2(E_{i+1})}$ for all $\psi \in \Gamma ^\infty (E_i)$ and $\phi \in \Gamma ^\infty (E_{i+1})$, cf. [21, Remark 2.5].

Assume $r_i\ge 1$ and choose positive integers $s_i$ such that

93 $$\begin{aligned} r_{i-1}s_{i-1}=r_is_i=:\kappa . \end{aligned}$$

It is easy to see that the Rumin–Seshadri operator $\Delta _i\in \mathcal {D}\mathcal {O}^{2\kappa }(E_i)$ defined by

94 $$\begin{aligned} \Delta _i:=\bigl (A_{i-1}A_{i-1}^*\bigr )^{s_{i-1}}+\bigl (A_i^*A_i\bigr )^{s_i} \end{aligned}$$

is Rockland, see [47] and [21, Lemma 2.18]. Clearly, $\Delta _i$ is formally self-adjoint and non-negative.

#### Definition 5

(*Model sequence*)

- A *model sequence* consists of a finite dimensional graded nilpotent Lie algebra $\mathfrak {n}$, a volume density $\mu \in |\Lambda _\mathfrak {n}|$, finite dimensional Hermitian vector spaces $F_i$, integers $r_i\ge 1$, and $B_i\in \mathcal {U}_{-r_i}(\mathfrak {n})\otimes \hom (F_i,F_{i+1})$. A model sequence gives rise to a sequence of left invariant homogeneous differential operators on the corresponding simply connected Lie group *N*,
  $$\begin{aligned} \cdots \rightarrow C^\infty (N,F_{i-1})\xrightarrow {B_{i-1}}C^\infty (N,F_i)\xrightarrow {B_i}C^\infty (N,F_{i+1})\rightarrow \cdots , \end{aligned}$$
  and provides left invariant $L^2$ inner products on the spaces $C^\infty (N,F_i)$ which are induced by the invariant volume density on *N* corresponding to $\mu$ and the Hermitian inner products on $F_i$, cf. (4).
- A model sequence is called *Rockland* if
  $$\begin{aligned} \cdots \rightarrow \mathcal {H}_\infty \otimes F_{i-1}\xrightarrow {\pi (B_{i-1})}\mathcal {H}_\infty \otimes F_i\xrightarrow {\pi (B_i)}\mathcal {H}_\infty \otimes F_{i+1}\rightarrow \cdots \end{aligned}$$
  is exact for every non-trivial irreducible unitary representation $\pi :N\rightarrow U(\mathcal {H})$ where $\mathcal {H}_\infty$ denotes the subspace of smooth vectors.
- Two model sequences, $\bigl (\mathfrak {n},\mu ,B_i\in \mathcal {U}_{-r_i}(\mathfrak {n})\otimes \hom (F_i,F_{i+1})\bigr )$ and $\bigl (\mathfrak {n}',\mu ',B_i'\in \mathcal {U}_{-r_i'}(\mathfrak {n}')\otimes \hom (F_i',F_{i+1}')\bigr )$ are said to be *isometric* if $r_i=r_i'$, and there exists an isomorphism of graded Lie algebras $\dot{\varphi }:\mathfrak {n}\rightarrow \mathfrak {n}'$ mapping $\mu$ to $\mu '$, and there exist unitary isomorphisms $\phi _i:F_i\rightarrow F_i'$ such that the induced isomorphisms
  $$\begin{aligned} \dot{\varphi }\otimes \hom (\phi _i^{-1},\phi _{i+1}):\mathcal {U}_{-r_i}(\mathfrak {n})\otimes \hom (F_i,F_{i+1})\rightarrow \mathcal {U}_{-r_i'}(\mathfrak {n}')\otimes \hom (F_i',F_{i+1}') \end{aligned}$$
  map $B_i$ to $B_i'$, for all *i*.

If $E_i$ are Hermitian vector bundles over a filtered manifold *M* equipped with a volume density, then the Heisenberg principal symbols of a sequence of differential operators $A_i\in \mathcal {D}\mathcal {O}^{r_i}(E_i,E_{i+1})$ give rise to a model sequence

95 $$\begin{aligned} \bigl (\mathfrak t_xM,\mu _x,\sigma ^{r_i}_x(A_i)\in \mathcal {U}_{-r_i}(\mathfrak t_xM)\otimes \hom (E_{x,i},E_{x,i+1})\bigr ) \end{aligned}$$

at every point $x\in M$. Here $\mu _x\in |\Lambda _{\mathfrak t_xM}|$ denotes the volume density induced by $\mathrm{d}x$ via the canonical isomorphism $|\Lambda _{\mathfrak t_xM}|=|\Lambda _{T_xM}|$.

The following generalizes [43, Proposition 6.1.5].

#### Corollary 7

If $\bigl (\mathfrak {n},\mu ,B_i\in \mathcal {U}_{-r_i}(\mathfrak {n})\otimes \hom (F_i,F_{i+1})\bigr )$ is a Rockland model sequence, see Definition 5, then there exist constants $\alpha _i>0$ with the following property. Suppose $E_i$ are Hermitian vector bundles over a closed filtered manifold *M*, let $\mathrm{d}x$ be a volume density on *M*, and suppose $A_i\in \mathcal {D}\mathcal {O}^{r_i}(E_i,E_{i+1})$ is a sequence of differential operators such that the Heisenberg principal symbol sequence, see (95), is isometric to the model sequence at each point $x\in M$. Moreover, fix positive integers $s_i$ as in (93), and let $\Delta _i\in \mathcal {D}\mathcal {O}^{2\kappa }(E_i)$ denote the associated Rumin–Seshadri operators, see (92) and (94). Then, as $\lambda \rightarrow \infty$,

96 $$\begin{aligned} \sharp \left\{ \begin{array}{c}\text {eigenvalues of }\Delta _i \text { less than }\lambda \\ \text {counted with multiplicities}\end{array}\right\} \sim \alpha _i\cdot {{\,\mathrm{vol}\,}}(M)\lambda ^{n/2\kappa }. \end{aligned}$$

Here ${{\,\mathrm{vol}\,}}(M):=\int _M\mathrm{d}x$, and *n* denotes the homogeneous dimension of $\mathfrak {n}$.

#### Proof

Let $x\in M$. By assumption, there exists an isomorphism of graded nilpotent Lie algebras $\dot{\varphi }:\mathfrak t_xM\rightarrow \mathfrak {n}$ mapping the volume density induced by $\mathrm{d}x$ on $\mathfrak t_xM$ to the volume density $\mu$ on $\mathfrak {n}$, and there exist unitary isomorphisms of vector spaces $\phi _i:E_{x,i}\rightarrow F_i$ such that the induced isomorphisms

$$\begin{aligned} \dot{\varphi }\otimes \hom (\phi _i^{-1},\phi _{i+1}):\mathcal {U}_{-r_i}(\mathfrak t_xM)\otimes \hom (E_{x,i},E_{x,i+1})\rightarrow \mathcal {U}_{-r_i}(\mathfrak {n})\otimes \hom (F_i,F_{i+1}) \end{aligned}$$

map $\sigma ^{r_i}_x(A_i)$ to $B_i$. We conclude that the induced isomorphism

$$\begin{aligned} \dot{\varphi }\otimes \hom (\phi _i^{-1},\phi _i):\mathcal {U}_{-2\kappa }(\mathfrak t_xM)\otimes {{\,\mathrm{end}\,}}(E_{x,i})\rightarrow \mathcal {U}_{-2\kappa }(\mathfrak {n})\otimes {{\,\mathrm{end}\,}}(F_i) \end{aligned}$$

maps $\sigma ^{2\kappa }_x(\Delta _i)$ to $C_i:=(B_{i-1}B_{i-1}^*)^{s_{i-1}}+(B_i^*B_i)^{s_i}$. Hence, the fundamental solutions of the heat equation are related by

97 $$\begin{aligned} k_t^{\sigma ^{2\kappa }_x(\Delta _i)}=\varphi ^*\bigl (\phi _i^{-1}k_t^{C_i}\phi _i\bigr ) \end{aligned}$$

where $\varphi :\mathcal {T}_xM\rightarrow N$ denotes the isomorphism of Lie groups integrating $\dot{\varphi }$. Let $\alpha _i>0$ the unique number such that

98 $$\begin{aligned} {{\,\mathrm{tr}\,}}_{F_i}\bigl (k^{C_i}_1(o_N)\bigr )=\alpha _i\Gamma (1+n/2\kappa )\mu . \end{aligned}$$

Note that $\alpha _i$ only depends on the model sequence and (potentially) on the integers $s_i$, but not on the actual geometry. From (97) and (65), we obtain

$$\begin{aligned} {{\,\mathrm{tr}\,}}_{E_i}\bigl (q_0^{\Delta _i}\bigr )=\alpha _i\Gamma (1+n/2\kappa )\mathrm{d}x. \end{aligned}$$

where $q_0^{\Delta _i}\in \Gamma ^\infty \bigl ({{\,\mathrm{end}\,}}(E_i)\otimes |\Lambda _M|\bigr )$ is as in Theorem 1. Hence, for the corresponding $a_0^{\Delta _i}=\int _M{{\,\mathrm{tr}\,}}_{E_i}\bigl (q_0^{\Delta _i}\bigr )$, see Corollary 1, we obtain

$$\begin{aligned} a_0^{\Delta _i}=\alpha _i\Gamma (1+n/2\kappa ){{\,\mathrm{vol}\,}}(M). \end{aligned}$$

The statement in (96) thus follows from Corollary 3.

It remains to show that the constants $\alpha _i$ do not depend on the choice of positive integers $s_i$ as in (93). To this end, suppose $u\in \mathbb {N}$ and consider $s_i':=us_i$. Then $r_{i-1}s_{i-1}'=r_is_i'=\kappa '$ with $\kappa '=u\kappa$, cf. (93). Since $A_i$ is a Rockland sequence, we have $\sigma ^{k_i}_x(A_i)\sigma ^{k_{i-1}}_x(A_{i-1})=0$. Hence, $\Delta '_i:=(A_{i-1}A_{i-1}^*)^{s_{i-1}'}+(A_i^*A_i)^{s_i'}$ and $(\Delta _i)^u$ have the same Heisenberg principal symbol, i.e., $\sigma ^{2\kappa '}_x(\Delta _i')=\sigma _x^{2u\kappa }((\Delta _i)^u)$, and thus the constants in their Weyl asymptotics coincide, see Corollary 3, Remark 2, and (65). Clearly, the number of eigenvalues less than $\lambda$ of $(\Delta _i)^u$ coincides with the number of eigenvalues of $\Delta _i$ which are smaller than $\lambda ^{1/u}$. From (96) we thus obtain

$$\begin{aligned} \sharp \left\{ \begin{array}{c}\text {eigenvalues of }\Delta _i'\text { less than }\lambda \\ \text {counted with multiplicities}\end{array}\right\} \sim \alpha _i\cdot {{\,\mathrm{vol}\,}}(M)\lambda ^{n/2\kappa '}. \end{aligned}$$

We conclude that the constant $\alpha _i$ does not depend on the choice of $s_i$. $\square$

### Generic Rank-Two Distributions in Dimension Five

In the remaining part of this section, we will specialize to a particular geometry in five dimensions and discuss certain natural Rockland sequences over it, known as curved BGG sequences, which have been constructed by Čap, Slovák and Souček, see [13].

Let *M* be 5-manifold, and suppose $H\subseteq TM$ is a smooth subbundle of rank two. Recall that *H* is said to be of Cartan type [8] if it is bracket generating with growth vector (2, 3, 5). More precisely, *H* is of Cartan type if every point in *M* admits an open neighborhood *U* and there exist sections $X,Y\in \Gamma ^\infty (H|_U)$ such that *X*, *Y*, [*X*, *Y*], [*X*, [*X*, *Y*]], [*Y*, [*X*, *Y*]] is a frame of $TM|_U$. Putting $T^{-1}M:=H$ and denoting the rank-three bundle spanned by Lie brackets of sections of *H* by $T^{-2}M:=[H,H]$, the 5-manifold *M* thus becomes a filtered manifold,

99 $$\begin{aligned} TM=T^{-3}M\supseteq T^{-2}M\supseteq T^{-1}M\supseteq T^0M=0. \end{aligned}$$

The osculating algebras $\mathfrak t_xM$ are all isomorphic to the graded nilpotent Lie algebra $\mathfrak {n}=\mathfrak {n}_{-3}\oplus \mathfrak {n}_{-2}\oplus \mathfrak {n}_{-1}$, uniquely characterized (up to isomorphism) by the fact that $\mathfrak {n}_{-1}$ admits a basis $\xi ,\eta$ such that $[\xi ,\eta ]$ is a basis of $\mathfrak {n}_{-2}$ and $[\xi ,[\xi ,\eta ]],[\eta ,[\xi ,\eta ]]$ forms a basis of $\mathfrak {n}_{-3}$.

Rank-two distributions of Cartan type are also known as generic rank two distributions in dimension five, see [10, 31, 32, 48, 49], the condition on *H* being open with respect to the $C^2$-topology. Their history can be traced back to Cartan’s celebrated “five variables paper” [14]. Whether a 5-manifold admits a Cartan distribution is well understood in the open case, see [20, Theorem 2]. For closed 5-manifolds, however, this problem remains open and has served as a major motivation to develop the analysis in this paper.

A rank-two distribution of Cartan type can equivalently be described as regular normal Cartan geometry of type (*G*, *P*) where *G* denotes the split real form of the exceptional Lie group $G_2$ and *P* denotes a maximal parabolic subgroup corresponding to the shorter root, see [14, 48] and [11, Theorem 3.1.14 and Sect. 4.3.2]. The Lie algebra of *G* admits a grading,

100 $$\begin{aligned} \mathfrak {g}=\mathfrak {g}_{-3}\oplus \mathfrak {g}_{-2}\oplus \mathfrak {g}_{-1}\oplus \mathfrak {g}_0\oplus \mathfrak {g}_1\oplus \mathfrak {g}_2\oplus \mathfrak {g}_3, \end{aligned}$$

i.e., $[\mathfrak {g}_i,\mathfrak {g}_j]\subseteq \mathfrak {g}_{i+j}$ for all *i* and *j*, such that

$$\begin{aligned} P=\{g\in G\mid \forall i:{{\,\mathrm{Ad}\,}}_g(\mathfrak {g}^i)\subseteq \mathfrak {g}^i\}, \end{aligned}$$

where $\mathfrak {g}^i:=\bigoplus _{i\le j}\mathfrak {g}_j$ denotes the associated filtration. The Lie algebra of *P* is $\mathfrak {p}=\mathfrak {g}_0\oplus \mathfrak {g}_1\oplus \mathfrak {g}_2\oplus \mathfrak {g}_3$. The corresponding Levi group,

101 $$\begin{aligned} G_0=\{g\in G\mid \forall i:{{\,\mathrm{Ad}\,}}_g(\mathfrak {g}_i)=\mathfrak {g}_i\}\cong {{\,\mathrm{GL}\,}}(2,\mathbb {R}), \end{aligned}$$

has Lie algebra $\mathfrak {g}_0\cong {\mathfrak {g}}{\mathfrak {l}}(2,\mathbb {R})$. Furthermore, $\mathfrak {p}_+=\mathfrak {g}_1\oplus \mathfrak {g}_2\oplus \mathfrak {g}_3$ is a nilpotent ideal in $\mathfrak {p}$ and $P_+:=\exp (\mathfrak {p}_+)$ is a closed normal subgroup in *P* such that the inclusion $G_0\subseteq P$ induces a natural isomorphism of groups, $G_0=P/P_+$. The graded nilpotent Lie algebra $\mathfrak {g}_-:=\mathfrak {g}_{-3}\oplus \mathfrak {g}_{-2}\oplus \mathfrak {g}_{-1}$ is isomorphic to $\mathfrak {n}$.

The Cartan geometry consists of a principal *P*-bundle $\mathcal {G}\rightarrow M$ and a regular Cartan connection $\omega \in \Omega ^1(\mathcal {G};\mathfrak {g})$ satisfying a normalization condition, see [11]. The Cartan connection induces an isomorphism $TM=\mathcal {G}\times _P\mathfrak {g}/\mathfrak {p}$. By regularity, the filtration on *TM*, see (99), coincides with the filtration induced by the filtration $\mathfrak {g}/\mathfrak {p}=\mathfrak {g}^{-3}/\mathfrak {p}\supseteq \mathfrak {g}^{-2}/\mathfrak {p}\supseteq \mathfrak {g}^{-1}/\mathfrak {p}\supseteq \mathfrak {g}^0/\mathfrak {p}=0$. Note that $P_+$ acts trivially on the associated graded ${{\,\mathrm{gr}\,}}(\mathfrak {g}/\mathfrak {p})$, and the inclusion $\mathfrak {g}_-\subseteq \mathfrak {g}$ induces an isomorphism of $G_0=P/P_+$ modules, $\mathfrak {g}_-={{\,\mathrm{gr}\,}}(\mathfrak {g}/\mathfrak {p})$. Regularity also implies that the induced isomorphism $\mathfrak tM={{\,\mathrm{gr}\,}}(TM)=\mathcal {G}\times _P{{\,\mathrm{gr}\,}}(\mathfrak {g}/\mathfrak {p})=\mathcal {G}_0\times _{G_0}\mathfrak {g}_-$ is an isomorphism of bundles of graded nilpotent Lie algebras. Here $\mathcal {G}_0:=\mathcal {G}/P_+$ is considered as a principal $G_0$-bundle over *M*.

The canonically associated Cartan geometry permits to construct natural sequences of differential operators over *M* known as curved BGG sequences, see [13] and [9, 12]. Every finite dimensional complex representation $\rho :G\rightarrow {{\,\mathrm{GL}\,}}(\mathbb {E})$ gives rise to a sequence of natural differential operators,

102 $$\begin{aligned} 0\rightarrow \Gamma (E_0)\xrightarrow {D_0}\Gamma (E_1)\xrightarrow {D_1}\Gamma (E_2)\xrightarrow {D_2}\Gamma (E_3)\xrightarrow {D_3}\Gamma (E_4)\xrightarrow {D_5}\Gamma (E_5)\rightarrow 0\qquad \quad \end{aligned}$$

where $E_i:=\mathcal {G}_0\times _{G_0}H^i(\mathfrak {g}_-;\mathbb {E})$ and $H^i(\mathfrak {g}_-;\mathbb {E})$ denotes the *i*-th Lie algebra cohomology with coefficients in the representation of $\mathfrak {g}_-$ obtained by restricting $\rho$. In [21, Corollary 4.23 and Example 4.24] it has been shown that this is a Rockland sequence for every irreducible representation $\rho$. We will denote the Heisenberg order of $D_i$ by $k_i$.

Let $\theta$ be a Cartan involution on $\mathfrak {g}$ such that

103 $$\begin{aligned} \theta (\mathfrak {g}_i)=\mathfrak {g}_{-i} \end{aligned}$$

for all *i*, cf. [11, Proof of Proposition 3.3.1]. Denoting the Killing form of $\mathfrak {g}$ by *B*, we obtain a Euclidean inner product on $\mathfrak {g}$, given by $B_\theta (X,Y):=-B(X,\theta (Y))$ where $X,Y\in \mathfrak {g}$. The summands in the decomposition (100) are orthogonal with respect to $B_\theta$. Let $\Theta$ denote the global Cartan involution on *G* corresponding to $\theta$. Then $K:=\{g\in G:\Theta (g)=g\}\cong \bigl ({{\,\mathrm{SU}\,}}(2)\times {{\,\mathrm{SU}\,}}(2)\bigr )/\mathbb {Z}_2$ is a maximal compact subgroup of *G*, and $B_\theta$ is invariant under *K*. Moreover, $\Theta$ restricts to a Cartan involution on $G_0$, and $K_0:=\{g\in G_0:\Theta (g)=g\}\cong O(2,\mathbb {R})$ is a maximal compact subgroup of $G_0$. Let *h* be a Hermitian inner product on $\mathbb {E}$ such that

104 $$\begin{aligned} h(\rho '(X)v,w)=-h(v,\rho '(\theta (X))w) \end{aligned}$$

for all $X\in \mathfrak {g}$ and $v,w\in \mathbb {E}$ where $\rho ':\mathfrak {g}\rightarrow {\mathfrak {g}}{\mathfrak {l}}(\mathbb {E})$ denotes the Lie algebra representation corresponding to $\rho$, cf. [11, Proof of Proposition 3.3.1]. In particular, *h* is invariant under the action of *K*. We equip the $G_0$-module $C^i(\mathfrak {g}_-;\mathbb {E})=\Lambda ^i\mathfrak {g}_-^*\otimes \mathbb {E}$ with the Hermitian inner product $h_i$ induced by $B_\theta$ and *h*. Note that $h_i$ is invariant under $K_0$. These inner products play an important role in the construction of the curved BGG sequences since Kostant’s codifferential, $\partial ^*:C^{i+1}(\mathfrak {g}_-;\mathbb {E})\rightarrow C^i(\mathfrak {g}_-;\mathbb {E})$, is adjoint to the Chevalley–Eilenberg codifferential $\partial :C^i(\mathfrak {g}_-;\mathbb {E})\rightarrow C^{i+1}(\mathfrak {g}_-;\mathbb {E})$ with respect to $h_i$, see [11, Proposition 3.3.1]. The induced $K_0$-invariant Hermitian inner product on $H^i(\mathfrak {g}_-;\mathbb {E})$ will also be denoted by $h_i$. We let $\mu \in |\Lambda _{\mathfrak {g}_-}|$ denote the volume density associated with the Euclidean inner product on $\mathfrak {g}_-$ induced by $B_\theta$. Clearly, $\mu$ is $K_0$-invariant.

To obtain Hermitian inner products on the bundles $E_i$, we choose a reduction of the structure group of $\mathcal {G}_0\rightarrow M$ to $K_0\subseteq G_0$. This is a principal $K_0$-bundle $\mathcal {K}_0\rightarrow M$ together with a $K_0$-equivariant bundle map $\mathcal {K}_0\subseteq \mathcal {G}_0$. This choice amounts to fixing a fiberwise Euclidean metric on the rank-two bundle *H*. It provides an isomorphism of principal $G_0$-bundles, $\mathcal {G}_0=\mathcal {K}_0\times _{K_0}G_0$, and thus

105 $$\begin{aligned} E_i=\mathcal {G}_0\times _{G_0}H^i(\mathfrak {g}_-;\mathbb {E})=\mathcal {K}_0\times _{K_0}H^i(\mathfrak {g}_-;\mathbb {E}). \end{aligned}$$

Moreover,

$$\begin{aligned} {{\,\mathrm{gr}\,}}(TM)=\mathcal {G}_0\times _{G_0}\mathfrak {g}_-=\mathcal {K}_0\times _{K_0}\mathfrak {g}_-. \end{aligned}$$

We equip $E_i$ with the fiberwise Hermitian metric induced from the $K_0$-invariant Hermitian metric on $H^i(\mathfrak {g}_-;\mathbb {E})$, and we equip *M* with the volume density $\mathrm{d}x$ induced from the $K_0$-invariant volume density $\mu$ on $\mathfrak {g}_-$.

#### Corollary 8

Let *M* be a closed 5-manifold equipped with a rank-two distribution of Cartan type. Moreover, let $\rho$ be an irreducible complex representation of the exceptional Lie group $G_2$, consider the associated curved BGG sequence (102), and let $\Delta _i$ denote the corresponding Rumin–Seshadri operator, see (94), constructed using the volume density $\mathrm{d}x$ on *M* and the fiberwise Hermitian metrics on $E_i$ described above. Then, as $\lambda \rightarrow \infty$,

106 $$\begin{aligned} \sharp \left\{ \begin{array}{c}\text {eigenvalues of }\Delta _i\text { less than }\lambda \\ \text {counted with multiplicities}\end{array}\right\} \sim \alpha _i(\rho )\cdot {{\,\mathrm{vol}\,}}(M)\lambda ^{5/\kappa }, \end{aligned}$$

where $\alpha _i(\rho )>0$ and ${{\,\mathrm{vol}\,}}(M)=\int _M\mathrm{d}x$. The constant $\alpha _i(\rho )$ does not depend on *M* or the distribution *H*, nor does it depend on the Cartan involution $\theta$ satisfying (103) or the Hermitian inner product *h* satisfying (104) or the reduction of structure group of $\mathcal {G}_0$ along $K_0\subseteq G_0$, and it is also independent of the choice of positive integers $s_i$ as in (93); it only depends on *i* and the representation $\rho$.

#### Proof

Fix $x\in M$. Every $G_0$-equivariant identification $(\mathcal {G}_0)_x\cong G_0$ induces an isomorphism of graded nilpotent Lie algebras $\dot{\varphi }:\mathfrak t_xM\rightarrow \mathfrak {g}_-$ and isomorphisms of vector spaces $\phi _i:E_{i,x}\rightarrow H^i(\mathfrak {g}_-;\mathbb {E})$ which intertwine the principal Heisenberg symbol $\sigma ^{k_i}_x(D_i)$ with $D_i^{G_-}$, the corresponding left invariant operator on the nilpotent Lie group $G_-=\exp (\mathfrak {g}_-)$. If the frame $(\mathcal {G}_0)_x\cong G_0$ is induced from a $K_0$-equivariant identification $(\mathcal {K}_0)_x\cong K_0$, then the isomorphisms $\phi _i$ are unitary and $\dot{\varphi }$ maps the volume density $\mu _x$ on $\mathfrak t_xM$ induced by $\mathrm{d}x$ to the volume density $\mu$ on $\mathfrak {g}_-$. Hence, at every point $x\in M$, the principal symbol sequence

$$\begin{aligned} \bigl (\mathfrak t_xM,\mu _x,\sigma ^{k_i}_x(D_i)\in \mathcal {U}_{-k_i}(\mathfrak t_xM)\otimes \hom (E_{i,x},E_{i+i,x})\bigr ) \end{aligned}$$

is isometric to the model sequence

$$\begin{aligned} \bigl (\mathfrak {g}_-,\mu ,D_i^{G_-}\in \mathcal {U}_{-k_i}(\mathfrak {g}_-)\otimes \hom (H^i(\mathfrak {g}_-;\mathbb {E}),H^{i+1}(\mathfrak {g}_-;\mathbb {E}))\bigr ), \end{aligned}$$

cf. Definition 5. According to Corollary 7 there exists $\alpha _i(\rho )>0$ such that (106) holds, and this constant does not depend on the choice of $s_i$ as in (93).

It remains to show that $\alpha _i(\rho )$ does not depend on $\theta$ or *h*. To this end, suppose $\tilde{\theta }$ is another Cartan involution on $\mathfrak {g}$ such that $\tilde{\theta }(\mathfrak {g}_i)=\mathfrak {g}_{-i}$, for all *i*, cf. (103). Then there exists $g\in G$ such that $\tilde{\theta }={{\,\mathrm{Ad}\,}}_g^{-1}\circ \,\theta \circ {{\,\mathrm{Ad}\,}}_g={{\,\mathrm{Ad}\,}}_{g^{-1}\Theta (g)}\circ \,\theta$. In view of the Cartan decomposition, we may write $g=k\exp (X)$ with $k\in G$ and $X\in \mathfrak {g}$ such that $\Theta (k)=k$ and $\theta (X)=-X$. Hence, $g^{-1}\Theta (g)=\exp (-2X)$, and we obtain $\tilde{\theta }={{\,\mathrm{Ad}\,}}_{\exp (-2X)}\circ \,\theta$. Since $(\tilde{\theta }\theta ^{-1})(\mathfrak {g}_i)=\mathfrak {g}_i$, we have $\exp (-2X)\in G_0$, see (101). Using $\theta (X)=-X$, we actually conclude $X\in \mathfrak {g}_0$, hence $g_0:=\exp (X)\in G_0$, and

107 $$\begin{aligned} \tilde{\theta }={{\,\mathrm{Ad}\,}}_{g_0}^{-1}\circ \,\theta \circ {{\,\mathrm{Ad}\,}}_{g_0}. \end{aligned}$$

For the corresponding global Cartan involution $\tilde{\Theta }$ we get $\tilde{\Theta }(g)=g_0^{-1}\Theta (g_0gg_0^{-1})g_0$, for all $g\in G$, and thus the corresponding maximal compact subgroups are related by ${\tilde{K}}=\{g\in G:\tilde{\Theta }(g)=g\}=g_0^{-1}Kg_0$ and

108 $$\begin{aligned} {\tilde{K}}_0=\{g\in G_0:\tilde{\Theta }(g)=g\}=g_0^{-1}K_0g_0. \end{aligned}$$

Furthermore, suppose ${\tilde{h}}$ is another Hermitian inner product on $\mathbb {E}$ such that ${\tilde{h}}(\rho '(X)v,w)=-{\tilde{h}}(v,\rho '(\tilde{\theta }(X))w)$ for all $X\in \mathfrak {g}$ and $v,w\in \mathbb {E}$, cf. (104). Using $\rho '({{\,\mathrm{Ad}\,}}_{g_0}(X))=\rho (g_0)\rho '(X)\rho (g_0)^{-1}$ and (107) as well as (104), we obtain the relation $h(\rho (g_0)\rho '(X)v,\rho (g_0)w)=-h(\rho (g_0)v,\rho (g_0)\rho '(\tilde{\theta }(X))w)$. Hence, by Schur’s lemma, there exists a constant $C>0$ such that, for all $v,w\in \mathbb {E}$,

109 $$\begin{aligned} {\tilde{h}}(v,w)=C\cdot h\bigl (\rho (g_0)v,\rho (g_0)w\bigr ). \end{aligned}$$

By invariance of the Killing form and (107), we have

110 $$\begin{aligned} B_{\tilde{\theta }}(X,Y)=B_\theta \bigl ({{\,\mathrm{Ad}\,}}_{g_0}(X),{{\,\mathrm{Ad}\,}}_{g_0}(Y)\bigr ). \end{aligned}$$

Hence, the ${\tilde{K}}_0$-invariant Hermitian inner product ${\tilde{h}}_i$ induced by $B_{\tilde{\theta }}$ and ${\tilde{h}}$ on $H^i(\mathfrak {g}_-;\mathbb {E})$ is related to the $K_0$-invariant Hermitian inner product $h_i$ via

111 $$\begin{aligned} {\tilde{h}}_i(\xi ,\eta )=C\cdot h_i(g_0\cdot \xi ,g_0\cdot \eta ) \end{aligned}$$

where $\xi ,\eta \in H^i(\mathfrak {g}_-;\mathbb {E})$ and $g_0\cdot \xi$ denotes the $G_0$-action.

Suppose, moreover, $\tilde{\mathcal {K}}_0\rightarrow \mathcal {G}_0$ is a reduction of the structure group along the inclusion ${\tilde{K}}_0\subseteq G_0$. Let $\mathcal {K}_0$ denote the principal $K_0$-bundle with underlying manifold $\tilde{\mathcal {K}}_0$ where the right action by $k\in K_0$ is given by the principal ${\tilde{K}}_0$-action of $g_0^{-1}kg_0$ on $\tilde{\mathcal {K}}_0$, see (108). We consider the reduction of structure group $\mathcal {K}_0\rightarrow \mathcal {G}_0$ along the inclusion $K_0\subseteq G_0$ obtained by composing the reduction $\tilde{\mathcal {K}}_0\rightarrow \mathcal {G}_0$ with the diffeomorphism $\mathcal {G}_0\rightarrow \mathcal {G}_0$ provided by right action with $g_0^{-1}$. Using (111), we conclude that via the canonical identifications

$$\begin{aligned} \tilde{\mathcal {K}}_0\times _{{\tilde{K}}_0}H^i(\mathfrak {g}_-;\mathbb {E})=E_i=\mathcal {K}_0\times _{K_0}H^i(\mathfrak {g}_-;\mathbb {E}), \end{aligned}$$

cf. (105), the Hermitian inner products on $E_i$ induced by ${\tilde{h}}_i$ and $h_i$, respectively, differ by the constant *C*. Similarly, using (110), we see that the induced volume densities on *M* coincide. This implies that the corresponding $L_2$-inner products on $\Gamma (E_i)$ differ by a constant which is independent of *i*, hence they give rise to the same formally adjoint operators.

Summarizing, we see that the Rumin–Seshadri operators associated with $\tilde{\theta }$, ${\tilde{h}}$, and a reduction $\tilde{\mathcal {K}_0}\rightarrow \mathcal {G}_0$ coincide with the Rumin–Seshadri operators associated with $\theta$, *h*, and the reduction $\mathcal {K}_0\rightarrow \mathcal {G}_0$ described in the previous paragraph. We conclude that the constant $\alpha _i(\rho )$ does not depend on $\theta$ or *h*. $\square$

Let us conclude this section with some remarks on the constant $\alpha _0(\rho _0)$ in Corollary 8 for the trivial representation $\rho _0$. For the nilpotent Lie group $G_-$ all irreducible unitary representations have been calculated explicitly in [24, Proposition 8]. For each $(\lambda ,\mu ,\nu )\in \mathbb {R}^3$ such that $(\lambda ,\mu )\ne (0,0)$ there is an irreducible unitary representation of $G_-$ on $L^2(\mathbb {R})$ with the standard inner product $\langle \psi _1,\psi _2\rangle =\int \bar{\psi }_1(\theta )\psi _2(\theta )d\theta$ where $d\theta$ denotes the Lebesgue measure. Moreover, these representations are mutually non-equivalent. There are further, more singular representations, but they form a set of measure zero with respect to the Plancherel measure. On the representations parametrized by $(\lambda ,\mu ,\nu )$ above, the Plancherel measure is $d\lambda \,d\mu \,d\nu$, i.e., the Lebesgue measure on $\mathbb {R}^3$, see [24, Eq. (26)]. In the representation corresponding to $(\lambda ,\mu ,\nu )$, the sub-Laplacian on $G_-$ takes the form of a Laplacian with quartic potential,

$$\begin{aligned} -\hat{\Delta }^{\lambda ,\mu ,\nu }=\frac{1}{\lambda ^2+\mu ^2}\frac{d^2}{d\theta ^2}-\frac{\bigl ((\lambda ^2+\mu ^2)^2\theta ^2+\nu \bigr )^2}{4(\lambda ^2+\mu ^2)}, \end{aligned}$$

see [7, Sect. 3.3.2]. Denoting the solution of

$$\begin{aligned} \tfrac{\partial }{\partial t}\Psi _t=-\hat{\Delta }^{\lambda ,\mu ,\nu }\Psi _t,\qquad \lim _{t\searrow 0}\Psi _t=\delta _{\bar{\theta }} \end{aligned}$$

by $\Psi ^{\lambda ,\mu ,\nu }_t(\theta ,\bar{\theta })$, the heat kernel of the sub-Laplacian at the origin in $G_-$ can be expressed in the form

$$\begin{aligned} k_t^{G_-}(o)=\int _{(\lambda ,\mu )\ne (0,0)}d\lambda \,d\mu \,d\nu \int _{-\infty }^\infty d\theta \,\Psi _t^{\lambda ,\mu ,\nu }(\theta ,\theta ), \end{aligned}$$

see [1, Corollary 29] and Theorem [7, Eq. (25)]. Integrating out the circular symmetry and using (98), we obtain the following expression for one of the constants in Corollary 8

$$\begin{aligned} \alpha _0(\rho _0)=\frac{2\pi }{5!}\int _0^\infty \mu d\mu \int _{-\infty }^\infty d\nu \int _{-\infty }^\infty d\theta \,\Psi _1^{0,\mu ,\nu }(\theta ,\theta ). \end{aligned}$$

No explicit formulas for $\Psi _t^{\lambda ,\mu ,\nu }(\theta ,\bar{\theta })$ appear to be known [7]. For some 2-step nilpotent groups, explicit formulas for the heat kernel of the sub-Laplacian can be found in [5, 19, 27].

## Footnotes

1 We could use $\Lambda =A$ and a left parametrix $\Lambda '=B$ as above. Alternatively, we may assume $R=0$, see [21, Lemma 3.16].

2 Note that there are no non-trivial formally self-adjoint and non-negative differential operators of odd Heisenberg order.

3 $\mathcal {H}$ has to be one-dimensional in this case.

4 By adding smooth cosymbols, the asymptotic terms $k_{j,z_j}$ may be chosen to have this property. They are not unique, but $p_j$ is without ambiguity, cf. [21, Lemma 3.8] or Lemma 2.
